# Plant-Based Diets Induce Transcriptomic Changes in Muscle of Zebrafish and Atlantic Salmon

**DOI:** 10.3389/fgene.2020.575237

**Published:** 2020-10-22

**Authors:** Anusha K. S. Dhanasiri, Amritha Johny, Xi Xue, Gerd M. Berge, Andre S. Bogevik, Matthew L. Rise, Christiane K. Fæste, Jorge M. O. Fernandes

**Affiliations:** ^1^Faculty of Biosciences and Aquaculture, Nord University, Bodø, Norway; ^2^Department of Paraclinical Sciences, Faculty of Veterinary Medicine, Norwegian University of Life Sciences (NMBU), Oslo, Norway; ^3^Toxinology Research Group, Norwegian Veterinary Institute, Oslo, Norway; ^4^Department of Ocean Sciences, Memorial University of Newfoundland, St. John’s, NL, Canada; ^5^Norwegian Institute of Food, Fisheries and Aquaculture Research (Nofima), Sunndalsøra, Norway; ^6^Norwegian Institute of Food, Fisheries and Aquaculture Research (Nofima), Fyllingsdalen, Norway

**Keywords:** plant-based proteins, fast muscle, pea protein concentrate, soy protein concentrate, wheat gluten, gene expression

## Abstract

With the expansion of the aquaculture industry in the last two decades, there has been a large increase in the use of plant ingredients in aquafeeds, which has created new challenges in fish growth, health and welfare. Fish muscle growth is an important trait that is strongly affected by diet, but our knowledge on the effect of plant protein-based diets on global gene expression in muscle is still scant. The present study evaluated nutrigenomic effects of the inclusion of proteins from pea, soy and wheat into aquafeeds, compared to a control diet with fishmeal as the main protein source using the zebrafish model by RNA-seq; these results were extended to an important aquaculture species by analyzing selected differentially expressed genes identified in the zebrafish model on on-growing Atlantic salmon fed with equivalent plant protein-based diets. Expression of selected Atlantic salmon paralogues of the zebrafish homologs was analyzed using paralogue-specific qPCR assays. Global gene expression changes in muscle of zebrafish fed with plant-based diets were moderate, with the highest changes observed in the soy diet-fed fish, and no change for the pea diet-fed fish compared to the control diet. Among the differentially expressed genes were *mylpfb*, *hsp90aa1.1*, *col2a1a*, and *odc1*, which are important in regulating muscle growth, maintaining muscle structure and function, and muscle tissue homeostasis. Furthermore, those genes and their paralogues were differentially expressed in Atlantic salmon fed with the equivalent percentage of soy or wheat protein containing diets. Some of these genes were similarly regulated in both species while others showed species-specific regulation. The present study expands our understanding on the molecular effects of plant ingredients in fish muscle. Ultimately, the knowledge gained would be of importance for the improved formulation of sustainable plant-based diets for the aquaculture industry.

## Introduction

Exploration of alternative feed ingredients for fishmeal and fish oil has become a necessity for sustainable growth of the aquaculture sector (Carter and Hauler, 2000; Gatlin et al., 2007; Hardy, 2010). The industry has seen a steady increase in utilization of plant-based ingredients, mainly legumes, cereal grains and oil seeds in the fish feeds throughout the past years. For instance, the Norwegian salmon industry reported a large increase in use of plant ingredients, from 10% in 1990 to 60.5% in 2016 (Ytrestøyl et al., 2015; Aas et al., 2019). This huge transition to plant-based ingredients in the diet of farmed fish has led to new challenges in fish growth, health and welfare, and product quality (Hardy, 2010). Some of these challenges have been successfully addressed through advanced feed processing technologies and extensive research on the influence of dietary incorporation of plant ingredients on health, growth and nutrient utilization (Drew et al., 2007; Gatlin et al., 2007), but many challenges remain.

Plant-based proteins in fish diets can affect growth as a consequence of their nutritional and non-nutritional characteristics (Gatlin et al., 2007). Plant proteins also have some nutritional limitations such as the presence of high amounts of carbohydrates and imbalance in amino acid profiles compared with fishmeal. Generally, they are limited in some essential amino acids such as lysine and/or methionine, and low in available phosphorus and cationic minerals that mainly exist in bound form with phytic acid (Gatlin et al., 2007). With the inclusion of plant ingredients in the diets, fish could be exposed to various phytochemicals including anti−nutritional factors (ANFs) and phytoestrogens. ANFs (e.g., fibers, enzyme inhibitors, lectins, saponins, etc.) can interfere with nutrient digestibility, absorption and utilization, ultimately negatively affecting growth and health (Francis et al., 2001; Krogdahl et al., 2010). Phytoestrogens are compounds with estrogenic activity in animals, which can have an impact on the processes regulated by estrogens (Rietjens et al., 2013). Phytoestrogens have been reported to mostly exert a positive effect on fish growth (Chakraborty et al., 2014).

Among the widely used plant-based protein sources in aquafeeds, soy protein concentrate (SPC) produced from acid alcohol extraction has become the preferred choice compared to soy bean meal due to the absence of the ANF saponins as well as low contents of trypsin inhibitors, storage globulins with antigenic properties and oligosaccharides (Drew et al., 2007; Zhou et al., 2018). Nevertheless, it has been shown that diets with SPC caused intestinal enteritis in salmonids (Escaffre et al., 2007; Penn et al., 2011). Furthermore, inclusion of SPC in diets affects growth, depending on the percentage of replacement and fish species, e.g., partial replacement (50%) in diet of Atlantic salmon, *Salmo salar* (Storebakken et al., 2000b) and total replacement (100%) in diet of rainbow trout, *Oncorhynchus mykiss* (Kaushik et al., 1995) of fishmeal with SPC caused no changes in the growth of respective fish species. In contrast, a decrease in growth was observed in Japanese flounder, *Paralichthys olivaceus* (Deng et al., 2006), and gilthead sea bream, *Sparus aurata* (Kokou et al., 2015), with 25% of fishmeal replacement and 40% of inclusion with SPC in the diets, respectively.

The inclusion of pea protein concentrate (PPC) in aquafeeds also showed its potential as an alternative to fishmeal in farmed fish (Thiessen et al., 2003; Øverland et al., 2009; Zhang et al., 2012). Production of PPC by applying dehulling and air classification processes has enhanced the protein content, reduced the starch content and lowered certain ANF such as tannins compared to pea meal (Drew et al., 2007). However, it can still contain protease inhibitors, phytic acid, α-galactosidases and saponins in considerable amounts (Drew et al., 2007). PPC caused intestinal inflammation in the Atlantic salmon, similarly to soy-induced enteritis, and reduction in growth when included at a level of 35% in the diet (Penn et al., 2011). However, another study has reported that even inclusion levels of 50% of the dietary protein did not show any unfavorable effect on growth in rainbow trout (Zhang et al., 2012).

Wheat gluten (WG) has become an attractive alternative protein source for aquafeeds in several farmed species (Apper-Bossard et al., 2013), since it has a comparatively high crude protein content than fishmeal. Moreover, WG is used as pellet binder in feed manufacturing. It is high in sulfur amino acids and glutamate but generally low in lysine compared to the other commonly used plant protein sources (Storebakken et al., 2000a; Apper-Bossard et al., 2013). In a study on rainbow trout, it was found that WG was able to replace FM completely or to a large proportion without any unfavorable effect on growth, when lysine was supplemented in the diet (Pfeffer et al., 1992; Davies et al., 1997). Furthermore, moderate FM replacement with WG did not show any effect on growth in Atlantic salmon (35%) (Storebakken et al., 2000a) and Nile tilapia, *Oreochromis niloticus* L. (15%) (Schneider et al., 2004).

Muscle growth is an important trait affected by the nutritional status and diet (Valente et al., 2013). Some previous studies have examined the transcriptomic changes linked to muscle growth and dietary manipulation in fish (Alami-Durante et al., 2010; Bower and Johnston, 2010; Ulloa et al., 2013, 2015; Valente et al., 2016). However, knowledge on the effect of plant protein-based diets on global gene expression in muscle is still scant. In the present study, we have evaluated nutrigenomic effects of the inclusion of plant proteins SPC, PPC, and WG into aquafeeds, compared to a control diet with fishmeal as the main protein source, using the zebrafish as a model. Global transcriptome changes were analyzed in fast muscle using RNA-seq technology. The analysis was extended to a commercially important species by testing selected genes from the zebrafish model on on-growing Atlantic salmon fed with custom-made feeds containing the same plant ingredients. Considering the salmonid-specific genome duplication event (Lien et al., 2016), expression of selected paralogues of respective selected genes was analyzed using paralogue-specific qPCR assays.

## Materials and Methods

### Feeding Experiments

#### Feeds

Custom-made diets for zebrafish and Atlantic salmon were produced by extrusion at the Nofima Feed Technology Centre, Fyllingsdalen, Norway as described by Johny et al. (2019). The control diet included 79.4 and 63.4% fishmeal as protein source for, respectively, zebrafish and salmon (dietary composition is presented in Supplementary Table 1). Both control diets had 12% wheat as pellet-binding component, and 4.6% of additives including monosodium phosphate-24% P, vitamins and mineral mix. Considering the high fish oil requirement of salmon, 20% fish oil was added into the salmon diets as compared to 4% in the zebrafish diets. Plant protein-based diets were prepared by replacing 30% fishmeal in the control diet with PPC, SPC, or WG as protein source. The total protein content in all diets was 50 to 59 g/100 g feed for zebrafish and 44 to 48 g/100g feed for salmon, while the lipid content ranged from 9 to 12% and from 24 to 26% for, respectively, zebrafish and salmon. The plant-based diets were not supplemented with amino acids.

#### Zebrafish Feeding Trials and Sampling

Feeding trials were conducted using four-month-old zebrafish (AB strain, mean weight 0.214 g) for 46 days at Nord University, Bodø, Norway. Sixteen fish (1:1 sex ratio) were distributed into each of four randomly assigned replicate tanks (3.5 L volume) per dietary group in a flow-through system with 20% water exchange per h (ZebTEC stand-alone toxicology rack; Tecniplast, Buguggiate, Italy). Standard husbandry conditions were maintained with a stable temperature of 28 ± SD 0.5°C, pH 7.5, water conductivity of 1500 μS/cm and photoperiod of 12 h light/12 h dark. Feeding was performed twice a day with a total daily feed amount equal to 2.5% (w/w) of the body weight. Feeding behavior and health were regularly observed. The fish were fasted for 24 h prior to sampling. During the sampling, the fish in each tank were separated by gender and weighed. After euthanasia with a lethal dose of 200 mg/L tricaine methanesulfonate (MS222) (Sigma-Aldrich, St. Louis, United States), buffered with an equal amount of sodium bicarbonate, the fish were immediately frozen in liquid nitrogen and stored at −80°C until use. The skin was removed from the frozen specimens and fast muscle for RNA-seq analysis was carefully dissected under the microscope. Great care was taken to obtain solely fast muscle but it is plausible that slow muscle, muscle progenitor cells, neurons, immune cells and connective tissue were included. Therefore, the samples comprise a mixed cell population that is fast-muscle. Six females were randomly selected, including at least one fish from each of the four tanks per treatment. Statistical analysis of differences in specific growth rate among the dietary groups was performed with one-way ANOVA using R^1^. Assumptions for statistical analysis, normality of the distribution and homogeneity of variances were assessed, respectively, using the Shapiro-Wilk test and Levene’s test. Pairwise comparisons were analyzed using Tukey’s honest significance test, and *p*-value after adjustment for the multiple comparisons <0.05 was considered as statistically significant.

#### Salmon Feeding Trials and Sampling

Feeding trials were conducted using one-year-old post-smolt Atlantic salmon (Salmobreed, Lønningdal, Norway) with a starting mean weight of 223 g for 63 days at Nofima Research Station, Sunndalsøra, Norway as detailed by Johny et al. (2019). Each feeding group was allocated to three randomly distributed tanks (1 m^3^ and *n* = 15) and fish were fed to satiation with automatic disk feeders. They were reared at an average temperature of 10.6 ± SD 0.6°C and a seawater flow of 20 L/min. The oxygen levels of the tank outlets were above 80%. At the end of the feeding trials, the fish were anesthetized with 60–80 mg/L MS222, weighed and then euthanized with a double dose (120–160 mg/L) of MS222. After removing the skin, fast muscle was carefully dissected from the dorsal region to obtain fast-muscle enriched samples, which were frozen in liquid nitrogen and stored at −80°C until RNA isolation for qPCR analysis. Nine fish were randomly selected, including three fish from each of the three tanks per treatment. Their sex was undetermined, since they were not sexually mature at the time of sampling. Specific growth rates among the dietary groups were analyzed using one-way ANOVA, followed by Duncan’s multiple range test using SAS software (as detailed in the related manuscript under preparation). All the pertinent assumptions, normality of the distribution and homogeneity of variances were checked before performing ANOVA.

### RNA Sequencing

Total RNA was extracted from zebrafish fast muscle samples using QIAzol lysis reagent (Qiagen, Hilden, Germany), following the manufacturer’s protocol. Tissues were homogenized twice at 5000 × g for 15 s with zirconium oxide beads (1.4 mm; Precellys, Montigny-le-Bretonneux, France) using MagNALyser (Roche, San Francisco, United States). RNA integrity was checked using an Agilent 2200 TapeStation (Agilent Technologies, Santa Clara, United States), and RNA quantity was determined with a Qubit fluorometer (Invitrogen, Thermo Fisher Scientific, Waltham, United States). Libraries for RNA-seq were prepared using the NEBNext ultra II directional RNA library preparation kit with a poly(A) mRNA magnetic isolation module (NEB #E7490) in accordance to the manufacturer’s protocol (New England BioLabs Inc., Ipswich, United Kingdom). Briefly, after poly(A) enrichment from 1 μg total RNA, mRNA was fragmented to about 100–200 nt lengths and used for 1st and 2nd strand cDNA synthesis. After A-tailing, end repair and adaptor legation, the second strand was selectively removed using uracil-specific excision reagent (USER^®^ II Enzyme; New England BioLabs Inc.). The resulting directional RNA-seq libraries were amplified with 8 PCR cycles and later purified using AMPure XP beads (Beckman Coulter, Inc., Brea, United States). Quality and quantity of the RNA-seq libraries were assessed using the Agilent 2200 TapeStation. Sequencing was performed on the Illumina NextSeq platform (Illumina, San Diego, United States) at Nord University using a single-end 75 bp high-throughput sequencing kit with 4% Phix control DNA (Illumina) as internal control.

### RNA-Seq Data Analysis

Raw sequencing data were processed for quality and adapter trimming using Cutadapt (Martin, 2011) with -q 25, 20, quality-base = 33, trim-n -m 20 parameters, followed by a further quality check with FastQC^2^. Quality trimmed reads were mapped to the zebrafish genome and transcriptome downloaded from Ensembl (^3^ release 91) with TopHat2, version 2.1.0 (Kim et al., 2013). Indexing of the genome prior to mapping was done with Bowtie 2 (Langmead and Salzberg, 2012) and Tophat2. HTSeq (Anders et al., 2014) was used to compute gene expression values. Differentially expressed genes in fish fed with plant protein-based aquafeeds in comparison to their control diet counterparts were determined using DESeq2 (Anders and Huber, 2010) with the criteria adjusted *p*-value (q) with the Benjamini-Hochberg procedure ≤0.05 and absolute fold-change ≥1.5.

### qPCR Analysis

We used paralogue-specific qPCR assays to determine the expression of Atlantic salmon putative orthologs of several differentially expressed genes in zebrafish fed with soy or wheat diets as compared to the fishmeal control diet (Table 1). The Atlantic salmon had been fed with customized diets containing equal percentages of the same plant proteins as the zebrafish. Total RNA was extracted from Atlantic salmon fast muscle (*n* = 9) using the above protocol, treated with DNase I (RNase-Free DNase Set from Qiagen) to digest any residual genomic DNA, and subjected to column-purification using RNeasy Mini Kit (Qiagen), following the manufacturer’s instructions. RNA integrity was confirmed by 1% (w/v) agarose gel electrophoresis, and purity was assessed using a NanoDrop UV spectrophotometry (NanoDrop, Wilmington, United States). First-strand cDNA templates for qPCR were synthesized in 20 μL reactions from 1 μg of DNaseI-treated, column-purified total RNA using random primers (250 ng; Invitrogen/Life Technologies), dNTPs (0.5 mM final concentration; Invitrogen/Life Technologies), and M-MLV reverse transcriptase (200 U; Invitrogen/Life Technologies) with the manufacturer’s first strand buffer (1× final concentration) and DTT (10 mM final concentration) at 37°C for 50 min.

**TABLE 1:** Atlantic salmon homologs related to zebrafish genes that are differentially expressed by feeding with plant-based diets.

Gene name (symbol)	Zebrafish		Atlantic salmon	
	NCBI accession number	Differential expression	Paralogues*	NCBI accession number
*myosin light chain, phosphorylatable fast skeletal muscle b* (*mylpfb*)	NM_001004668.1	Soy group 2.7-fold decrease	***mylpfba***	**XM_014203362.1**
			***mylpfbb***	**NM_001123716.1**
			*mylpfbc*	XM_014161797.1
*heat shock protein hsp90-alpha1* (*hsp90aa1.1*)	NM_131328.1	Wheat group 3.9-fold decrease	***hsp90aa1.1a***	**XM_014205881.1**
			***hsp90aa1.1b***	**XM_014144832.1**
*activating molecule in beclin-1-regulated autophagy* (*ambra1a*)	NM_001281992.1	Soy group 2.1-fold increase	***ambra1aa***	**XM_014175106.1**
			***ambra1ab***	**XM_014126406.1**
			***ambra1ac***	**XM_014147648.1**
			*ambra1ad*	XM_014123983.1
*collagen type II, alpha 1* (*col2a1a*)	NM_131292.1	Soy group 2.6-fold decrease	***col2a1aa***	**XM_014134054.1**
			***col2a1ab***	**XM_014168236.1**
			*col2a1ac*	XM_014135018.1
			*col2a1ad*	XM_014145553.1
*betacellulin* (*btc*)	NM_001044764.3	Wheat group 2.6-fold increase	***btca***	**XM_014129351.1**
			*btcb*	XM_014138426.1
*ryanodine receptor 1a* (skeletal) (*ryr1a*)	XM_021479405.1	Soy group 2.2-fold increase	***ryr1aa***	**XM_014196267.1**
			*ryr1ab*	XM_014129250.1
*ornithine decarboxylase* (*odc1*)	NM_131801.2	Soy group 2.6-fold decrease	***odc1a***	**XM_014211026.1**
			***odc1b***	**XM_014192087.1**

*Genomic location and pairwise alignment details of paralogues are presented in Supplementary Table 2. Paralogues used for qPCR analysis are shown in bold letters. Paralogues, *btcb* (XM_014138426.1) and *ryr1ab* (XM_014129250.1) were not used for qPCR analysis, since the primers tested failed to show amplification. Paralogues *mylpfbc* (XM_014161797.1), *ambra1ad* (XM_014123983.1), *col2a1ac* (XM_014135018.1), and *col2a1ad* (XM_014145553.1) had very low expression levels in all dietary groups and therefore were not included in the differential expression analysis.

Putative paralogous genes in Atlantic salmon corresponding to selected zebrafish genes were identified by BLASTn searches against the non-redundant nucleotide and expressed sequence tags (EST) databases at NCBI. Genomic location and pairwise alignment details of paralogues are presented in Supplementary Table 2. Additionally, an overview of their genomic neighborhood was retrieved from Ensembl (see text footnote 3) as shown in Supplementary File 1. All identified paralogues were aligned using ClustalW^4^, and paralogue-specific primers were designed to target regions where there were at least 2 bases different between paralogues to ensure specificity. Alignments of the paralogues with the primers highlighted are presented in the Supplementary File 2. Primers were designed either with primer-BLAST (NCBI) or manually and analyzed with Net Primer^5^ (Table 2).

**TABLE 2:** Primers employed for paralogue-specific qPCR study in Atlantic salmon fast muscle.

Gene	Accession number	Sequence 5′–3′ §	Efficiency (%)	Size (bp)
*mylpfba*	XM_014203362.1	F-CTGTTCTCTCATACCTTCCTTCTCTT	110.5	113
		R-CACCCGCTCTCACACAAATATC		
*mylpfbb*	NM_001123716.1	F-CTATTCTCTTTATGTGTACTCGTGTGC	81.3	96
		R-GCCCGCTCTCACACAAATGT		
*hsp90aa1.1a*	XM_014205881.1	F-GAAGTGTATAGAGCTTTTCACAGAACTC	87.7	178
		R-CAGGGAAACCATTTCATCACC		
*hsp90aa1.1b*	XM_014144832.1	F-AAAGTGTATGGATCTTTTCATCGAG	93.3	179
		R-TCAGGGAGACCATTTCGTCAG		
*ambra1aa*	XM_014175106.1	F-CGTAGCAGTGGGAAAAGCTAAC	86.1	78
		R-GCCATTGCATGGTGAAATCCA		
*ambra1ab*	XM_014126406.1	F-GGCAACATTGTCATCAGCTCC	103.5	103
		R-CAGAACCATGTTTGTAACTAACTAGCTA		
*ambra1ac*	XM_014147648.1	F-AACAACAGCAGCAGCAGAGATAG	107.7	136
		R-AGCATCGTCACTAGCATCAGTC		
*col2a1aa*	XM_014134054.1	F-CATGGTGCCTCAGCCCTATC	86.6	125
		R-CATTGGCTGCCTCTTTCTCC		
*col2a1ab*	XM_014168236.1	F-CAGCGGTCTGGGTCTGGC	100.0	112
		R-CAGTTGGCGGACGGAGG		
*btca*	XM_014129351.1	F-CAGGGTATCCTACTAAACCGAGGA	105.1	168
		R-TAGGGCCAAAGCTGTGGCTAT		
*ryr1aa*	XM_014196267.1	F-CTGCTGAACCTGCTCCTGCT	97.4	76
		R-TTTTCAGTGTCTGCCTTCTCGG		
*odc1a*	XM_014211026.1	F-CTTGTCCAATTCTTTTGTGACAATCT	90.5	78
		R-CAGGAAGACAAAATCAGGAGCA		
*odc1b*	XM_014192087.1	F-CCAGTTTTCTTTTGTGACCAATCTA	87.9	143
		R-TCATCCGACATGGACATCTCA		
igf1*	NM_001123623.1	F-CCTGTTCGCTAAATCTCACTTC	91.5	227
		R-TACAGCACATCGCACTCTTGA		
igfII*	NM_001123647.1	F-GAAAACACAAGAATGAAGGTCAA	124.1	127
		R-CCACCAGCTCTCCTCCACATA		
igfbp-1a2*	NM_001279137	F-CTAAACCCCCAAACCCAGAT	134.8	104
		R-GTTGTGGCCAGGAAGGTAGA		
*ef1a2¤*	BG933853	F-GCACAGTAACACCGAAACGA	88.0	132
		R-ATGCCTCCGCACTTGTAGAT		
*actb¤*	BG933897	F-CCAAAGCCAACAGGGAGAAG	99.5	91
		R-AGGGACAACACTGCCTGGAT		

^§^Alignments of the paralogues with the primers highlighted are presented in Supplementary File 2. *Genes were tested in addition to the selected genes that were differentially regulated in the zebrafish feeding experiment: *igfI-insulin-like growth factor I*, *igfII-insulin-like growth factor II*, *igfbp-1a2-insulin-like growth factor binding protein 1 paralog A2.* ¤Genes selected as reference genes for qPCR analysis.

Primer quality was assessed to ensure amplification of a single product without primer dimers by melt curve analysis using cDNA prepared with a pool of RNA with equal contribution from all the samples used in the qPCR study. The size of the amplicons corresponding to each primer pair was verified by 2% (w/v) agarose gel electrophoresis using 1 kb plus ladder (Invitrogen/Life Technologies). Amplification efficiencies (Pfaffl, 2001) of primer pairs were calculated using a 5-point 1:3 dilution series starting with the pooled cDNA representing 10 ng of input total RNA. qPCR was performed in triplicate using Power SYBR Green I dye chemistry in 384-well format on a ViiA 7 real time PCR system (Applied Biosystems, Foster City, CA, United States) for the normalizer and target genes. Each 13 μL reaction mixture contained 1× Power SYBR Green PCR Master Mix (Applied Biosystems), 50 nM of both the forward and reverse primers, and 4 μL diluted cDNA (corresponding to 5 ng of input total RNA). The thermocycling profile was as follows: 1 cycle at 50°C for 2 min and 1 cycle at 95°C for 10 min, followed by 40 cycles at 95°C for 15 s and at 60°C for 1 min. The fluorescence signal data were collected after each 60°C step. Minus reverse transcriptase and no-template reactions were used as negative controls to confirm that there were no genomic and reagent contaminations, respectively.

Reference genes for normalization were selected after analyzing six candidate reference genes, including *actb* (*β-actin*), two paralogues of *elongation factor 1 alpha* [*ef1a1* (Olsvik et al., 2005) and *ef1a2* (Xue et al., 2015)], *pabpc1* (*polyadenylate-binding protein 1*), *rpl32* (*60S ribosomal protein L32*) (Xue et al., 2015), and *eif3d* (*eukaryotic translation initiation factor 3 subunit D*) (Caballero-Solares et al., 2017). Evaluation of normalizer genes was carried out using all the replicate samples from each of the control and plant protein diet-fed groups using *geNorm* (Vandesompele et al., 2002). *actb* and *ef1a2* were selected as normalizer genes as they were found to be the most stable of the tested six reference genes (i.e., lowest M-value, a gene stability measure). The relative quantity (RQ) of each transcript was determined using a qBase relative quantification framework (Hellemans et al., 2007; Xue et al., 2019), with normalization to the expression levels of *actb* and *ef1a2*. The sample with the lowest normalized expression level was used as calibrator (i.e., assigned RQ 1.0) when determining the RQ of each gene.

Statistical analysis was performed using R (see text footnote 1). Changes in gene expression between the control and each of the plant-based diets were determined by either parametric unpaired *t*-test or the non-parametric Wilcoxon rank sum test with continuity correction, based on the fulfillment of all pertinent assumptions for statistical analysis. Normality of the distribution was analyzed using the Shapiro-Wilk test, and homogeneity of variances was assessed by *F*-test. Gene expression changes were considered statistically significant at *p* ≤ 0.05.

## Results

### Plant-Based Diets Induced Moderate Transcriptomic Changes in Zebrafish Muscle

**FIGURE 1:**
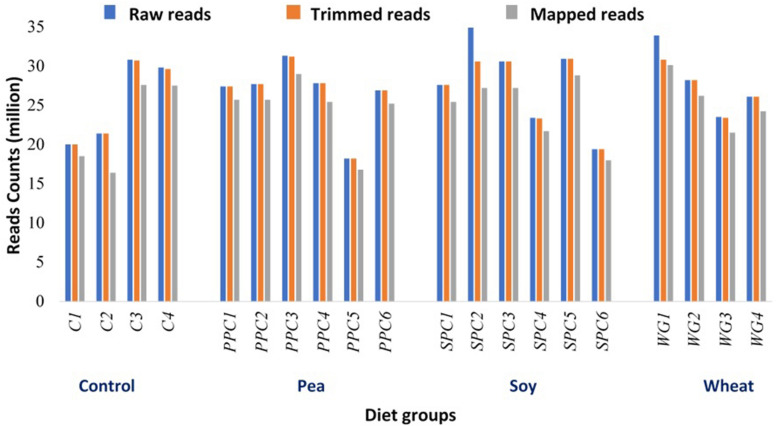
RNA-seq library characterization of individual replicates from zebrafish in control (C1–C4), pea (PPC1–PPC6), soy (SPC1–SPC6), and wheat (WG1–WG4) diet groups. Raw read counts were trimmed for quality and the adaptors were removed. The number of reads that could be aligned to the zebrafish genome and transcriptome (Ensembl release 91) is presented.

We used RNA-seq to determine the global transcriptomic changes in the fast-muscle enriched samples of zebrafish in response to plant protein-based diets that included 30% PPC (pea diet), SPC (soy diet), or WG (wheat diet), as well as a control diet containing fishmeal as the main protein source (Supplementary Table 1). The number of raw sequences ranged from 18 to 35 million per library. We obtained 18 to 31 and 16 to 30 million trimmed and mapped reads, respectively, per library (Figure 1). Each library had less than 7% of multiple mapping.

**FIGURE 2:**
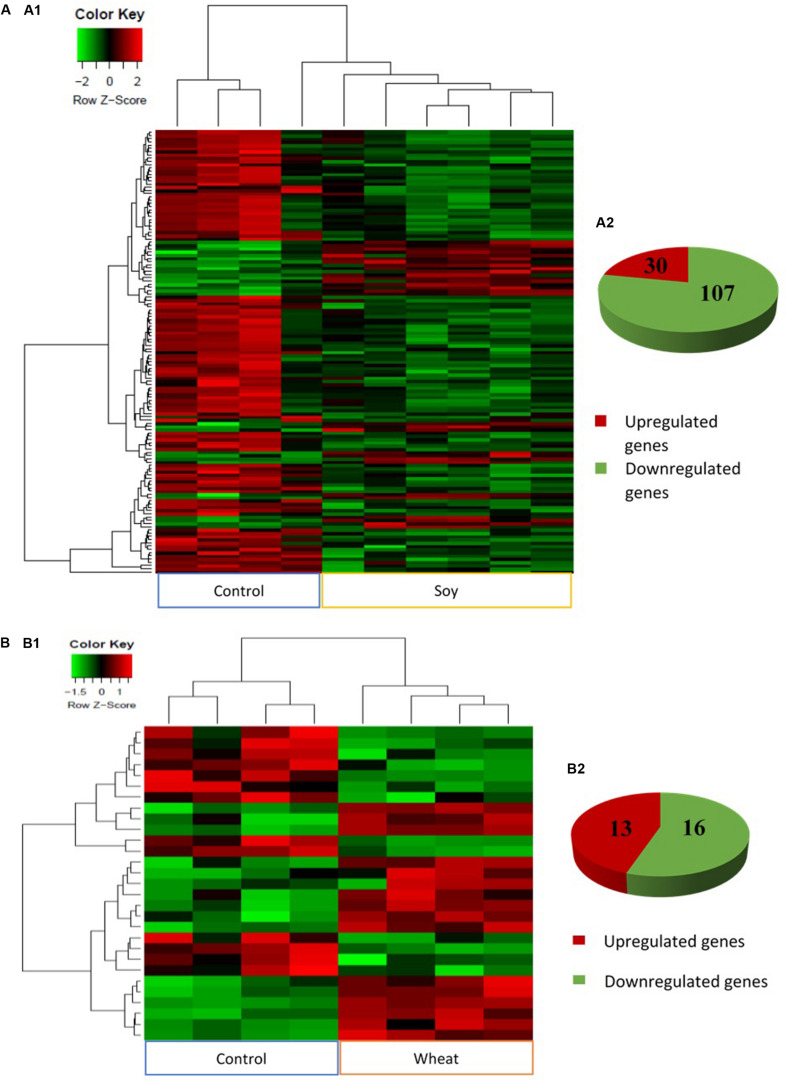
Heat maps generated by complete linkage method with Euclidean distance measure **(A1,B1)** and pie charts **(A2,B2)** showing significantly differentially expressed genes (absolute fold change ≥1.5, *q* < 0.05) in the fast muscle of zebrafish fed with the soy **(A)** and wheat **(B)** diets in comparison to controls fed with a diet containing fishmeal as the main protein source. Gradient scale indicates *Z*-scores of DEGs where red signifies most induced expression and green represents most reduced expression.

The analysis of global transcriptomic changes in the fast muscle of fish fed with the plant-based diets in comparison to the control group revealed 137 and 29 significantly differentially expressed genes (DEGs, fold change ≥ 1.5, Benjamini-Hochberg adjusted *p*-value < 0.05) in soy and wheat diet groups, respectively, with a higher proportion of downregulated genes (Figures 2A,B). However, the pea diet group did not show any significant changes compared to the control group within the applied statistical criteria.

### Feeding Soy and Wheat Diets Resulted in Regulation of Genes Involved in Growth, Structure and Function in Zebrafish Muscle

GO enrichment analysis of DEGs in the soy or wheat diet groups failed to show any enriched biological processes, most likely due to the relatively low number of DEGs. However, functional annotation revealed that several DEGs are involved in regulating fast muscle growth and its structure and function in zebrafish. The list of annotated DEGs in the soy and wheat diet groups compared to the control group and associated GO terms is presented in Supplementary Tables 3, 4, respectively. An important gene for fast and slow skeletal muscle development, *myosin light chain, phosphorylatable fast skeletal muscle b* (*mylpfb*, 2.7-fold), and a component of the myosin complex, *myosin heavy polypeptide 1.1* (*myhz1.1*, 2.7-fold), were downregulated in zebrafish fed with the soy diet, but not with the wheat diet. Whereas *popeye domain-containing* (*popdc3*), needed for skeletal muscle development, was upregulated in the same group of fish. Another gene involved in muscle development, *heat shock protein hsp90-alpha1* (*hsp90aa1.1*, 3.9-fold), was downregulated in zebrafish fed with the wheat diet, but its expression did not significantly change in the zebrafish fed with the soy diet. Two of the upregulated genes in zebrafish fed with the soy diet were *activating molecule in beclin-1-regulated autophagy* (*ambra1a*, 2.1-fold), a positive regulator of autophagy during skeletal muscle development, and *ryanodine receptor 1a* (*ryr1a*, 2.2-fold), an essential component of all skeletal muscle fiber calcium-release channels. On the other hand, a gene coding for a vital component of the skeletal muscle extracellular matrix, *collagen type II, alpha 1* (*col2a1a*, 2.6-fold), was downregulated in the same group of fish. Zebrafish fed with soy diet also showed downregulation of *ornithine decarboxylase* (*odc1*, 2.6-fold), which is important for polyamine biosythetic processes and myoblast proliferation. *betacellulin* (*btc*, 2.6-fold), a member of the epidermal growth factor family that mediates diverse processes including proliferation and differentiation, was upregulated in fast muscle of wheat diet-fed zebrafish.

Furthermore, several genes involved in metabolic processes including proteolysis [i.e., *dipeptidyl-peptidase 6b, dpp6b* (3.3-fold increase)], cholesterol biosynthetic process [i.e., apolipoproteins, *apoa1a, apoa1b* and *apobb.1* (2. 7-, 2. 8-, 2.9-fold decrease, respectively)] and glucose metabolism [i.e., *glyceraldehyde-3-phosphate dehydrogenase, gapdh* (1.6-fold decrease)] were differentially regulated in soy diet-fed zebrafish.

Among the genes differentially regulated in both soy and wheat diets-fed fish were *PDZ and LIM domain 1 (pdlim1*, downregulation), involving in actin cytoskeleton organization; serotransferrin (*tfa*, downregulation), important for ion transport as well as response to bacterium; *mutS homolog 3* (*msh3*, upregulation), vital for DNA repair; and *activating transcription factor 3* (*atf3*, downregulation), needed for regulation of transcription and fish immune responses (Feng and Rise, 2011).

A considerable number of *crystallin* genes (*crystallin, gamma* and *crystallin, beta*), mainly known to be important for visual perception, were downregulated in the soy diet fed group. Even though chaperone-like activity was described for mammalian *crystallin, gamma* (Andley et al., 1996), to our knowledge, the importance of this gene for fast muscle growth and homeostasis in mammals or fish has not been reported.

### Differential Expression of Atlantic Salmon Homologs Related to Zebrafish Genes That Are Differentially Expressed With Feeding Plant-Based Diets

The expression of selected genes that were differentially regulated with the diet in zebrafish fast muscle was subsequently examined in the fast-muscle enriched samples of Atlantic salmon fed with equivalent diets. Considering the salmonid genome duplication event, we used paralogue-specific qPCR assays to quantify the transcript levels of Atlantic salmon homologs of the selected zebrafish genes (Table 1).

**FIGURE 3:**
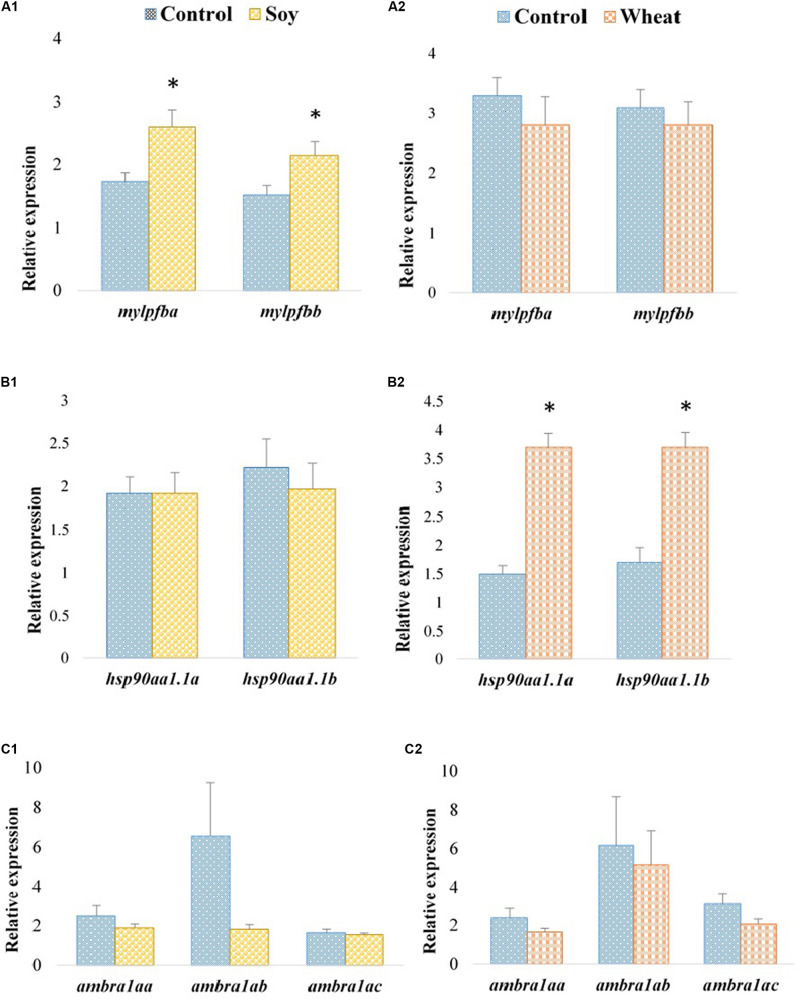
Expression profiles of *mylpfb, hsp90aa1.1*, and *ambra1a* paralogues in the fast muscle of Atlantic salmon fed with the soy **(A1,B1,C1)** and wheat **(A2,B2,C2)** diets compared to controls fed with a diet containing fishmeal as the main protein source. Values are means ± S.E.M (*n* = 7–9). Asterisks above the bars indicate significant changes (*p* < 0.05) in transcript levels.

**FIGURE 4:**
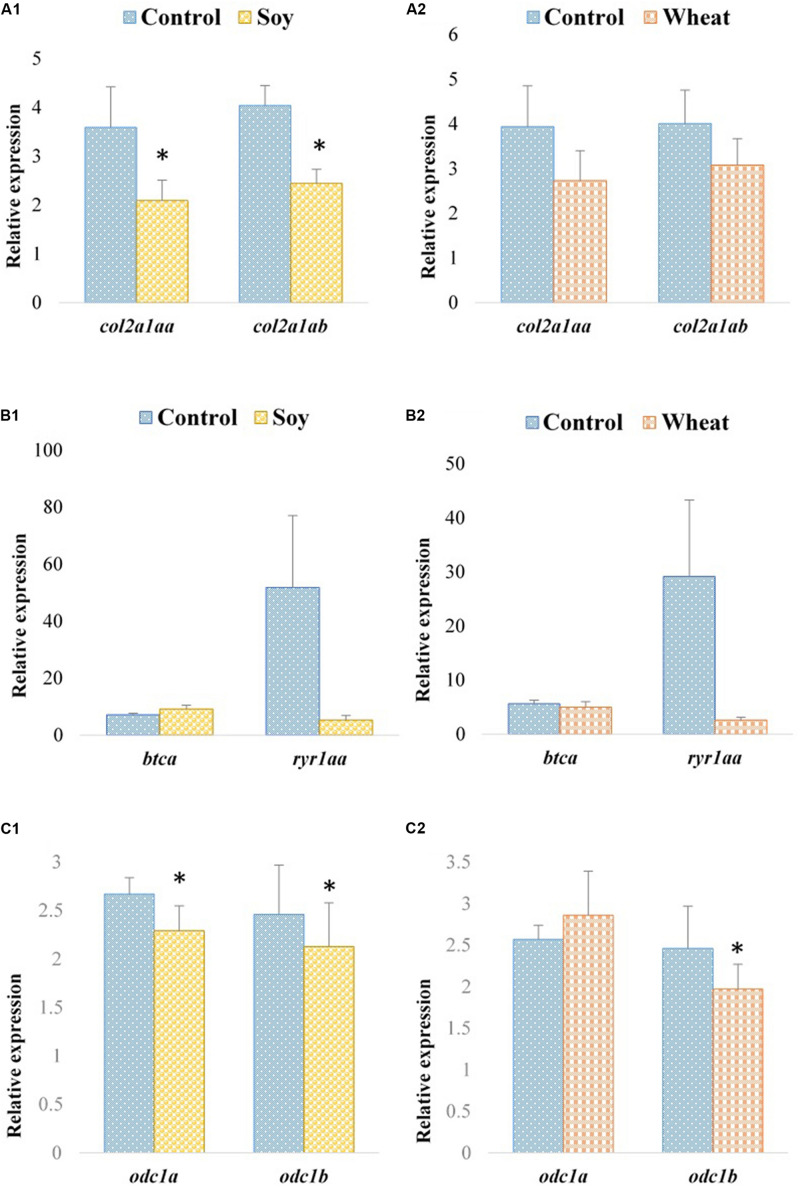
Expression profiles of *col2a1a*, *btc*, *ryr1a*, and *odc1* paralogues in the fast muscle of Atlantic salmon fed with the soy **(A1,B1,C1)** and wheat **(A2,B2,C2)** diets compared to controls fed with a diet containing fishmeal as the main protein source. Values are means ± S.E.M (*n* = 7–9). Asterisks above the bars indicate significant changes (*p* < 0.05) in transcript levels.

Expression profiles of the selected paralogous genes in fast muscle of Atlantic salmon are presented in Figures 3, 4. The paralogues *mylpfba* and *mylpfbb* were upregulated in the soy diet group (Figure 3A1) whereas the zebrafish putative ortholog was downregulated in those fed with the same plant-based diet. There was no significant regulation of *mylpfb* observed for wheat diet fed zebrafish or Atlantic salmon. Both the *hsp90aa1.1a* and *hsp90aa1.1b* paralogues (Figure 3B2) were upregulated (2.5- and 2.2-fold, respectively) in wheat diet-fed Atlantic salmon, contrary to the downregulation observed in wheat diet-fed zebrafish. The expression levels of all three *ambra1a* paralogues did not change significantly in soy diet-fed Atlantic salmon (Figure 3C1) even though the putative ortholog was upregulated in the zebrafish counterparts. They were also not significantly changed in Atlantic salmon fed with the wheat diet (Figure 3C2). Similarly, to the findings in zebrafish, *col2a1aa* and *col2a1ab* were downregulated (1.7-fold each) in Atlantic salmon fed with the soy diet (Figure 4A1). The expression of *btca* and *ryr1aa* paralogues did not change significantly in soy or wheat diet-fed Atlantic salmon (Figures 4B1,2). Both *odc1* paralogues were downregulated (1.2-fold each) in soy diet-fed Atlantic salmon (Figure 4C1), comparable to their zebrafish counterparts. Further, *odc1* paralogues were also downregulated in Atlantic salmon fed with wheat diet (Figure 4C2) while they were not significantly changed in the zebrafish fed with the similar plant-based diet.

**FIGURE 5:**
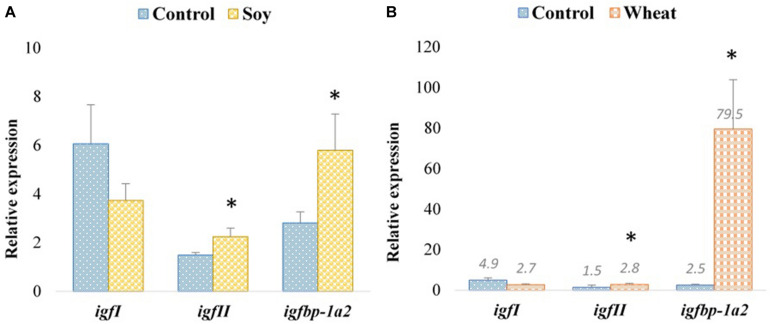
Expression profiles of IGF-signaling genes in the fast muscle of Atlantic salmon fed with the soy **(A)** and wheat **(B)** diets compared to controls fed with a diet containing fishmeal as the main protein source. Values are means ± S.E.M (*n* = 9). Asterisks above the bars indicate genes, whose transcript levels changed significantly (*p* < 0.05) with the plant-based diet.

We also analyzed the expression of genes involved in insulin-like growth factor (IGF) signaling in the fast muscle of Atlantic salmon fed with soy and wheat diets. The transcript expression of *insulin-like growth factor I* (*igfI*) did not show any significant changes with respect to the diet (Figures 5A,B). In contrast, *insulin-like growth factor II* (*igfII*, 1.5- and 1.9-fold, respectively, for soy and wheat diet groups) and *insulin-like growth factor binding protein 1 paralog A2* (*ifbp-1a2*, 2.1- and 31.8-fold, respectively, for soy and wheat diet groups) were upregulated with both soy and wheat diet groups (Figures 5A,B).

### Growth Performance of Zebrafish and Atlantic Salmon During the Feeding Studies

**FIGURE 6:**
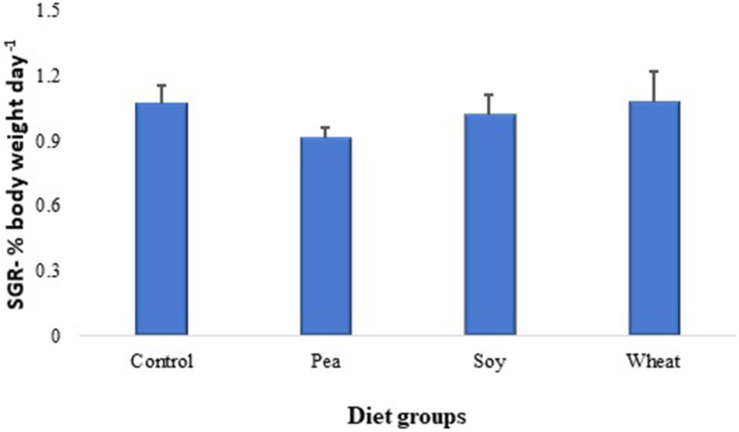
Specific growth rates of zebrafish fed for 46 days with diets containing 30% plant proteins from pea, soy and wheat or with a fishmeal-based control diet. Values are means ± S.E.M (*n* = 32 females).

The specific growth rate of zebrafish fed with diets including 30% PPC, SPC, or WG did not significantly change after 46 days of the feeding trial as compared to their counterparts fed with a diet containing fishmeal as the main protein source (Figure 6). Moreover, inclusion of 30% SPC also did not show any significant changes in the specific growth rate of Atlantic salmon compared to counterparts fed with a diet containing fishmeal as the main protein source; on the other hand, inclusion of 30% WG had a significant reduction in specific growth rate in salmon compared to the control group (growth data are included in the related manuscript under preparation).

## Discussion

The present study reports the nutrigenomic effects of fish diets containing partial inclusion of plant-based proteins, e.g., pea, soy, and wheat, in the fast muscle of zebrafish. Global gene expression changes in the muscle tissues of zebrafish fed with plant-based diet were moderate yet included several genes important for the regulation of muscle growth, maintenance, function and homeostasis.

The specific growth rate of zebrafish was not significantly affected by any of the plant protein-based diets used in the present study after a 46-day feeding trial. Inclusion of plant-based proteins in aquafeeds can affect fish growth, depending on the percentage of replacement and species. For instance, 50% replacement of fishmeal with SPC did not affect the growth rate in Atlantic salmon (Storebakken et al., 2000b), whereas 25% replacement of fishmeal with SPC significantly reduced the growth rate of Japanese flounder (Deng et al., 2006). The total replacement of marine protein sources with plant-based proteins from 35 to 98 days post-fertilization reportedly decreased the specific growth rate of zebrafish (Ulloa et al., 2013). The plant-based diets used in our study (Johny et al., 2019) contained still a considerable amount of fishmeal (49.4%) in addition to the 30% plant protein, resulting in a crude protein content of 50 g pea, 55 g soy, and 59 g wheat per 100 g feed, which was similar to the control diet with 56 g crude protein/100 g feed. The relatively high ratio of fishmeal in the plant-based diets has probably helped the zebrafish to maintain a similar growth rate as their control counterparts fed with 79.4% fishmeal. Moreover, we found that Atlantic salmon fed with plant-based diets containing 33.4% fishmeal in addition to 30% SPC also did not show any significant changes in the specific growth rate; however, salmon fed with the diet containing 30% WG had a growth rate decreased by 11.1% compared to the controls that were fed with 63.4% fishmeal.

The analysis of DEGs in the muscle of zebrafish fed with the plant protein-containing diets showed that the DEG numbers varied with the plant ingredient. They were highest in fish fed with the soy diet followed by the fish fed with the wheat diet. Surprisingly, the pea diet did not induce any significant gene expression changes as compared to the fishmeal control group. Among the DEGs changed in the soy and wheat diet-fed groups, there were genes associated with muscle growth, function, metabolism and homeostasis, some of which were also differentially expressed in Atlantic salmon fed with the same plant-based protein diets, while others showed species specific regulation. Even though those gene expression changes have not significantly influenced their growth, we believe that these molecular adjustments occurred in the muscle to maintain muscle homeostasis following imbalances caused by dietary plant ingredients. Moreover, our recently published study (Dhanasiri et al., 2020) from the same feeding trial also indicated plant-based diets induced epigenetic changes in the intestine with mild inflammation without significantly affecting their growth.

All teleosts share three rounds of whole-genome duplication (WGD), 1R, 2R, and 3R where the last was a teleost-specific WGD (Glasauer and Neuhauss, 2014; Lien et al., 2016). Additionally, a fourth (4R) WGD occurred in the common ancestor of salmonids (Glasauer and Neuhauss, 2014). WGDs are important for evolutionary adaptations and innovations; complexity and diversifications. The duplicated genes after WGDs can undergo different fates including: non-functionalization (deleterious mutations occur in one of the duplicates leading to loss of expression); subfunctionalization (mutations in paralogous genes leading to preservation of both duplicates); neofunctionalization (one of the genes acquires a novel function) (Glasauer and Neuhauss, 2014). Differential expression patterns of Atlantic salmon homologs (putative orthologs and paralogues) related to zebrafish genes regulated by dietary inclusion of plant-based diets observed in our study detailed in the following sections, provide possible evidence for gene duplication and divergence. However, functional studies are needed to clarify the fate of the Atlantic salmon paralogous genes to determine whether they were subfunctionalized or neofunctionalized.

Skeletal muscle is a highly adaptable tissue, which changes in size and cell composition in response to environmental and physiological conditions. Its homeostasis can be challenged by both short and long term nutritional changes (Matsakas and Patel, 2009). Skeletal muscle fibers consist of myofibrils that are made up of sarcomeres containing organized arrays of actin thin filaments and myosin thick filaments along with accessory proteins (Johnston et al., 2011). Several muscle subtypes are described based on the expression of numerous isoforms of myosin light and heavy chains. Myosin heavy chain is mainly responsible for the functional and phenotypic diversity of muscles (Pette and Staron, 2000). The regulatory light chain of myosin in fast skeletal muscle is encoded by the *mylpf* gene in mammals (Wang et al., 2007), and ortholog, *mylpfb*, exists in zebrafish. In mouse, *mylpf* knockdown resulted in the complete lack of skeletal muscle during development, indicating the importance of *mylpf* for the growth of both fast and slow skeletal muscle (Wang et al., 2007). *mylpfb* was downregulated 2.7-fold in muscle of zebrafish fed with the soy diet and there were no other differentially expressed myosin-related genes that could compensate for this change but its regulation did not have a significant influence on growth. On the other hand, its homologs *mylpfba and mylpfbb* were upregulated 1.5- and 1.4-fold in Atlantic salmon fed with the same diet and these gene expression changes also did not affect growth significantly. These puzzling species-specific observations reflect the complexity of myogenesis and may be due to diet-related changes at regulation levels that were not addressed in the present study, such as post-translational modifications or expression of non-coding RNAs.

Several studies in zebrafish and Atlantic salmon demonstrated that heat-shock proteins 90α (Hsp90α), especially Hsp90α1, play an important role in myosin folding, myofibril assembly and myosin thick filament organization (Du et al., 2008; Garcia de la serrana and Johnston, 2013; He et al., 2015). Loss of Hsp90α1 function resulted in increased myosin protein degradation (Du et al., 2008) and disruption of all sarcomeric structures, including both thick and thin filaments in skeletal muscles in zebrafish (Codina et al., 2010). We observed a downregulation of *hsp90aa1.1* in zebrafish fed with the wheat diet, however, we did not observe a significant change in the specific growth rate of these fish. On the other hand, both the *hsp90aa1.1a* and *hsp90aa1.1b* paralogues were upregulated in the same group of salmon, concomitantly with a significant reduction in growth. Increased expression of those paralogues was also previously reported under the rapid enhancement of nutritional status, such as the initiation of feeding after starvation in juvenile salmon and supplementation of complete amino acids into the starved primary myogenic cultures (Garcia de la serrana and Johnston, 2013). Wheat diet-fed fish showed a significant reduction in specific growth rate indicating a possible lower nutritional status compared to the control group. Further studies are required to understand the regulation of *hsp90aa1* paralogues in Atlantic salmon under different nutritional conditions as well as their species-specific regulation.

In addition to genes coding for fast muscle components or directly involved in muscle growth and function, some genes indirectly involved in muscle functions, such as *col2a1a*, were downregulated in zebrafish and salmon fed with the soy diet. The skeletal muscle extracellular matrix (ECM) is vital for muscle fiber force transmission, maintenance and repair (Gillies and Lieber, 2011). Collagen is a major structural protein in muscle ECM even though it is a minor constituent (1–10%) of the muscle mass dry weight. *collagen type II* (*col2a1*) is expressed during skeletal muscle development in mammals (Gillies and Lieber, 2011) and zebrafish embryogenesis (Dale and Topczewski, 2011). Therefore, the downregulation of *col2a1a* caused by the soy diet in adult zebrafish and Atlantic salmon warrants further investigation. Moreover, both zebrafish and Atlantic salmon fed with the soy diet showed downregulation of *odc1*, a gene coding for ornithine decarboxylase, which is important for polyamine biosynthetic processes. Studies in mouse myoblasts and human skeletal muscle cell lines showed that *odc1* gene promotes myoblast proliferation (Lee et al., 2011). The downregulation of *odc1* in both zebrafish and salmon fed with the soy diet could be linked to high polyamine levels present in SPC, since ornithine decarboxylase levels are modulated by the available polyamine concentration (Pegg, 2006). This could have ultimately delayed myoblast proliferation and affected myogenesis.

Muscle growth is regulated by growth hormones acting directly through sarcolemmal receptors and indirectly *via* the insulin-like growth factor (IGF) pathway. The IGF system comprises IGF-I, IGF-II, several receptors and six binding proteins (IGFBPs) (Johnston et al., 2011). IGFs stimulate muscle growth in fish by promoting the proliferation of myogenic cells, protein synthesis and hypertrophy, as well as inhibiting protein degradation and muscle atrophy (Fuentes et al., 2013). The availability of IGFs is regulated by IGFBPs, and generally IGFBP-1 and -2 inhibit the growth-promoting functions of IGFs in both mammals and fish (Fuentes et al., 2013). Zebrafish selected for large body size showed higher *igf1* and lower *igfbp1a* mRNA levels compared to those selected for small body size in a study investigating, responses to changes in the nutritional input (Amaral and Johnston, 2012). Interestingly, a study in Atlantic salmon showed that switching to fast growth involves the local upregulation of *igfI*, *igfbp-5.2* and *igfbp-4* accompanied by downregulation of *igfbp-2.1* and *igfII* in the skeletal muscle (Bower et al., 2008). We did not observe differential expression of genes from the GH-IGF signaling system in zebrafish fed with any of the three-plant protein-based diets as compared to the fishmeal diet, which is in agreement with the lack of growth rate differences between the feeding groups. On the other hand, *igfII* and *igfbp-1a2* were upregulated in Atlantic salmon fed with the soy and wheat diets with normal and reduced growth, respectively, compared to the control group. Further studies are needed to explain the involvement of specific components of the GH-IGF system in regulating the growth changes observed in Atlantic salmon fed with different plant-based diets.

*Odc1* is the only salmon gene in our study whose paralogues show different expression patterns with the wheat diet, which suggests that they have undergone subfunctionalization following duplication. The identified paralogues of *mylpfb*, *hsp90aa1.1*, *ambra1*, and *col2a1* showed similar patterns of regulation with soy- and wheat diets, indicating functional redundancy. Nevertheless, cellular localization and functional studies are required to ascertain conclusively if these paralogues are indeed functionally redundant or undergoing subfunctionalization or neofunctionalization.

## Conclusion

The partial inclusion of proteins from soy and wheat into zebrafish feed resulted in the regulation of, respectively, 137 and 29 genes in muscle. In contrast, the inclusion of pea protein concentrate did not induce changes in the gene expression as compared to a control group receiving a fishmeal-based diet. Among the differentially expressed genes, several were important for muscle growth, structure, function and homeostasis. Some of these genes and their paralogues were similarly regulated in Atlantic salmon fed with equivalent diets while others showed species- specific regulation. Our results revealed that molecular adjustments occurred in the muscle of zebrafish and Atlantic salmon, probably to maintain muscle homeostasis following imbalances caused by dietary plant ingredients, even if there were no significant differences observed in the growth. Ultimately, this knowledge may be applied for the improved formulation of sustainable plant-based diets for the aquaculture industry.

## Data Availability Statement

The datasets presented in this study can be found in online repositories. The names of the repository/repositories and accession number(s) can be found below: https://www.ncbi.nlm.nih.gov/, PRJNA577226; https://www.ncbi.nlm.nih.gov/, SUB6971208.

## Ethics Statement

The animal study was reviewed and approved by Norwegian Animal Research Authority, Norway; Nord University, Norway.

## Author Contributions

AD, JF, MR, and CF conceived and designed the study. CF, JF, and MR contributed reagents and materials for the experiments. AD, XX, and AJ performed laboratory work. AD and GB conducted the zebrafish and Atlantic salmon feeding trials, respectively. AB prepared the feeds. AD, JF, and XX analyzed the data. AD, JF, XX, MR, and CF wrote the manuscript. All authors read and approved the manuscript.

## Conflict of Interest

The authors declare that the research was conducted in the absence of any commercial or financial relationships that could be construed as a potential conflict of interest.

## Footnotes

1 https://www.r-project.org/

2 https://www.bioinformatics.babraham.ac.uk/projects/fastqc/

3 http://www.ensembl.org

4 https://www.ebi.ac.uk/Tools/msa/clustalw2/

5 http://www.premierbiosoft.com/netprimer/
