# Supplementary material for: Plant-Based Diets Induce Transcriptomic Changes in Muscle of Zebrafish and Atlantic Salmon
Source: Front Genet. 2020 Oct 22;11:575237. doi: 10.3389/fgene.2020.575237 (PMC7642599; doi:10.3389/fgene.2020.575237)
Supplement: Supplementary file 1 [file Data_Sheet_1.docx]

**Supplementary File 1**

**Plant-based diets induce transcriptomic changes in fast muscle of**

**zebrafish and Atlantic salmon**

Anusha K.S. Dhanasiri ^1,2^*, Amritha Johny ^3^, Xi Xue ^4^, Gerd M Berge ^5^, Andre S. Bogevik^6^, Matthew L. Rise ^4^, Christiane K. Fæste ^3^ and Jorge M.O. Fernandes ^1^*

*^1^ Faculty of Biosciences and Aquaculture, Nord University, Bodø, Norway*

*^2^ Department of Paraclinical Sciences, Faculty of Veterinary Medicine, Norwegian University of Life Sciences (NMBU), Oslo, Norway*

*^3^ Toxinology Research Group, Norwegian Veterinary Institute, Oslo, Norway*

*^4^ Department of Ocean Sciences, Memorial University of Newfoundland, St. John's, NL A1C 5S7, Canada*

*^5^ Norwegian Institute of Food, Fisheries and Aquaculture Research (Nofima), Sunndalsøra, Norway*

*^6^ Norwegian Institute of Food, Fisheries and Aquaculture Research (Nofima), Fyllingsdalen, Norway*

**Corresponding authors**

Anusha K.S. Dhanasiri

Email: anusha.dhanasiri@nmbu.no

Jorge M.O. Fernandes

Email: jorge.m.fernandes@nord.no

**Genomic region overview of the respective paralogues downloaded from Ensembl database (**[**http://www.ensembl.org**](http://www.ensembl.org)**).**

***mylpfba* XM_014203362.1**

***mylpfbb-* NM_001123716.1**

***mylpfbc* - XM_014161797.1**

***hsp90aa1.1a*- XM_014205881.1**

***hsp90aa1.1b* - XM_014144832.1**

***ambra1aa* - XM_014175106.1**

***ambra1ab* - XM_014126406.1**

***ambra1ac* - XM_014147648.1**

***ambra1ad* - XM_014123983.1**

***col2a1aa* - XM_014134054.1**

No ensembl records

***col2a1ab* -XM_014168236.1**

No ensembl records

***col2a1ac* - XM_014135018.1**

***col2a1ad* - XM_014145553.1**

***btca* - XM_014129351.1**

***btcb* - XM_014138426.1**

***ryr1aa* - XM_014196267.1**

***ryr1ab* - XM_014129250.1**

***odc1a* - XM_014211026.1**

***odc1b* - XM_014192087.1**
