# Supplementary material for: Plant-Based Diets Induce Transcriptomic Changes in Muscle of Zebrafish and Atlantic Salmon
Source: Front Genet. 2020 Oct 22;11:575237. doi: 10.3389/fgene.2020.575237 (PMC7642599; doi:10.3389/fgene.2020.575237)
Supplement: Supplementary file 2 [file Data_Sheet_2.PDF]

Supplementary File 2

Plant-based diets induce transcriptomic changes in fast muscle of zebrafish and Atlantic salmon

Anusha K.S. Dhanasiri [1,2]*, Amritha Johny [3], Xi Xue [4], Gerd M Berge [5], Andre S. Bogevik[6], Matthew L. Rise [4], Christiane K. Fæste [3] and Jorge M.O. Fernandes [1]*

[1] Faculty of Biosciences and Aquaculture, Nord University, Bodø, Norway

[2] Department of Paraclinical Sciences, Faculty of Veterinary Medicine, Norwegian University of Life Sciences (NMBU), Oslo, Norway

[3] Toxinology Research Group, Norwegian Veterinary Institute, Oslo, Norway

[4] Department of Ocean Sciences, Memorial University of Newfoundland, St. John's, NL A1C 5S7, Canada

[5] Norwegian Institute of Food, Fisheries and Aquaculture Research (Nofima), Sunndalsøra, Norway

[6] Norwegian Institute of Food, Fisheries and Aquaculture Research (Nofima), Fyllingsdalen, Norway

Corresponding authors

Anusha K.S. Dhanasiri

Email: anusha.dhanasiri@nmbu.no

Jorge M.O. Fernandes

Email: jorge.m.fernandes@nord.no

Multiple sequence alignments of putative paralogues from Atlantic salmon.
The paralogous used for the qPCR analysis and statistical interpretation as well as the primer pairs used for the amplification of each paralogues are highlighted.

CLUSTAL 2.1 Multiple Sequence Alignments for *mylpfb*

Sequence 1: XM_014203362.1 (*mylpfba*) 1543 bp
Sequence 2: NM_001123716.1 (*mylpfbb*) 823 bp
Sequence 3: XM_014161797.1 (*mylpfbc*) 696 bp

Sequences (1:2) Aligned. Score: 94.4107
Sequences (1:3) Aligned. Score: 50.7184
Sequences (2:3) Aligned. Score: 48.9943

```
CLUSTAL 2.1 multiple sequence alignment


XM_014203362.1      CTCTCTCTCCCTCTCTCCCTCTCTCTGCCTCTACAAATCCAAACTTGAGGCTGTGGCTAA
NM_001123716.1      ------------------------------------------------------------
XM_014161797.1      ------------------------------------------------------------


XM_014203362.1      AGTTAGCACGGTCTACAGAGGTATATAAGCCCCCGAGGATAGGGGTTTAATGCGGCTTCA
NM_001123716.1      ------------------------------------------------------------
XM_014161797.1      -------------------------AGGGCAGAAGATAACATGGGCCATACATATAAACC


XM_014203362.1      CATCAGTCTCTTCTCTTGTTGACC--ATCCAAACCTCCATACATACCGTCTCGAGATGGC
NM_001123716.1      ------------CTCTTGTTGACC--ATCCAAACCTCCATACATACCGTCTCGAGATGGC
XM_014161797.1      CTCCCTACTAATCACACATAGTCCTGGACCAAGAGACATTGCACCACACACTGACATGGC
                                * *   * * **    ****    *  * **   *     ** *****

XM_014203362.1      ACCCAAGAAGGCC---AAGAGGAGGGGAGCAGCAGCAGAGGGCGGTTCCTCCAACGTGTT
NM_001123716.1      ACCCAAGAAGGCC---AAGAGGAGGGGAGCAGCAGCAGAGGGCGGTTCCTCCAACGTGTT
XM_014161797.1      CAGTAAGAAGGCTGCTAATAAGAGACAGAGGGGAGCCCAGAAGAGCTGCTCCAATGTCTT
                        ********    ** * ***       * ***  **    * * ****** ** **

XM_014203362.1      CTCCATGTTTGAGCAGAGCCAGATCCAGGAGTACAAGGAGGCTTTCACAATTATTGACCA
NM_001123716.1      CTCCATGTTTGAGCAGAGCCAGATCCAGGAGTACAAGGAGGCTTTCACAATCATTGACCA
XM_014161797.1      CTCCATGTTCGAACAGTCCCAGATACAGGAGTTCAAGGAGGCTTTTGGCTGTATTGACCA
                    ********* ** ***  ****** ******* ************       ********

XM_014203362.1      GAACAGAGACGGTATCATCAGCAAGGATGACTTGAGGGACGTGCTGGCCTCAATGGGCCA
NM_001123716.1      GAACAGAGACGGTATCATCAGCAAGGATGACTTGAGGGACGTGCTGGCCTCAATGGGCCA
XM_014161797.1      AGACAGAGATGGTGTCATCAAAAAGCAGGATCTGAGGGAAACCTATGGACAGCTAGGAAA
                      ******* *** ******  *** * **  *******       *      * **  *

XM_014203362.1      GTTGAATGTGAAGAATGAGGAGCTGGAAGCCATGGTCAAGGAGGCCAGCGGCCCCATCAA
NM_001123716.1      GTTGAATGTGAAGAATGAGGAGCTGGAAGCCATGGTCAAGGAGGCCAGCGGCCCCATCAA
XM_014161797.1      GCTGAACGTAAAGGATGAGGAGCTGGATGAGATGTTGAACGAGGGGAAGGGTCCCATTAA
                    * **** ** *** ************* *  *** * ** ****  *  ** ***** **

XM_014203362.1      CTTCACCGTCTTCCTCACCATGTTTGGAGAGAAGCTCAAGGGTGCTGATCCCGAGGATGT
NM_001123716.1      CTTCACCGTCTTCCTCACCATGTTTGGAGAGAAGCTCAAGGGTGCTGATCCCGAGGATGT
XM_014161797.1      CTTCACCGTTTTCCTCTCTTTGTTTGGGGAGAAGCTCAATGGCACAGACCCTGAGGACAC
                    ********* ****** *  ******* *********** **  * ** ** *****

XM_014203362.1      CATCGTTAGTGCTTTCAAGGTCCTGGACCCCGAGGCTACCGGTTTCATCAAGAAGGAATT
NM_001123716.1      CATCGTTAGTGCTTTCAAGGTCCTGGACCCCGAGGCTACCGGTTTCATCAAGAAGGAATT
XM_014161797.1      CATCCTCGCTGCCTTCAAGCTCTTTGACCCAAACGGCACAGGCTTCGTCAACAAGGATGA
                    **** *   *** ****** ** * *****  * *  ** ** *** **** *****

XM_014203362.1      CCTTCAGGAGCTCCTGACCACTCAGTGCGACAGGTTCTCTGCAGAGGAGATGAAGAACCT
NM_001123716.1      CCTTCAGGAGCTCCTGACCACTCAGTGCGACAGGTTCTCTGCAGAGGAGATGAAGAACCT
XM_014161797.1      GTTTAGACGATTACTGATGAACCAGGCTGATAAATTCACAGCAGATGAGGTGGATCAGGC
```

```
                                  **       * ****  *  ***   ** *  *** * ***** *** ** *  *

XM_014203362.1      GTGGGCTGCCTTCCCCCCAGATGTGGCCGGCAACGTAGACTACAAGCAAATCTGCTACGT
NM_001123716.1      GTGGGCTGCCTTCCCCCCAGATGTGGCCGGCAACGTAGACTACAAGCAAATCTGCTACGT
XM_014161797.1      CTTCTCTGTGGCTCCAATTGACGTGGCCGGCAACATCGACTACAAGTCACTGTGTTACAT
                     *   ***     **    ** ************ * *********  * * ** *** *

XM_014203362.1      CATCACACACGGAGAGGAGAAGGAGGAGTAATGAAACAGACATAAGAAAAGAAAACAGCC
NM_001123716.1      AATCACACACGGAGAGGAGAAGGAGGAGTAATGAAACAGACAGAAGAAAAGAAAACAGCC
XM_014161797.1      CATCACACACGGAGACGAGAAGGAGGAGGCCTAAAGACGTTCTGACACCTGAAAG-GGCA
                     ************** ************   * **   *     * *   ****   **

XM_014203362.1      TCCCTTGCCATTCTACCTTCCTGCTCCTCTCTTCTTCCTTCTGTTCTCTCATACCTTCCT
NM_001123716.1      TCCCTTGCCATTCTACCTTCCTGCTCCTCTGTTCTTCCTTCTATTCTCT-----------
XM_014161797.1      GAACTGG--AATCT-CCTCTGTGCCCGTCTTTTGCCAATACACTAAAGACA---------
                       ** *  * *** ***   *** * *** **     * *  *

XM_014203362.1      TCTCTTTATGTGTACTCATGTGCTCTGTCTCTCACTCACACAAATTCTCTAAAAGACTCG
NM_001123716.1      -----TTATGTGTACTCGTGTGCTCTGTCTCTCACGCACACAAATTCTCTAAAAGACTCG
XM_014161797.1      -----TTTTCAAAAATCAA-----------------------------------------
                         ** *    * **

XM_014203362.1      TCTCTACTCAAGATATTTGTGTGAGAGCGGGTGCTCATGGGTTGTCTATGTTTGTTCGTG
NM_001123716.1      TCTCTACTCAAGACATTTGTGTGAGAGCGGGCGCTCATGGGTTGTCTATGTTTGTTCGTG
XM_014161797.1      ------------------------------------------------------------


XM_014203362.1      GGGATATGGGATTATTTTCAATAAAAATGATCTTTTAACATCATCTCTTTCTCACCCGCT
NM_001123716.1      GGGATATGGGATTATTTTCAATAAAAATGATCTTTTAACATCTCCAAAAAAAAAAAAAAA
XM_014161797.1      ------------------------------------------------------------


XM_014203362.1      TTCCCTTCCCTTAACCCTCTTCCTCCTTTTCATCTCTTACTCCTATCTATCATTGGTTGT
NM_001123716.1      AAAAAAAAAAAAAAAA--------------------------------------------
XM_014161797.1      ------------------------------------------------------------


XM_014203362.1      TTCCCCAGTCTCTCTGTTAGAAGAAATGCACACAGCAAGTCGTATTCCACTGCTGTGGTA
NM_001123716.1      ------------------------------------------------------------
XM_014161797.1      ------------------------------------------------------------


XM_014203362.1      TGGATGTGTAAACCCCAAGGTGAAATGGAGCACCAGTCTTTGCCTCGGCTGCAACAGCTC
NM_001123716.1      ------------------------------------------------------------
XM_014161797.1      ------------------------------------------------------------


XM_014203362.1      AGAGGTTAGACCTGGAAGTCCTTTCACTCCCCCTTAATTTAACCCTCAGATGGGCAAATA
NM_001123716.1      ------------------------------------------------------------
XM_014161797.1      ------------------------------------------------------------


XM_014203362.1      AACCAAACAGAGGGATATCGTTCTATAAATATTGTAGAGGTTATCTCCTGTAGATAAGGT
NM_001123716.1      ------------------------------------------------------------
XM_014161797.1      ------------------------------------------------------------


XM_014203362.1      GGGAAAATAGGAGATATTCTAAGAAGGAATGAGGCTAGAGAAAAACATGGCCTCCTTAAA
NM_001123716.1      ------------------------------------------------------------
XM_014161797.1      ------------------------------------------------------------


XM_014203362.1      GTGACGGCTACGTTTTTCTCAAGTGAGAAAGGCTGCTAGAGAGAAGAGAAAGGACCCAAA
NM_001123716.1      ------------------------------------------------------------
XM_014161797.1      ------------------------------------------------------------


XM_014203362.1      GAAAACACTCGTCTTCACCTCCTTTCCCTCCTCCCTTTCCTCCTTTACTTCTACCTGTTT
```

```
NM_001123716.1      ------------------------------------------------------------
XM_014161797.1      ------------------------------------------------------------

XM_014203362.1      TCTATAAGCTCCTTTGTTTTCTGCACCTGGACTAGCTTTAGTCTGCCAGAAACTCCTCTG
NM_001123716.1      ------------------------------------------------------------
XM_014161797.1      ------------------------------------------------------------

XM_014203362.1      TAACGAATAAAAGGGACTAACTGTGAATAAAAGCTCTTATCTTTTGTA
NM_001123716.1      ------------------------------------------------
XM_014161797.1      ------------------------------------------------
```

CLUSTAL 2.1 Multiple Sequence Alignments for *hsp90aa1.1*

Sequence 1: XM_014205881.1(*hsp90aa1.1a*) 2155 bp
Sequence 2: XM_014144832.1 (*hsp90aa1.1b*) 2155 bp

Sequences (1:2) Aligned. Score: 95.0348

CLUSTAL 2.1 multiple sequence alignment

```
XM_014205881.1      GGAGGAGGAAGTGGAGACCTTTGCCTTCCAGGCTGAGATCGCCCAGCTGATGTCCCTGAT
XM_014144832.1      GGAGGAGGAAGTGGAGACCTTTGCCTTCCAGGCTGAGATCGCCCAGCTGATGTCTCTGAT
                    ****************************************************** *****

XM_014205881.1      CATCAACACATTCTACTCCAACAAAGAGATCTTCCTCAGGGAGCTCATCTCCAACTCCTC
XM_014144832.1      CATCAACACTTTCTACTCAAACAAAGAGATTTTCCTTAGGGAGCTCATCTCCAACTCTTC
                    ********* ******** *********** ***** ******************** **

XM_014205881.1      AGATGCTTTGGACAAGATCAGATACGAGAGCTTGACAGACCCCACCAAATTGGATTCCTG
XM_014144832.1      AGATGCTTTGGACAAGATCAGATACGAGAGCTTGACAGACCCCACCAAATTGGATTCCTG
                    ************************************************************

XM_014205881.1      CAAGGACCTAAAGATCGAGGTCACCCCTGACCTGCGCACTCGCACCCTGACCTTGGTTGA
XM_014144832.1      CAAGGAGCTGAAGATCGAGATCACCCCTGACCTGCGCACTCGTACCCTGACCCTGGTTGA
                    ****** ** ********* ********************** ********* *******

XM_014205881.1      CACCGGCATCGGCATGACCAAGGCCGACCTGATAAACAACCTTGGAACCATCGCCAAGTC
XM_014144832.1      CACCGGCATCGGCATGACCAAGGCCGACCTGATCAACAACCTGGGAACCATTGCAAAGTC
                    ********************************* ******** ******** ** *****

XM_014205881.1      TGGCACCAAGGCCTTCATGGAGGCCCTGCAGGCTGGAGCTGACATCTCTATGATCGGGCA
XM_014144832.1      TGGCACCAAGGCCTTCATGGAGGCCCTGCAGGCTGGAGCTGACATCTCCATGATCGGGCA
                    ************************************************ ***********

XM_014205881.1      GTTCGGTGTGGGTTTCTACTCCGCCTACCTGGTGGCTGAGAGGGTGACTGTCATCACCAA
XM_014144832.1      GTTCGGTGTGGGTTTCTACTCTGCATACCTGGTGGCTGAGAGGGTGACTGTCATCACCAA
                    ********************* ** ***********************************

XM_014205881.1      GCACAACGATGATGAGCAGTACATCTGGGAGTCTGCAGCTGGTGGCTCCTTCACTGTCAA
XM_014144832.1      GCACAATGATGATGAGCAGTACATCTGGGAGTCTGCAGCTGGTGGCTCATTCACTGTCAA
                    ****** ***************************************** ***********

XM_014205881.1      AGTTGACACTGGTGAGTCCATTGGCCGTGGCACCAAAGTGATCCTGCACATGAAGGAGGA
XM_014144832.1      AGTTGACACTGGCGAGTCCATTGGTCGTGGCACCAGAGTGATCCTGCACATGAAGGAGGA
                    ************ *********** ********** ************************

XM_014205881.1      CCAGTTTGAATACTGTGAGGAGAAGCGCGTCAAGGAGGTTGTGAAGAAGCACTCCCAGTT
XM_014144832.1      CCAGTTTGAATACTGTGAGGAGAAGCGCGTCAAGGAGGTTGTGAAGAAGCACTCCCAGTT
                    ************************************************************

XM_014205881.1      CATTGGCTACCCCATCACACTCTATGTGGAGAAGTCTAGAGAGAAGGAGGTGGACCTTGA
XM_014144832.1      CATTGGCTATCCCATCACACTCTTTGTGGAGAAGTCTAGAGAGAAGGAGGTGGACCTTGA
                    ********* ************* ************************************

XM_014205881.1      GGAGGGAGAAAAGGATGAGGAGGCTGATAAAGATGCTGCAGCTGAGGACAAAGACAAGCC
XM_014144832.1      GGAGGGAGAAAAGGATGAGGAGGCTGATAAAGATTCTGCAGCTGAGGACCAAGACAAGCC
                    ********************************** ************** **********

XM_014205881.1      CAAGATCGAGGACGTCGGCTCTGATGAGGACGAGGACACCAAGGATAGCAAGAACAAGAG
XM_014144832.1      CAAGATCGAAGATGTCGGTTCTGATGAGGATGAAGACACCAAGGATTCCAAGAACAAGAG
                    ********* ** ***** *********** ** ************  ************

XM_014205881.1      GAAGAAGAAGGTCAAGGAGAAGTACATCGACGCTGAGGAGCTGAACAAGACCAAGCCTAT
XM_014144832.1      GAAGAAGAAGGTCAAGGAGAAGTACATTGACGCAGAGGAGCTGAACAAGACCAAGCCTAT
                    *************************** ***** **************************

XM_014205881.1      CTGGACCCGTAACCCTGATGACATCACCAATGAGGAGTATGGAGAGTTCTACAAGAGTCT
```

```
XM_014144832.1      CTGGACCCGTAACCCTGATGACATCACCAATGAGGAGTACGGAGAGTTCTACAAGAGTCT
                    *************************************** ********************

XM_014205881.1      GACCAACGACTGGGAGGACCACCTGGCTATCAAGCACTTCTCAGTGGAGGGCCAGCTGGA
XM_014144832.1      GACCAACGACTGGGAGGACCACCTGGCTATCAAGCACTTCTCAGTGGAGGGCCAGCTGGA
                    ************************************************************

XM_014205881.1      GTTCCGCGCTCTGCTCTTTGTGCCCAGGAGGGCTTCCTTCGACCTCTTTGAGAACAAGAA
XM_014144832.1      GTTCCGCGCTCTGCTTTTTGTGCCCAGGAGGGCTTCCTTCGACCTCTTCGAGAACAAGAA
                    *************** ******************************** ***********

XM_014205881.1      GAAGAAGAACAACATCAAGCTGTATGTGCGCAGGGTCTTCATCATGGACAACTGTGACGA
XM_014144832.1      GAAGAAGAACAACATCAAGCTGTATGTGCGCAGGGTGTTCATCATGGACAACTGTGACGA
                    ************************************ ***********************

XM_014205881.1      GCTGATGCCAGAGTATCTCAACTTCATCAAGGGTGTGGTGGACTCTGAGGATCTCCCCCT
XM_014144832.1      GCTGATGCCAGAGTATCTCAACTTCATCAAGGGTGTGGTGGACTCTGAGGATCTCCCCCT
                    ************************************************************

XM_014205881.1      GAACATCTCCAGAGAGATGCTGCAGCAGAGCAAGATCCTCAAGGTGATCCGCAAGAACTT
XM_014144832.1      GAACATCTCCAGAGAGATGCTGCAGCAGAGCAAGATCCTCAAGGTAATCCGCAAGAACCT
                    ********************************************* ************ *

XM_014205881.1      GGTCAAGAAGTGTATAGAGCTTTTCACAGAACTCTCAGAGGACAGAGATAACTACAAGAA
XM_014144832.1      GGTCAAAAAGTGTATGGATCTTTTCATCGAGCTCTCAGAAGACAAGGACAACTACAAGAA
                    ****** ******** ** *******  ** ******** ****  ** ***********

XM_014205881.1      GTTCTATGAGCAGTTTTCCAAGAACATCAAGCTGGGAATCCATGAGGACTCTCAGAACCG
XM_014144832.1      GTTCTATGAGCAGTTCTCCAAGAACATCAAGCTGGGAATCCATGAAGACGCTCAGAACCG
                    *************** ***************************** *** **********

XM_014205881.1      CAAGAAACTGTCAGACATGCTGCGCTACTACACCTCCAACTCCGGTGATGAAATGGTTTC
XM_014144832.1      CAAGAAGCTGTCAGACATGCTGCGCTACTACACCTCCAACTCCGCTGACGAAATGGTCTC
                    ****** ************************************* *** ******** **

XM_014205881.1      CCTGAAGGACTACGTTTCCCGCATGAAGGACACCCAGAAACACATCTACTACATTACTGG
XM_014144832.1      CCTGAAGGAGTATGTTTCTCGCATGAAGGACACCCAGAAACACATGTACTACATAACTGG
                    ********* ** ***** ************************** ******** *****

XM_014205881.1      TGAGACCAAGGAACAGGTCGCCAACTCTTCCTTTGTGGAGCGCCTCCGCAAGGCCGGCCT
XM_014144832.1      TGAGACCAAGGAACAGGTCGCCAACTCTTCCTTTGTGGAGCGCCTCCGCAAGGCCGGCTT
                    ********************************************************** *

XM_014205881.1      GGAAGTGATCTACATGATTGAGCCCATTGATGAGTACTGTGTCCAGCAGCTGAAGGAGTA
XM_014144832.1      GGAAGTGATCTACATGATTGAGCCCATTGATGAGTACTGTGTCCAGCAGCTGAAGGAGTA
                    ************************************************************

XM_014205881.1      CGATGGCAAGAACCTGGTCTCTGTGACCAAGGAGGGTCTGGAGCTGCCTGAGGATGAGGA
XM_014144832.1      TGATGGCAAGAACCTGGTCTCCGTGACCAAGGAGGGTCTGGAGCTGCCTGAGGATGAGGA
                     ******************** **************************************

XM_014205881.1      TGAGAAAAAAAAACAGGAGGAGCTGAATTCTAAATTTGAGAACCTCTGCAAGATCATGAA
XM_014144832.1      TGAGAAGAAGAAGCAAGAGGAGCTGAACACTAAATTTGAGAACCTCTGCAAGACAATGAA
                    ****** ** ** ** ***********  ************************  *****

XM_014205881.1      GGACATCCTGGACAAGAAAATTGAGAAGGTTTCAGTGTCCAACCGCCTGGTCTCCTCCCC
XM_014144832.1      GGACATCCTGGACAAGAAGATTGAGAAGGTTTCAGTTTCCAACCGCCTGGTCTCCTCCCC
                    ****************** ***************** ***********************

XM_014205881.1      CTGCTGCATTGTGACCAGCAACTACGGGTGGACAGCCAACATGGAGAGGATCATGAAATC
XM_014144832.1      CTGCTGCATCGTCACCAGTACATACGGCTGGACGGCCAACATGGAGAGAATCATGAAATC
                    ********* ** ***** *  ***** ***** ************** ***********

XM_014205881.1      TCAAGCTCTCAGAGACAACTCCACCATGGGCTACATGACAGCCAAAAAGCACCTGGAGAT
XM_014144832.1      TCAAGCTCTCAGAGACAACTCCACCATGGGCTACATGACAGCCAAGAAGCACCTGGAGAT
                    ********************************************* **************

XM_014205881.1      CAACCCAACCCACCCTATTGTCGAGACCCTGAGAGAGAAAGCTGAGGCCGACAAGAACGA
```

```
XM_014144832.1      CAACCCAACCCACCCTATTGTCGAGACTTTGAGAGAGAAAGCTGAGGCTGACAAGAACGA
                    ***************************  ******************* ***********

XM_014205881.1      CAAAGCCGTGAAGGATCTGGTCATCTTGCTGTTCGAGACTGCTCTGATGTCTTCTGGATT
XM_014144832.1      CAAAGCTGTAAAGGACTTGGTCATCTTGCTGTTCGAGACTGCTCTATTGTCTTCTGGGTT
                    ****** ** *****  ****************************  ********** **

XM_014205881.1      CACACTGGACGACCCTCAGACCCACGCAAATCGCATCTACAGGATGATCAAGCTTGGCCT
XM_014144832.1      CACGCTGGACGACCCTCAGACCCATGCAAACCGCATTTACAGGATGATTAAGCTTGGCCT
                    *** ******************** ***** ***** *********** ***********

XM_014205881.1      GGGCATCGATGATGATGACTCAGCAGTGGAGGACATCCTCCAGCCCAGTGAGGATGACAT
XM_014144832.1      GGGCATCGATGGTGATGACTCAGCAGTGGAGGAAATCCTCCAGCCCAGTGAGGATGACAT
                    *********** ********************* **************************

XM_014205881.1      GCCTGTCCTGGAGGGAGATGATGACACCTCTAGAATGGAGGAAGTTGACTAAAGA
XM_014144832.1      GCCTGTCTTGGAGGGAGATGATGACACATCAAGAATGGAGGAAGTTGACTAAAGA
                    ******* ******************* ** ************************
```

CLUSTAL 2.1 Multiple Sequence Alignments for *ambra1a*

```
Sequence 1: XM_014175106.1 (ambra1aa) 5849 bp
Sequence 2: XM_014126406.1 (ambra1ab) 9647 bp
Sequence 3: XM_014147648.1 (ambra1ac) 9981 bp
Sequence 4: XM_014123983.1 (ambra1ad) 4926 bp
```

```
Sequences (1:2) Aligned. Score: 66.5584
Sequences (1:3) Aligned. Score: 40.8275
Sequences (1:4) Aligned. Score: 48.3151
Sequences (2:3) Aligned. Score: 29.7605
Sequences (2:4) Aligned. Score: 45.7775
Sequences (3:4) Aligned. Score: 70.2598
```

```
CLUSTAL 2.1 multiple sequence alignment


XM_014175106.1      ------------------------------------------------------------
XM_014126406.1      AATAGCGATAAATGTAGATGAACAATACATTGCTTACTTTAAAGCAAAAGTGACCCTTTG
XM_014147648.1      ------------------------------------------------------------
XM_014123983.1      ------------------------------------------------------------


XM_014175106.1      ------------------------------------------------------------
XM_014126406.1      TAAACTAGCTATTTATTGCACTGAAAAAAAAGCTCCCTGTTTGACGCCCACCTAGGTTCC
XM_014147648.1      ------------------------------------------------------------
XM_014123983.1      ------------------------------------------------------------


XM_014175106.1      ------------------------------------------------------------
XM_014126406.1      TGTGTTCAACTAGTAATGCCTGATGTGCTGTGGCAGGTCTGATCTGGCCAGCTGGCTAAT
XM_014147648.1      ------------------------------------------------------------
XM_014123983.1      ------------------------------------------------------------


XM_014175106.1      ------------------------------------------------------------
XM_014126406.1      GTTTTGGAGAACACAGGCTAATAGCTAGCCTAGCTACGCTGCTAGTTTGCTAACCACAGT
XM_014147648.1      ------------------------------------------------------------
XM_014123983.1      ------------------------------------------------------------


XM_014175106.1      ------------------------------------------------------------
XM_014126406.1      CGGTCAACACAATATAAAAACACCGGAGAAAATAAAACATAAATATATGAGACAGTTTCA
XM_014147648.1      ------------------------------------------------------------
XM_014123983.1      ------------------------------------------------------------


XM_014175106.1      ------------------------------------------------------------
XM_014126406.1      GGTGCTAGTAAAAGTCTAATATATGTAACTAGTTAAGTCGTCAAGAACATCCAACCGTAG
XM_014147648.1      ------------------------------------------------------------
XM_014123983.1      ------------------------------------------------------------


XM_014175106.1      ------------------------------------------------------------
XM_014126406.1      CAGTGGGAAAAGGTAGGGACGTGGTGGAATATATCGCTAACGTTATCTAGTTTGGCCAAA
XM_014147648.1      ------------------------------------------------------------
XM_014123983.1      ------------------------------------------------------------


XM_014175106.1      ------------------------------------------------------------
XM_014126406.1      TGCCTTGTTTGGGTTGGTTGTCAACGCTAGCTCTTACTGGAGTTGGACAAACGCTGGCAT
XM_014147648.1      ------------------------------------------------------------
XM_014123983.1      ------------------------------------------------------------
```

```
XM_014175106.1      ------------------------------------------------------------
XM_014126406.1      GTTAGCTCGCTAGTTTATTAGGAGATGGGTAGTTACAGATTGTAACGGTTCACAACGCTA
XM_014147648.1      ------------------------------------------------------------
XM_014123983.1      ------------------------------------------------------------


XM_014175106.1      ------------------------------------------------------------
XM_014126406.1      GCTCTTGAGTTGGACAAACGCAGGCATGTTAGCTTGCTAGTTTATTGGGAGACGGGTAGC
XM_014147648.1      ------------------------------------------------------------
XM_014123983.1      ------------------------------------------------------------


XM_014175106.1      --------------------------------AATGACAACCATAAGAACTATTATTATT
XM_014126406.1      TACAGATTGTAAGACAATGTGATTTAACCAAATATTATACTGGTAGTTACAGGCCTGGCT
XM_014147648.1      ------------------------------------------------------------
XM_014123983.1      ------------------------------------------------------------


XM_014175106.1      GTATAAACCGTTTGG--ATGAGGACCAG-------GATCCTATG---GTCCAGACCCAG
XM_014126406.1      GTACAAAACGCCTAATAACTTGAACCTGTTATTGGCGATGCTATCTTCGTCCTCCACCAG
XM_014147648.1      ------------------------------------------------------------
XM_014123983.1      ------------------------------------------------------------


XM_014175106.1      TTTGGAAAAC-------GCTGCGTTGTATAATCATTAACTTATATGTATACATGTTTTCT
XM_014126406.1      TATAATGAACCACATTTGTTGGGTTAAATCG-CATTATCTGGTTTTACAATCTGGAGTTA
XM_014147648.1      -------------------TTTGCGATCTGGGCTCTG-CTCATGTGACTGTCTG--TTTC
XM_014123983.1      -----------------------CACGCTAAAAA-TG-TTCACGACAC-ACATC--TCTG
                                                *      *   *            *

XM_014175106.1      GTAAAACATAGAAATAGCGATACATGTAGATTATA-AATACATTGCTTAC----------
XM_014126406.1      GGAGACCGGTAGTTTAACTAGATTTTTAAGTTTCATGACAGCCTGTTTTCGTTG--TTTC
XM_014147648.1      CTAAACTGGGGA-GCAG-TAGACGGATAGCTAGCATAAAGAATTAGCGCCGTGC-AAGAA
XM_014123983.1      GGAAGCTAGCTACGCAG-TAGACGGATAGCTAGCACAATGAATTTGCTCTATGCCAGGAA
                      *            *   * *    **  *   *  *     *

XM_014175106.1      TTTAAAGCAAAATTGACCCTTTGTAAGCTA-GCTATTTCTTGCACTGCAAAAAAAGCAAT
XM_014126406.1      TCTGTGTTAAAGTTTTTCTGGCAGAACCCACACCACCAGTTGTTTGGTCATTAACGTTAT
XM_014147648.1      ATTATGTTGAAATAGCTTTTCCAGAGGATATGA----ATGTAATCAATCGCAAGTATAAT
XM_014123983.1      AATATGATGAAATAGATTTATCAGAGGATATGA----ATGTGATCAATCGCAATTATCAT
                      *      ** *           *    *          *           *     **

XM_014175106.1      ----CGGTTTTGTGGCGCCCACCTATGTTCCTGTGTTCA-ACTAGTAATGCCTG-ATGAT
XM_014126406.1      GTTGTAATTCTGTAAAACGAACAGGTTTTCAT-TATTCATGCCACCAATGGCAACATTGT
XM_014147648.1      -----AGATTGTTATTTGTCAATCATATTGGTAAATGCA---TGAATGTGTCAAAGCTAA
XM_014123983.1      -----AGATTGATATTGGTCAATT-CATTAATGTGT-CG---AGGCTAAGACAAGTGTAG
                            *   *       *      **  *   * *           * *

XM_014175106.1      CTGTGGCTGGTCTGGCCAGCTGGCTAATG----TTTTGGAGAACA--TAGCTAAT---AG
XM_014126406.1      CATCAGCTCCTCAGGACTGTTAACAAATGAGAACTGCAGTGATTAGGTAACTAAT---AA
XM_014147648.1      GTGTAGCCAGTACTCCCCGTTCAGTTTTGCTTGCTAGCGA-ATCAGCTAGCTAGTCTCAG
XM_014123983.1      CA--AGCTAGTACTTGCCGTACATTTTTGCTTGCTACGCT-AACAGTTAGCTA-------
                         **   *     * *         **     *      *  *  ** ***

XM_014175106.1      CT------AGCC----TAGCTACACTGCTAGCT-TGTTAACCACAGTTAACACAATATAA
XM_014126406.1      TTGTTTTTAGCTAGT-TAGTTACAAACATGGTTCTGTCATCAACAGTTGG-ATTTCATCA
XM_014147648.1      CTAGCTTTAGTTGGTCCGCCTACGATGACAAAT--TTCAACAGAAAT--GCAATAAATAA
XM_014123983.1      ----------------CGCCTATGATGACACAT--TTCAACAAGAAT--ACAAGAAAGAA
                                        **          *   * * *   * *    *    *  *

XM_014175106.1      AAACATCGGAGAAAATAGA----CCATA----ATTATACGAGATAGTTTCAGGTGCTAGT
XM_014126406.1      TGCTATGGCAAGGTGAAGG----TGTAG----ATTACAAGCTAGACTCCCCTTTAG-ACT
XM_014147648.1      AGCAAGGATGAAATATGCTGCTTTCTGGCGCTGTCGTTGGTTAGGGACCATCGTAAAAGT
XM_014123983.1      AGCCTAGAC--------------TCTG----CATGGCGAGCGAGTGGTGGCAGCAGAAGT
                                                     *     *  *              * *

XM_014175106.1      AAAT-GTCGAATATATGTAACTTGCTA-AGTCGACAAGAACATC-CAACCGTAGCAGTGG
XM_014126406.1      ACAA-GCTGGCCACACACACTTTCATATGGCCTTTGCTTAGACC-CTTTGGTGACTGAGA
```

```
XM_014147648.1      GCAATACAGCCTCTGCATGGCGAGCTA--GCAGCAGCAGAAGTTACTCTCCCTAGAGCAG
XM_014123983.1      G----ACTGTCCCTAGA------------GCAGCCAGAGAGGC--------------CAG
                            *                    *         *

XM_014175106.1      GAAAAGCTAACTAGTTAGCTACAAACATGGTTATGTCATCAACAGCTGGATTTCACCATG
XM_014126406.1      CCAACAAACACCAGTGTGAAGGAGAGGGAGGAAGGCC-TGATC--TTTGAGATGACTGT-
XM_014147648.1      GCAGAGAGCAGTGGTGTGGCCCCGGCCTGTACAGGAGGAGAAC--CATGGCCTC-TCGT-
XM_014123983.1      TGGTAGAGTGGTGGTGTGGCCCCGTACGGGA--GGAGAACCAC--CATGGCCTC-TCGT-
                                 **  *                *       *     *   *     *

XM_014175106.1      CAATGGCAAGAATTCGGTGCGGATCTTAACTGCCCGGGAGCGTGGGGCACGTGGCCTTGG
XM_014126406.1      CTACCCAAAGAATTCAGTGCGGATCTTATCTGCCCGGGAGCGTGGGGCCCGTGGCCTTGG
XM_014147648.1      -CATCAAAGGAACTCTGTGCGGATCCTGTCCACCCGGGAGCGCGGCTCGCAGGCCTTTGG
XM_014123983.1      -CATCAGAGGAACTCTGTGCGGATCCTGTCCAGCCGGGAGCGCGGCTCACAGGCCTTTGG
                      *    * *** ** ********* *  *   ********* **  * *  * * ****

XM_014175106.1      CTCCCAGCGCCTCCTGCAGCAGCTTGTTGAGGAGAAGACCCGGCGGATGAAATGGCAGAG
XM_014126406.1      CTCCCAGCGCCTCCTGCAGCAGCTTGTTGAAGAGAAGACCCGGAGGATGAAATGGCAGAG
XM_014147648.1      CTCCCAGCGCCTCCTGCAGCAGATGGTGGAGGAGAAGGTCCGCTGGATGAAATGGCAGAG
XM_014123983.1      CTCCAAGCGCCTCCTGCAACAGCTGGTGGAGGAGAAGGTCCGCTGGATGAAATGGCAGAG
                    **** ************* *** * ** ** ******  ***  ****************

XM_014175106.1      TCAGAAAGTGGAGTTGCCGGACAGCCCTCGTTCTACCTTCCTGTTGGCTTTTAGCCCGGA
XM_014126406.1      TCAGAAAGTGGAGTTGCCGGACAGCCCTCGTTCTACCTTCCTGTTGGCTTTTAGCCCGGA
XM_014147648.1      TCAGAAAGTAGAGCTGCCAGATAGCCCTCGCTCCACGTTCCTGTTGGCCTTCAGCCCAGA
XM_014123983.1      TCAGAAAGTGGAGCTGCCAGACAGCCCTCGATCGACGTTCCTGTTGGCCTTCAGCCCAGA
                    ********* *** **** ** ******** ** ** *********** ** ***** **

XM_014175106.1      CAGGACCTTAATTGCCTCCACTCATGTCAACCATAACATTTATATCACCGAGATTAAGAC
XM_014126406.1      CAGGACCTTAATTGCCTCCACTCATGTCAACCATAACATTTATATCACCGAGATTAAGAC
XM_014147648.1      CCGGACCCTCATGGCTTCCACTCACGTCAACCACAACATCTACATCACGGAGGTGAAGAC
XM_014123983.1      CCGGACCCTCATGGCTTCCACTCATGTCAATCACAACATCTACATCACAGAGGTGAAGAC
                    * ***** * ** ** ******** ***** ** ***** ** ***** *** * *****

XM_014175106.1      AGGGAAGTGTGTTCACTCTCTGGTGGGCCATCGCCGCACTCCTTGGTGTCTGACCTTTCA
XM_014126406.1      AGGGAAGTGTGTTCACTCTCTGGTTGGCCATCGCCGCACTCCTTGGTGTCTGACCTTTCA
XM_014147648.1      TGGAAAGTGCCTGCATTCGCTGGTGGGACACCGCCGCACACCCTGGTGCGTGACCTTTCA
XM_014123983.1      TGGAAAGTGCTTGCATTCGCTGGTAGGCCACCGCCGCACGCCCTGGTGCGTGACCTTTCA
                     ** *****  * ** ** ***** ** ** ******** ** *****  **********

XM_014175106.1      CCCTACCATCCCTGGTTTGGTGGCTTCAGGATGTCTGGATGGAGAGGTTCGCATCTGGGA
XM_014126406.1      CCCTACCATCCCTGGTTTGGTGGCTTCGGGATGTCTGGATGGAGAGGTTCGCATCTGGGA
XM_014147648.1      CCCTACCATCCCCGGCCTGGTGGCCTCCGGGTGCCTTGACGGAGAGGTCCGCATCTGGGA
XM_014123983.1      CCCTACCATCCCAGGTCTGGTGGCCTCCGGGTGCCTTGACGGAGAGGTCCGCATCTGGGA
                    ************ **  ******* ** ** ** ** ** ******** ***********

XM_014175106.1      CCTGCATGGTGGCAGTGAGAGCTGGTTCACTGAGAGCAATGTGGCCATTGCCTCCCTGGC
XM_014126406.1      CCTGCATGGTGGCAGTGAGAGCTGGTTCACTGAGAGCAATGTGGCCATTGCCTCCCTGGC
XM_014147648.1      CCTGCATGGTGGCAGTGAGAGCTGGTTCACAGAGAGCAACGTTGCCATTGCCTCGCTTAC
XM_014123983.1      TCTGCATGGTGGCAGTGAGAGCTGGTTCACGGAGAGCAACGTGGCCATTGCCTCGCTAGC
                     ***************************** ******** ** *********** **  *

XM_014175106.1      CTTTCACCCTACTGCCCAGCTCCTCCTTATAGCAACAAACAATGAGGTTCACTTCTGGGA
XM_014126406.1      GTTTCACCCTACTGCCCAGCTCCTTCTCATAGCAACAAACAATGAGGTTCACTTCTGGGA
XM_014147648.1      CTTCCACCCCACCGCCCAGCTCCTCCTCATCGCCACCAACAATGAGCTACACTTCTGGGA
XM_014123983.1      CTTCCACCCCACTGCCCAGCTCCTCCTCATCGCCACCAATAACGAGCTCCACTTCTGGGA
                     ** ***** ** *********** ** ** ** ** ** ** *** * ***********

XM_014175106.1      CTGGAGTAGGAGGGAGCCATTTGCCCTGGTCAAGACTGCCAGCGAGACAGAGAGAGTCAG
XM_014126406.1      CTGGAGTAGGAGGGAGCCATTTGCCCTGGTCAAGACTGCCAGCGAGACAGAGAGAGTCAG
XM_014147648.1      CTGGAGTCGGCCCGAGCCCTTCGCCGTGGTCAAAACCGGCAGCGAGACTGAAAGAGTCAG
XM_014123983.1      CTGGAGCCGGCCCGAGCCCTTCGCCGTGGTCAAAACTGGCAGCGAGACTGAAAGAGTCAG
                    ******  **   ***** ** *** ******* ** * ********* ** ********

XM_014175106.1      ---------------ACTGGTGAGGTTTGACCCACTGGGTCATTACCTGTTGACCGCTAT
XM_014126406.1      ---------------ACTGGTGAGGTTTGACCCACTGGGTCATTACCTGTTGACCGCTAT
XM_014147648.1      CTCCATCTCTGTCAGGTTGGTGAGGTTTGATCCTTTGGGGCACAATCTTCTGACGGCTAT
XM_014123983.1      ---------------GCTGGTGAGATTTGATCCTTTGGGGCACAATCTTCTGACTGCTAC
```

```
                                     ******* ***** **  **** **  * **  **** ****

XM_014175106.1      AGTGAATCCCTCTAACCAACAGCAGAGTGATGATGACTCTGAGGTCCCCATGGATAGCGT
XM_014126406.1      AGTGAATCCCTCTAACCAACAG---AGTGATGATGACTCTGAGGTCCCCATGGATAGCAT
XM_014147648.1      TGTGAACCCCTCCAACCAGCAG---AATGATGATGACTCCGAGGTCCCCATGGACAGTGT
XM_014123983.1      TGTGAACCCCTCCAACCAGCAG---AATGATGATGACTCGGAGGTCCCCATGGACAGCGT
                     ***** ***** ***** ***   * ************ **************  **  *

XM_014175106.1      GGAGATGCCCCACTTTCGCCATCGATCCTTCCTACAGTCCCAACCGGTCCGCCGTACCCC
XM_014126406.1      GGAGATGCCCCACTTTCGCCATCGATCCTTCCTACAGTCCCAACCGGTCCGCCGTACCCC
XM_014147648.1      AGAGATGCCCCACTTCCGCCAGCGCTCCTTCCTGCCCTCCCAGCCTGTACGCCGCACGCC
XM_014123983.1      GGAGATGCCCCACTTCCGCCAGCGCTCCTTCCTGCCCTCCCAGCCTGTACGCCGCACGCC
                     ************** ***** ** ******** *  ***** ** ** ***** ** **

XM_014175106.1      CATTCTCCACAACTTCCTGCACATCCTGTCATCGAGGAACTCCGGCTCACAGTCTGAGGA
XM_014126406.1      CATTCTCCACAACTTCCTGCACATCCTGTCATCGAGGAACTCAGGCTCACAGTCTGAGGA
XM_014147648.1      AATCCTCCACAACTTCCTGCACATCCTGTCATCGCGCTCGTCGGGGGCACAGGCTGGGGG
XM_014123983.1      CATCCTCCACAACTTCCTGCACATCCTTTCGTCGCGCTCCTCGGGGGCGCAGGCCGGGGG
                     ** *********************** ** *** *    ** **  * *** * * **

XM_014175106.1      ACAGCATCCTCCTGCACCCGCTGCCAATAGTGCTGCTGACTCTCCTAGTCTTCCCTCTGG
XM_014126406.1      ACAGCATCCTCCTGCACCCGCTGTCAATAGTGCTGCTGACTCTCCTAGTCTTTCCTCTGG
XM_014147648.1      GGAT--GCGTCCCGC-CCTGGTGGGGAGGACAGCGGCGAGCCCCCCAGCATGCCTTTGGG
XM_014123983.1      GGAT--TCGTCCCGC-CCTGGCGGGGAGAGCAGCGGCGAGCCCCCCAGCATGCCTTTGGG
                      *    * *** ** ** *  *   *       *  **  * ** **  *  * *  **

XM_014175106.1      ACGTTATTCTGCCCTGCGGAATCGCTCCCGACTCCCCTACCAGGGTTGTGTGCAACACCT
XM_014126406.1      ACGTTATTCCGCCCTGCGTAATCGCTCCCGACCTCCCTACCAGGGTTGTGTGCAACACCT
XM_014147648.1      CCAGTACCCAGCCTCGCAGGACCT---TGGACTGCCCTACCCAGGTTGCACCCAGCACCT
XM_014123983.1      CCAGTACCCAGCCTCGCAGGACCG---TGGACTGCCCTTCCCGGGCTGCACCCAGCACCT
                     *  **  * ***  **   * *      ***  **** **  ** **    ** *****

XM_014175106.1      TGGTATGGTTTGCTTCTGCAGTCGGTGCTCTGCCACCCGAGCCCCTTCACCATCTGGAGA
XM_014126406.1      TGGTATGGTTTGCTTCTGCAGTCGGTGCTCTGCCATCCAAGCCCCTTCACCATCTGGAGA
XM_014147648.1      GGGCATGGTGTGCCTCTGCAGCCGCTGCATGGCCAACCGCAGCCCCTCTCTGGGGGAGGG
XM_014123983.1      GGGCATGGTGTGCCTATGCAGCCGCTGCACAGCCAACCGCAGCCCCTCTCTGGGCGAGGG
                     ** ***** *** * ***** ** ***   **** **    *** ** *     *  *

XM_014175106.1      GGACCC---CTCTGACCCTGAGA---GCCTGGAGGC-------------------CCAGT
XM_014126406.1      GGACCC---CTCTGACCCGGAGA---GCCTGGAGAC-------------------CCAGT
XM_014147648.1      TCCTTCCACCTCCAGGCCTGTTGTCCACCCCGAGCTTCCCCAGGTGGCCTCTCAGCCAGC
XM_014123983.1      GCCCTC---CTCCAGGCCTGTGGTCCTCCCTGAGCACCC------------TCAGCCGGC
                         *   ***    ** *       **  ***                     ** *

XM_014175106.1      CTC--ATGCCTCCACCTTCTCCTCTGCTCGCACTGAGCCCCGGCAGCCCTCTGAGCC---
XM_014126406.1      CCT--ATGCCTCCACCTTCTCCTCTGCTCGCACTGAGCCCCGGCAGCCCTCTGAGCC---
XM_014147648.1      CCCCCACGCCTCCACCTTTTC---GGCCCGTACGGAGCCCCGGCAGCCTTCAGAGCCCAC
XM_014123983.1      CCCCCACGCCTCCACCTTTTCCTCAGCCCGTACGGAGCCCCGACAGCCTTCAGAGCCCAG
                    *    * *********** **    ** ** ** ******** ***** ** *****

XM_014175106.1      TCGCTCCTCACACCGTCCATCTGCGTTCAGCAGTGTGTACGGCAGTGCTAGAGGACACTC
XM_014126406.1      TCGCTCCTCACACCGTCCCTCTGCCTTCAGCAGTGTGTACAGCGGTGCTAGAGGACACTC
XM_014147648.1      TGGAGCCCAGCAGCGGCCCTCAGCCTTCACCACAGTCTACTACAGTGCCGGCAGCTCCCT
XM_014123983.1      TGGGGCCCAACAGCGGCCCTCTGCCTTCACTACAGTCTACTACAGCGCCGGCAGCTCCCT
                    * *  **   ** ** ** ** ** ****  *  ** ***  * * **  *  *   *

XM_014175106.1      TTTACGCACTCCATCTTCAGGCCC--TGGCCAGCGCAGCATCCCAGGCCTTATAACCAAC
XM_014126406.1      TTTACGCACTCCATCTTCAGGCC-------------------------------------
XM_014147648.1      GCACCGCGCTGTGCCTACCACCCACGTGGCCAGCCAGCCCCTGAGTCAGCAGCTGTTGCC
XM_014123983.1      GCACCGTGCCGTGCCTACCGCCCACGTGGCCAGCCCGCCCCTGAGCCAGCAGCCTCCGCC
                        **  *     ** *   **

XM_014175106.1      CAGGGCCACCAGCAGCCAGG-CCGGGAGTGCTCTGGGCGACTGGCGGGGGCAGACTGGAT
XM_014126406.1      -------ACCAGCAGCCAGG-CCGGGAGTGTTCTGGGCGACTGGCGGGGGCAGACTGGAT
XM_014147648.1      TACGCCCGGCAACCACCAACACCAACCCCCATCCCGCCCTCTGCCCGGACCTGACTGGAC
XM_014123983.1      GTCACCCACCAGCCACCAAC------CCCCCTCCCGCCCACTGCCCGGACCCGATTGGAC
                             ** *  ***             **  * *  *** * **  * ** ****
```

```
XM_014175106.1      GGGCAGCATGCTCAACATGCG-----------------------------------CCCT
XM_014126406.1      GGGTAGCATGCTCAACATGCG-----------------------------------GCCT
XM_014147648.1      ACGCAGCCTGTTAAATATGAGAAGCTCAGAGAGCGGGGCCGGAAG---------TGGGGG
XM_014123983.1      ACGCAGCCTGCTGAACATGAGAGGCTCAGACAGCAGGACTGGAGGGAGTGAAGGTGGGGT
                      * *** ** * ** *** *

XM_014175106.1      GAGC-GTAGTGGGGGTGT---------------TGGGGTATCTCCACCCAGGACTAGTGC
XM_014126406.1      GAGC-GTAGTGGGAGTAT---------------TGGGGTATCTCCACCCAGGACTAGTGC
XM_014147648.1      GAGCAGTGGAGGGGGTATGGGTGGTGTTGGGGGTGGGATGCTGCCCCCCCGGACCAGCTC
XM_014123983.1      GAGCGGGGGAGGTGGTATGGGTGGTGTCGGGGGTGGGATGCTGCCCCCCCGAACCAGCTC
                    **** *  * **  ** *               **** *    ** *** * ** **  *

XM_014175106.1      ATCCTCTGTCAACCTGCTGTCTGTGCTGAGGCAGCAGGAGGGCTCATTTCAGTCGCCTGT
XM_014126406.1      ATCGTCTGTCAGCCTGCTGTCTGTGCTGAGACAGCAGGATTGCTCTTATCAGTCGCCTGT
XM_014147648.1      GTCCTCTGTCAGCCTGCTGTCAGTGCTGCGGCAGCAAGATGGCTCCTCCCAGTCCCCCGT
XM_014123983.1      GTCCTCCGTCAGCCTGCTGTCAGTGCTCCGGCAGCAGGACGGCTCCTCCCAGTCCCCCGT
                     ** ** **** ********* *****  * ***** **  **** *  ***** ** **

XM_014175106.1      CTACACCTCTGCCACAGAGGGAGGAAGCTTTCCCCC--AGC--AGTAGTGAGC----CCT
XM_014126406.1      CTACACCTCTGCCACAGAGGGAGGAAGCTTTCCCCC--AGC--AGTTATGAGC----CCC
XM_014147648.1      CTACACTTCTGCCACTGAGGGACGAGGCTTCCCCCCGCAGCCGGGCGCCGAACTTGGCTC
XM_014123983.1      CTACACTTCTGCCACAGAGGGACGGGGTTTCCCCACGCAGTCGAGCTCTGAACCGGGCTC
                    ****** ******** ****** *  * ** *** *  **    *    ** *    *

XM_014175106.1      CACACCAGTGG--AAACGGCTCT------------GTAGATG-----GACCCAGCACTAG
XM_014126406.1      CACACCAGTGG--AAACAGCTCT------------GTAGATG-----GACCCAGCACTAG
XM_014147648.1      CAGCCCTGTGGCCAATCAGAGAGGAGGAAGTGGTGGTGGAGGAGGAGGGGTGGGCTCCAG
XM_014123983.1      TGGCCCCGTGGCCAATCAGCGAGG------------TGGTGGAG------GGGCGGGCACCAG
                        ** ****  ** * *                ** **       *     ** * **

XM_014175106.1      TAGTGGACACCACTCTCTTGGGGAGGGAGGGAGCAACAGCCCCACCTCTATCCGTAACGT
XM_014126406.1      TAGTGGACACCACTCTCTTGGGGAGGGAGGGAGCAACAGCCCCACCTCTATCCGTAACGT
XM_014147648.1      CAGTGGGCACCACCCTTTCTGGGATGGCACGCGCAGCAACCCGGCCTCGTTCCGCAATGT
XM_014123983.1      CAGCGGGCACCACCCTTTCTGGGACGGCACGCGCAGCAACCCGACCTCGTTCCGCAACGT
                     ** ** ****** ** *  **** **   * *** ** ***  ****  **** ** **

XM_014175106.1      GCTGCAGTGCAACTTGAATCGCTACTTCATGGAGTATGATCGCATGCAGGAAATGGAGCA
XM_014126406.1      GCTGCAGTGCAACTTGAATCGCTACTTCATGGAGTATGATCGCATGCAGGAAATGGAGCA
XM_014147648.1      GCTCCAGTGCAATCTTAGCCGCTACTTCATGGAGTTCGACCGCATGCAGGACCTGGAGTT
XM_014123983.1      GCTCCAGTGCAACCTGAGCCGCTACTTCATGGAGTTTGACCGCATGCAGGACCTGGAGTC
                    *** ********  * *  ****************  ** ***********  *****

XM_014175106.1      GTCA---GGTGGTG------------CTGGTGGAGAGAGCAGCCAAGAGCAACAGACACG
XM_014126406.1      GTCA---GGTGGCG------------CTGGTAGAGAGAGCAGCCAAGAGCAACAGACACG
XM_014147648.1      GCCGCTGGGGGCCGCCAGCGTGGATGTCGGGGGAGGGGAGGGCGGCCAGGAGCAGATGCA
XM_014123983.1      GCCGCTGGGGGGCG------------TCGGGGGAGGGGAGGGCGGCCAGGAACAGACGCA
                    * *    ** *  *              **  *** *    **    ** * ****  *

XM_014175106.1      GGAGATGCTCAATAATAACATAGACTGTGAGCGGCCT--GTCACCC-------CCCATTA
XM_014126406.1      GGAGATGCTCAATAATAACATAGACTCTGAGCGGCCT--GTCATGC-------CTCATTA
XM_014147648.1      GGAGCTGCTCAACAACAACATAGACCCAGAGAGACCCGGGCCCTCCGTGTCCTCCCACTA
XM_014123983.1      GGAGCTGCTCAACAACAACATGGACCCGGAGAGACCCAGGCCTTCC---TCCTCCCACTA
                    **** ******* ** ***** ***   *** * **   * *   *       * ** **

XM_014175106.1      TCAGCCCC-----CCAACAGC-------AGTGATGGTGGTTCTGGTCCCT----------
XM_014126406.1      TCAGCCCC-----CCCACAGC-------ATTGATGGTGGTTCTGGTCCCT----------
XM_014147648.1      CCAGCCCCAGCCACCCCCACCTGCCCCTACCGAGAACAACCCCCCTCCCTTCTCTAGCGC
XM_014123983.1      TCAGCCCCAGCCGCCCCCACCAACCCCAGCCGAGAACAACCCCCCTCCCT---CCAATGC
                     *******     **  ** *          **        *   *****

XM_014175106.1      -----------CCCGTGGCCATATGAACCGCTGCAGGGTCTGCCACAACTTGTTCACGTT
XM_014126406.1      -----------CCCGCGGCCATATGAACCGCTGCAGAGTCTGCCACAACCTGTTCACATT
XM_014147648.1      CGCCCACTCCTCCCGGGGCCACCTGAACCGCTGCCGGGCCTGCCACAACCTGCTCACCTT
XM_014123983.1      GGCTCACTCCTCTCGGGGCCAACTGAACCGCTGCCGGGCCTGTCACAACCTGCTCACATT
                               * ** *****  *********** * * *** ****** ** **** **

XM_014175106.1      TAACCAAGGTACACAGCGCTGGGAGCGCACCGGCCAGACCCCATC---------------
XM_014126406.1      TAATCAAGGTACACAGCGCTGGGAGCGCACCGGCCAGACCCCATC---------------
```

```
XM_014147648.1      CAACCACGACTCCCAACGCTGGGAGCGCACCAGCCCAGCCTCCTCCACCTCCGCTGTCTC
XM_014123983.1      CAACCATGACTCCCAACGCTGGGAGCGCACCAGCCCAGCCTCGTCCACCTCCGCTGTCTC
                     ** ** *    * ** *************** ***    ** * **

XM_014175106.1      ---ACAAGAGGAGGGGAGCACCTCATGGCAGCCCCCAAGCCCCAACTCTGCCTTTCACAC
XM_014126406.1      ---ACAAGAGG---GGAGCACCTCATGGCAGCATCCAAGCCCCAACCCTGCCTTTCACAA
XM_014147648.1      CACGCAGGAGGGCTCCAGCTCTCCCTGGCGGCAGCC---CCCCAGCCCTGCCTATGAGGA
XM_014123983.1      CACCCAGGAGGGCTCCAGCTCTCCCTGGCAGCAGCC---TACCAGCCCTGCCTATGAAGA
                        ** ****     *** *  * **** **  **      *** * ****** * *

XM_014175106.1      ----AATGAGGCAG-GCGGTGCCA----CATGTTCCCCAGTCCTCTGACAGG---AGACA
XM_014126406.1      ----CATGAGGCAG-ACTGTGCTA----CAGGATCCCCAGTCCTCTGACAGA---AGACA
XM_014147648.1      GCCCCATGCATCATCACGGAGCCGGGATCCACACCCCAACCCCAAGAGAGGGAGAGGAG
XM_014123983.1      GACCCATGTGTCACCACAGAGGCGGGAGCCCCAACCCCAACCACCAGAGAGGGAGAGGAG
                         ***   **    * * *       *     *****  *     ** **    **

XM_014175106.1      GTTAGCACAGCCAC---AGCCCAGTG-----------CTGATGAACCTGGAGG---GGA
XM_014126406.1      GTTAGCACAGATAC---AGCCCAGTG-----------CTGATGAACCTGGAGG---GGA
XM_014147648.1      GGTAGCCCAACCCCCTGAGCCCAGCGAGCTACAGCCCTCCCCAGGCCCTGACGTTGCGGC
XM_014123983.1      GGTAACCCAGCCCCCGGAGCTCAGCGAGCGGCAGCCCTCCCCAGGCCCTCATGGTGCGGC
                    * ** * **     *    *** *** *             *     *   ***    *     **

XM_014175106.1      CACAGCCTTCCCT--CACCGGGGAACCCCTAGCCC-ACAGAGAGAACAGGCGGTGGGGTT
XM_014126406.1      CACAGCCTTCCCT--CACCGGGGAACCCCCAGCCC-GCAGAGAGAACAGGCAGTAGGGTT
XM_014147648.1      CATGCCCTTCCCCACCATTGCGTCGTCCTCTGCCCAGCCCGGGGAGCAGACAGTGGGCCT
XM_014123983.1      CATGCCCTTCCCCACCATTGCGTCGTCCACTGCCCAGCCTGGTGAGCAGACGGTGGGCCT
                    **    *******    **   * *     **    ****  *    * ** *** * ** **   *

XM_014175106.1      GGTGTATAACCAGGAGACAGGCCAGTGGGAGAGAGTTTACCAACCGTCTGCTAGTCCAAG
XM_014126406.1      GGTGTATAATCAGGAGACAGGCCAGTGGGAGAGGGTTTACAGACCAGTCTGCCAGTCCAAG
XM_014147648.1      GGTGTTCAACCAGGAGACGGGCCAGTGGGAGCGCGTCTATCGCCAGGCCGCC--TCCAGC
XM_014123983.1      GGTGTACAACCAGGAGACGGGCCAGTGGGAGCGTGTCTACCGCCAGGCCGCC--TCCAGC
                    *****  ** ******** *********** * ** **     * * * **    ****

XM_014175106.1      TCCAGCG-----GTGAATGTACCACAAGAGGCCTTAAACCAAGAAATGCCTGACGAAAAC
XM_014126406.1      TCCAGCG-----GTGAATGTACCACAAGAGGCCTTAAACCAAGAAATGCCTGACGAAAGC
XM_014147648.1      CGCTCC------GCCGACGCACCACCAGAGGCCTTAAGCCAGGAAATGCCTGTAGATAAC
XM_014123983.1      CGCTCCACCGTTGCCGACGCACCGCCAGAGGCCTTAAGCCAGGAAATGCCTGTGGATAAC
                      *  *      *   * * *** * *********** *** **********  ** * *

XM_014175106.1      CACGATGACGATTACCTGAGAAGGAGGCTTTTGGAGTCATCTCTTATGTCGCAGTCTCGG
XM_014126406.1      CCTGATGACGATTACCTGAGAAGGAGACTTTTGGAGTCATCTCTTATGTCGCAGTCTCGG
XM_014147648.1      CCGGATGAGGACTCTCTGAGAAGAAGGCTGTTGGAGTCCTCCCTCCTCTCTCTCTCCCGC
XM_014123983.1      CCAGACGAGGACTCTCTGAGGAGGAGGCTGTTGGAGTCCTCCCTCCTCTCTCTCTCCCGC
                    *  ** ** ** *  ***** ** ** ** ******** ** **  * ** *  ** **

XM_014175106.1      TATGACATATCTGGCTCCCGGGACCATCCTATTTATCCAGATCCTGCCAGGTTGTCCCCT
XM_014126406.1      TATGACATATCTGGCTCCCGGGACCATCCCATTTATCCAGATCCTGCCAGGTTGTCACCT
XM_014147648.1      TACGACATGGGAGGATCCCGAGACCACCCTATCTACCCAGACCCCGCCAGGTTATCCCCG
XM_014123983.1      TACGACATGGGAGGATCCAGAGACCACCCCATCTACCCAGACCCTGCCAGGTTGTCCCCG
                    ** *****     ** *** * ***** ** ** ** ***** ** ******** ** **

XM_014175106.1      GCTGCATATTATGCCCAGCGGATGATTCAGTACTTATCTCGGCGGGACAGCATTCGCCAA
XM_014126406.1      GCTGCATATTACGCCCAGCGGATGATTCAGTACTTATCTCGGCGGGACAGCATTCGCCAA
XM_014147648.1      GCTGCGTACTATGCCCAGAGGATGATCCAGTACCTGTCAAGGCGGGATAGTATTCGCCAG
XM_014123983.1      GCTGCCTACTATGCGCAGAGAATGATCCAGTACCTGTCGAGGCGGGACAGTATCCGCCAG
                    ***** ** ** ** *** * ***** ****** * **  ******* ** ** *****

XM_014175106.1      CGATCACTGCGCTACCAG---AGCCGACTGCGGCCTCTCTCATCCACCTCAGATAGCCCA
XM_014126406.1      CGATCACTGCGCTACCAG---AGCCGACTGCGGCCTCTCTCATCCACCTCGGATAGCCCA
XM_014147648.1      CGCTCGCTGCGCTACCAGCAGAACCGGCTGAGGACACTCTCCTCCTCGTCGGACAGCCCT
XM_014123983.1      CGCTCGCTGCGCTACCAGCAGAACCGCCTGAGGACCCTCTCCTCCTCGTCGGACAGCCCG
                    ** ** ************   * *** *** ** * ***** *** * ** ** *****

XM_014175106.1      GGAAGCAACTCCTCCCCCTCCGTTGAGAACAACGAGGTGGACTTTGAGGAGTTTGAGGAT
XM_014126406.1      GGAAGCAACTCTTCCCCCTCCATTGAGAACAACGAGGTGGAATTTGAGGAGTTTGAGGAT
XM_014147648.1      GCCGGCAACCCCTCGGCCGCCATTGAGAACAGCGACGTGGACTTTGAGGAGCTAGATGAT
XM_014123983.1      GCCGGCAACCCCTCGGCCGCAATGGAGAACAGCGACGTGGACTTTGAAGAGCTAGATGAC
```

```
                    *   ***** * **  ** *  * ******* *** ***** ***** *** * ** **

XM_014175106.1      AATGGTGATCGCGCCAGACACAGAGCTCCACGCAACGCTCGTATGTCTGCCCCCTCGCTT
XM_014126406.1      AACGGTGATCGCGCCAGACACAGGGCTCCGCGCCACGCTCGTATGTCTGCCCCCTCACTT
XM_014147648.1      AACGGCGACAGGACGAGGCACAGAACACCGCGCAACGCCAGGATGTCTGCTCCTTCGCTG
XM_014123983.1      AACGGAGACAGGACGAGGCATCGAACACCACGCAACGCTAGGATGTCTGCGCCTTCACTG
                    ** ** **  *  * ** **  *  * ** *** ****  * ******** ** ** **

XM_014175106.1      GGCAGATTTGTTCCCCGGAGGTTCCTACTGCCTGAGTATCTGCCATATGCTGGGATCTTC
XM_014126406.1      GGCAGATTTGTTCCCCGGAGGTTCCTACTGCCTGAGTATCTACCATATCCTGGCATCTTC
XM_014147648.1      GGGCGCTTCGTTCCCCGGCGGTTCCTGTTGCCTGAGTATCTGCCCTACGCTGGGATCTTC
XM_014123983.1      GGGCGATTTGTTCCTCGGCGGTTTCTTTTGCCTGAGTACCTGCCCTACGCTGGGATCTTC
                    **  * ** ***** *** **** **  ********** ** ** **  **** ******

XM_014175106.1      CACCATGAGAGGGGCCAGCCTGGCCTGGCCACCCATTCCTCCGTCAACAGGGTCCTAGCA
XM_014126406.1      CACCATGAGAGGGGCCAGCCTGGCCTGGCCACCCATTCCTCTGTCAACAGGGTCCTAGCA
XM_014147648.1      CAC---GAGAGGGGACAGCCTGGCCTTGCCACTCACTCCTCTGTCAACAGGGTCTTAGCA
XM_014123983.1      CAT---GAGAGGGGACAGCCTGGCCTGGCCACCCACTCCTCTGTCAACAGGGTCCTAGCA
                    **    ******** *********** ***** ** ***** ************ *****

XM_014175106.1      GGTGCGGTTATTGGCGACGGTCAGTCGGCTGTGGCCAGTAACATCGCCAACACCACCTAC
XM_014126406.1      GGTGCGGTTATTGGTGACGGTCAGTCAGCTGTGGCTAGTAACATCGCCAACACTACCTAC
XM_014147648.1      GGTGCCTCCATCGGGGACGGCCAGTCGGCGGTGGCTAGTAACATAGCCAACACCACCTAT
XM_014123983.1      GGTGCCTCAATCGGGGACGGCCAGTCGGCGGTGGCCAGCAACATAGCCAACACCACCTAC
                    *****    ** ** ***** ***** ** ***** ** ***** ******** *****

XM_014175106.1      CGCCTCCAGTGGTGGGACTTCACCAAGTTTGACCTACCAGAAATCAGCAACTCCTCTGTC
XM_014126406.1      CGCCTCCAGTGGTGGGACTTCACCAAGTACGACCTACCTGAAATCAGCAACTCCTCTGTC
XM_014147648.1      CGGCTGCAGTGGTGGGACTTCACCAAATTCGATCTGCCTGAGATCAGCAACGCCTCTGTG
XM_014123983.1      CGGCTGCAGTGGTGGGACTTCACCAAGTTCGATCTACCGGAGATCAGCAACGCCTCTGTG
                    ** ** ******************** *  ** ** ** ** ********* *******

XM_014175106.1      AACCTGCTGGTGCAGAACTGCAAGATCTACAATGACGCCAGCTGTGACATCTCTGCAGAC
XM_014126406.1      AATGTGCTGGTGCAGAACTGCAAGATCTACAATGACGCCAGCTGTGACATCTCTGCAGAC
XM_014147648.1      AATGTGCTGGTGCCAAACTGTAAGATATACAACGACGCCAGTTGTGACATCTCAGCGGAT
XM_014123983.1      AATGTGCTGGTTCCAAACTGTAAGATCTACAACGATGCCAGCTGTGACATCTCGGCAGAC
                    **  ******* *  ***** ***** ***** ** ***** *********** ** **

XM_014175106.1      GGGCAGCTCCTGGCCGTGTTCATACCCAGCAGCCAGCGGGGCTTCCCGGATGAGGGCATC
XM_014126406.1      GGGCAGCTCCTGGCTGTGTTCATACCCAGCAGCCAGCGGGGCTTCCCAGACGAGGGCATC
XM_014147648.1      GGGCAGCTCCTGGCCGTGTTCATACCCAGCAGCCAGCGGGGCTTCCCTGACGAGGGCATC
XM_014123983.1      GGGCAGCTCCTGGCAGTGTTTATACCCAGCAGCCAGCGAGGCTTCCCTGACGAGGGCATC
                    ************** ***** ***************** ******** ** *********

XM_014175106.1      CTGGCTATCTACTCTCTGGCGCCTCACAACCTGGGAGAGATGCTCTACTCCAAGAGATTC
XM_014126406.1      CTGGCTATCTACTCTCTGGCGCCTCACAACCTGGGAGAGATGCTCTACTCCAAGAGATTC
XM_014147648.1      CTGGCCGTGTACTCCCTGGCCCCTCACAACCTGGGGGAGATGCTCTACACCAAGAGATTT
XM_014123983.1      CTGGCTGTGTACTCCCTGGCCCCTCACAACCTGGGGGAGGTGCTCTACACCAAAAGATTT
                    *****  * ***** ***** ************** *** ******** **** *****

XM_014175106.1      GGTCCTAATGCCATCTCTGTCAGCCTGTCCCCGATGGGCCACTACGTGATGGTGGGCTTG
XM_014126406.1      GGTCCTAATGCCATTTCTGTCAGCCTGTCCCCAATGGGCCACTACGTGATGGTGGGCTTG
XM_014147648.1      GGTCCCAATGCCATCTCGGTGAGCCTGTCTCCCATGGGCTGCTACGTGATGGTGGGCCTA
XM_014123983.1      GGTCCCAATGCCATCTCGGTGAGCCTGTCTCCCATGGGCTGTTACGTGATGGTGGGCTTG
                    ***** ******** ** ** ******** ** ******   *************** *

XM_014175106.1      GCCTCCCGCAGAATCCTGCTCCATCACACTACAGACCACATGGTAGCGCAAGTGTTACGC
XM_014126406.1      GCCTCTCGCCGGATCCTGCTCCATCACAACACAGACCACATGGTAGCGCAAGTGTTACGC
XM_014147648.1      GCATCTCGCAGGATCCTGCTACATCCCTCCACTGACCACATGGTGGCGCAAGTGTTCCGG
XM_014123983.1      GCCTCCCGCAGGATCCTGCTGCATCCCTCCACTGACCACATGGTGGCGCAAGTGTTCCGG
                    ** ** *** * ******** **** *   ** *********** *********** **

XM_014175106.1      CTGCAGCAGCCCCATGGTGGAGAGACTTCCATGAGGAGAATGTTTGATGTGGTGTACCCC
XM_014126406.1      CTGCAGCAGCCCCATGGTGGAGAGACTTCCATGAGGAGAATGTTTGATGTGGTGTACCCC
XM_014147648.1      CTGCAGCAACCCCACGGCGGCGAGACCTCCATCAGGATGGTGTTTAATGTGGTGTACCCC
XM_014123983.1      CTGCAGCAGCCCCACGGTGGAGAGACCTCCATCAGGATGGTGTTCAACGTGGTCTACCCC
                    ******** ***** ** ** ***** ***** ****   ****  * ***** ******
```

```
XM_014175106.1      ATGGCTTCTGATCAGCGGAGACACGTCAGCATCAACTCGGCCCGCTGGCTCCCAGAACCT
XM_014126406.1      ATGGCCGCTGATCAGCGGAGACACGTCAGCATCAACTCGGCCCGCTGGCTCCCAGAACCT
XM_014147648.1      ATGGCTCCGGACCAGCGGCGGCACGTCAGCATCAATTCAGCCAGATGGCTTCCAGACCCG
XM_014123983.1      ATGGCTCCGGACCAGCGGCGGCATGTCAGCATCAATTCAGCCAGATGGCTTCCAGACCCG
                    *****  * ** ****** * ** *********** ** *** * ***** ***** **

XM_014175106.1      GGCCTGGGGCTTGCATACGGTACCAACAAGGGAGACCTAGTCATCTGCAGACCTGTAGAT
XM_014126406.1      GGCCTGGGTCTTGCCTACGGTACCAACAAGGGTGACCTCGTCATCTGCAGACCTGTAGAT
XM_014147648.1      GGGATGGGCCTGGCCTACGGGACCAACAAAGGGGACTTGGTCATTTGCAGGCCTGTGTTG
XM_014123983.1      GGGATGGGCCTGGCCTACGGGACCAACAAAGGAGACCTGGTCATCTGCAGGCCCGTGTTC
                    **  **** ** ** ***** ******** ** *** * ***** ***** ** **

XM_014175106.1      GCCCAGTCAGATGGAGACGGCCCCGCTGA---GCACAGTGAGCCCATATTTGCTGTCAAC
XM_014126406.1      GCCCAGTCAGACGGAGACGGCCCCGCTGA---GCACAGTGAGCTCGTATTTGCTGTCAAC
XM_014147648.1      TACCGGAGTGACGGAGAGAGCCCGGCCGAAGCGAGCACCGAGCCCATATTCACAGTCAAC
XM_014123983.1      TACCGTAGTGATGGAGAGAGCCCGGCCGAAGCAAGCACAGAGCCCATATTCACAGTCAAC
                      **     ** *****  **** ** **      **  **** * ****  * ******

XM_014175106.1      AACAATCGAGGCAGTGGTACCGGTGGGAGCCGCGATGGAGAGCGGCCAGGGAGCAGCAGG
XM_014126406.1      AACAATCGAGGCGGTGGTACCGGTGAGAGCCACGACGGAGAGCGACCAGGGAGCAGCAGG
XM_014147648.1      AACA---GTGGTGGTGGAACAAGCAGGACCCGCGGTACAGATCGACCAGGGGCCCCCAGT
XM_014123983.1      A------GCGGTAGTGGAACCAGCAGGACCCGCAGTACAGATCGACCAGGGGCCCCCAGT
                    *      * **  **** **  *   ** ** *     *** ** ******  *  ***

XM_014175106.1      -GCCCC--CTTGAGGTCTGAAAGGGACATGGGACTTATGAACGCCATCGGTCTCCAGCCC
XM_014126406.1      -GCCCC--CTTGAGGTCTGAAAGGGACATGGGACTTATGAACGCCATCGGTCTCCAGCCC
XM_014147648.1      CGCTCCAGCTGGAGACTTGACAGAGACATGGGTCTGATGAACGCCATTGGCCTCCAGCCC
XM_014123983.1      CGCTCCAGCTGGAGACTTGACAGAGACATGGGTCTCATGAACGCCATTGGCCTCCAGCCC
                     ** **  ** ***   *** ** ******** ** *********** ** *********

XM_014175106.1      CGTCACCCTACCCCCGTTGTGACCTCACAAGGCACACAGACACCCGTCCTACAGCTTCAG
XM_014126406.1      CGTCACCCTACCCCCGTTGTGACATCACAAGGCACACAGACACCCGTCCTACAGCTTCAG
XM_014147648.1      CGACACCCCGCCCCCTCGGTGACTTCACAGGGCACTCAGACGCCCATCGTGCAGCTGCAG
XM_014123983.1      CGACACCCCGCCCCCTCTGTGACCTCACAGGGCACTCAGACACCCATCGTCCAGCTGCAG
                    ** *****  *****   ***** ***** ***** ***** *** ** * ***** ***

XM_014175106.1      AATGCCGAAACACAGACTGACCGAGAGCCACAGGTTCCCAGCACCTCCCGGGCCTCCCAA
XM_014126406.1      AATGCCGAAACACAGACCGACCGAGATCCACAGGTTCCCAGCACATCCCAGGCCTCCGGA
XM_014147648.1      AACGCCGAGACGCAGACAGAGATGGACCTCTCCGGGCCCAGCACCTCCCACACT------
XM_014123983.1      AATGCCGAGACGCAGACAGAGAGGGACCTCCCCAGGCCCAGCACCTCCCACGCT------
                    ** ***** ** ***** **    ** *        ******** ****   *

XM_014175106.1      GCAACAGACATCAGTGGACACATAAGGGACCTTCATCCAGGCAGCAGCACAGAAATGGTG
XM_014126406.1      GCAACAGACATTGCTGGGCACATAAGGGACCTTCATCCAGGCAGCAGCGCAGAGACG---
XM_014147648.1      GCTGCTTATGTAGAAGCCCGTCCAGAGGTCCCATCCACAAGCCGTGAGACCCAGGGAGGT
XM_014123983.1      GCTGCTTATG---AAGTCCGTCAAGAGCTCCCGTCCACGAGCCGCGAGGCCAAAGGGAGC
                    **  *  *       *  *    *  *  **      *  ** *     *  *

XM_014175106.1      CCTGAGACGCCCCCTCACACACTGCAGGAGGATGGCGACTCAGCCGAGGCCTTCACAGAG
XM_014126406.1      ------ACACCGTCTC----------------TG-----TCAGCCGAGGCCTCCACAGAG
XM_014147648.1      CTGGAGCAGCCGCAAACCAGGCGGACTGAGGCCAGTGCCATGGGGGACTCTTCAACAGAA
XM_014123983.1      CTAGAGCAGCCACAAACCAGCCAGATCGAGGACAGAGCCCTGGGGGACGCCTCGACAGAA
                             **                               *  **  * *  *****

XM_014175106.1      GCCAGCACCACCTTGGCTAACCCAGGGGAATCTCAAGATCAGGGCAGTGGGGAGGATGCC
XM_014126406.1      GCCAGCACCACCTTGGCTAACCCAGGGGAATCTCAAGACCGGGGCAGTGGGGAGGACGCC
XM_014147648.1      GCCGGCACCTCCACAGCCAACACCGGCGAGGCCCCAGAGTATGCCCCGGGAGAGGACGCC
XM_014123983.1      GCCGGCACCTCTACCGCTAACACTGGTGAGGCCCCAGAGTACGCCCCGGGAGAGGACGCC
                    *** ***** *    ** *** * ** **  * * ***    * *   ** ***** ***

XM_014175106.1      GTTTCTCGGCTCCGCAGGCTGATCGCCGAGGGTGGTATGACGGCCGTGGTCCAGAGGGAG
XM_014126406.1      GTTTCTCGGCTCCGCGGGCTGATCCCTGAGGGTGGTACGACGGCCGTGGTCCAGAGGGAG
XM_014147648.1      CTGGCGCGGATCCGCCGGCTCATCGCAGAGGGCGGCATGACGGCGGTGGTCCAGCGGGAG
XM_014123983.1      CTGGCACGGATCCGCCGGCTCATCGCAGAGGGCGGCATGACGGCGGTGGTTCAGCGGGAG
                     *  * *** ***** **** *** * ***** ** * ****** ***** *** *****

XM_014175106.1      CAGAGCACCACCATGGCCTCCATGGGTAGCTTCGGTAACAACATCATCGTTAGCCACCGC
XM_014126406.1      CAGAGCACCACCAT---------GGGCAGCTTCGGTAACAGCGTCATCGTTAGCCACCGC
```

```
XM_014147648.1      CAGAGCACCACCATGGCCTCCATGGGCGGCTTCGGCAACAACATTATCGTCAGCCATCGC
XM_014123983.1      CAGAGCACCACCATGGCCTCCATGGGTGGGTTTGGCAACAACATTATCGTCAGCCACCGC
                    **************         ***  * ** ** **** * * ***** ***** ***

XM_014175106.1      ATCCACCGCGGCTCCCAGACTGGGACAGAGGCT-CAGGATGGGGGCAGAGACACTCAGGG
XM_014126406.1      ---------GGATCCCAGACTGGGACAGAGGCT-CAGGGTGGGGGCAGAGGCACCCAGGG
XM_014147648.1      ATCCACCGCGGCTCCCAGACCCGGACCGGCGCCGCAGCCTGGCTGCCCAG-CAACAGTGA
XM_014123983.1      ATCCACCGCGGCTCCCAGACTGGGGCGGGTGCTGCAGCCAAGCGGCCTAG-CAGCAGTGA
                             ** ********  ** * *  **  ***    *  **  ** **     *

XM_014175106.1      CAGGGGTCAGGGTCTTGGTGGAGGTGGTTGGACGTCCTTC-TCTCCGTCCCACCGCGGCA
XM_014126406.1      CAGGGGTCAGGG---TGGTGGTGATGGTTGGACGTCCCTC-TCTCCGCCCCACCGTGGCA
XM_014147648.1      CCCCAACCTGCC---CGTCCCCGGCCACTCTACAGCCTCCATTTCCACCTCCTCCGCCTG
XM_014123983.1      CCCTATTCTGGC---CGACCCTGGCCCCTCTACAGCCTCCCCTTCCA---------CCTG
                    *      * *      *     *     *  **  **  *   ***

XM_014175106.1      CTCTGTCGTTCACAGAGCCCACCTCCCGTATTCTGACCCACCACAGCATGCAGCTGGGCG
XM_014126406.1      CTCTGTCGTTCACAGAGCCCACCTCCCGTATTCTGACCCACCAGAGCATGCAGCTGGGTG
XM_014147648.1      CCCCCTCACTATCA---CCCAGCCTCAGCCTTCCCAGACCTTGCATC--TCAGCCTGG--
XM_014123983.1      CCCCCTCA---------------------------ACTCCCTGTGTC--TCAGCCTGG--
                    * *  **                            *  *       *   ****  **

XM_014175106.1      AGAGCTCAGGATTCCTGTTAGAGCCCT-CTGGGGCCCATAGCAGGACAGTAACACCTCTC
XM_014126406.1      AAAGCTCAGCCTTCCTGTTAGAGCCCT-CTGAGGCCCATAGCAGGACAGTAACACCTCTC
XM_014147648.1      AGAACTCGTCCGCTCCCACAGAACC------AGGCCCGGTGTTGGG----GCTGCCCTTT
XM_014123983.1      AGCACCCCTCTGCTCCCTCAGAGCCTCGCCCAGGCCAGGTGTGGGG----TATGCCCTCC
                    *   * *       *    *** **       ****    *  **         **

XM_014175106.1      CCCTCCCAGTACATCACAGTCTCTTCTCATCTCAGCCCTCAGGGGCCCATTGGGGTGGAG
XM_014126406.1      CCTTCCCAGTACATCACAGTCTCTTCTCATCTCAGCCCTCAGGGGCCCATTGGGGTGGAG
XM_014147648.1      GTCTCCCA----ACCACAACCCC--CCAGCCTTG--CTTCAG----------GTGCGGGG
XM_014123983.1      ATCTCTCA----ACCACAACCCC--CTGGCCTAG--CCTCAGAGG-------GTGAGGGG
                       ** **    * ****  * *  *    **    * ****          * * ** *

XM_014175106.1      GAGGGGGCCGATGGGACCCCTATGGACACAGAAGATGCGTTTGAGGGTGTGGGACTTGTC
XM_014126406.1      GAGGGGGCCGATGGGACCCCTGTGGACACAGACGATGCGTTTGAGGGTGTGGGACTTGTC
XM_014147648.1      GCCGGGCTCTCAGTGGCC---ATGGACATCGACAACGTGTTTGAAGTAGCCAGCCGAATG
XM_014123983.1      GCCAGTCTCTCAGTGACC---ATGGACATCGACGACATGTTTGAAGGAGCCGGCCGAATG
                    *   *   *   * * **    ******  **  *   ****** *  *   * *   *

XM_014175106.1      GACGATCTAGATCTACACTTGCCCTCTGCTTC---CTCCTCCTCTTCTTCAACCCTGTCC
XM_014126406.1      GATGATCTAGATCCACACTCGCCCTCTGCTTC---CTCCTCCTCTTCTTCAACCCTGTCC
XM_014147648.1      GACAATTCTGAGCCAGGCCCCTCTTCCTCCTTGATCTTCTCCTCCTCTTCCTCCTCCTCT
XM_014123983.1      GACGATTCTGAGCCAGGCCCCTCTTCCTCCTTGCTCTTCTCCTCCTCTTCTTCCT---CT
                    **  **   ** * *  *    * **  * *    ** ****** *****  **    *

XM_014175106.1      CCTGTTGTCTCCCCTCTTGACAACAAC---TTCAGCGCCAGTTACCATGGAGACCCCTAC
XM_014126406.1      CCTGTTGTCTCCCCTCTCAACAACAACAGCTACAGTGCCAGTTACCACGGAGACCCCTAC
XM_014147648.1      CGCAGGTCCTACCCTCCCAGCAGTGGCACAAACGGCAACGATAACAACAGCAGCAGC---
XM_014123983.1      CGCGGCTCTTACCCCTCTGGCACCGGCAGAAATGGCAACAATAACAACAACAACAACGGC
                    *        * ***      **    *       *   *  * ** *      *  *

XM_014175106.1      A--------GTCGATAGATGGTCATGTGGTTTCCATACCCATCTGATCTCTACCTAGAAG
XM_014126406.1      A--------GTCGATAGATGGTCATGTGCTTTCCATACCCATCTGATCTCTACCTAGAAG
XM_014147648.1      ---------AGAGATAGTTACCCAGGTGACCCTTACAGCAGGTAGAGT-----GTGATTT
XM_014123983.1      AGTAACAACAGAGATAGTTACCCAGGTGACCCTTACAGCAGGTAGAGTTCTGTGTGACTT
                                *****  *   **  ***      * * *     **        *

XM_014175106.1      ACTCTCCTCCTTCTAGCTTTATTACATTGACTACT-GGTTTTTCTGGAGGCTTTCAAAAC
XM_014126406.1      ACTCTCCTCCGTCTAGCCTTAATACATTGACTACT-GTTTATTCTGGAGGCTTCCAAT-C
XM_014147648.1      GACCATTATTGCCCAGGAGTGGGAGAGAATCCCCCAGAGCAGACAGTGGGTGACTGATGC
XM_014123983.1      GGCCCTTATTGCCCAGGAGAGGGAGAGAATCCCTCAGAGGAGATAGTGGGTGACTG----
                       *        * **       * *    *     *        *  **

XM_014175106.1      C--CGACAATGAAAT-AGGCACTTAAAACTTCATCCCACTCCTCCATTGCCTGTTCAGTT
XM_014126406.1      C--CAACAATGAAATGAGGCACTTAAAACTTCATCCTACTCCTCCATTGCATGAACTGTT
XM_014147648.1      TAGTGACGATGCTTTGGGCCGAGAGAAGGTGTATCAGGAACCCTAATGGAACTCAGTGTG
XM_014123983.1      -----------CTTTGGGTCGACAGATGGTGTATCACGAACCCTAA---AGCCGAGTGTA
```

```
                                  *  * *     *   *  ***     **   *            **

XM_014175106.1      -TTCTATCCAATAGTAGGCTGGAT----GAGACCTATCTTCAACCTAGGATTAGGCTCGG
XM_014126406.1      -TTCTATCCAATAGTAGGATGGAT----GAGACCTATCTTCAACCTAGGATGAGGCTTGG
XM_014147648.1      GCCCTGTCTAACAGGTGCGCTTCTCCTCAAAGCCTTATCTCACAATAAATGGGTTCTCAG
XM_014123983.1      -CACTACACGATTTTTGGCCCTC-------------------------------------
                       **     *     *

XM_014175106.1      TCTCTTTCTTGGATGTGGTGTTCCTTTGCGTGCCCTGAC--CACTTTCAGACTTTTGTCA
XM_014126406.1      TCTCTTTCTTGGATGTGGTGTTCCTTTGCGTGCCCTGACTTCAATTTGAGACTTGTGTCA
XM_014147648.1      ACTCTGCGTGGTCTCCAGCCCCAAACAGAAGGGACATGAGCCCCCCCCCCCCCCCCCCCC
XM_014123983.1      ------------------------------------------------------------


XM_014175106.1      TTGCCTAATTGAGAGCAAGAATCCAAAGCTACAGTTTGACCATTCCATCGGAGAGAGGTG
XM_014126406.1      TTGCCCAATTGAGTGCAAGAATCCAAAGCTACGGTTTGACCATTCCATCGGAGGGAGGTG
XM_014147648.1      CCATCAGCTTCAGTGGCATTGTGACCCCCTCTGCATTGGTGTGCACTTACATTTGCACTT
XM_014123983.1      ------------------------------------------------------------


XM_014175106.1      GACCTGAATGACTCTTTTACAATCAGGGTT-CAGATGGGTGGTTTTCGAATGCCACCTGG
XM_014126406.1      GACCTGAATGACTCTTTTACAATCAGGGTTTCAGATCGGTGGTTTTCAAATGCCATCAAG
XM_014147648.1      AAAATGGACCTTAACTGTATGATCATTCCATGTCAGAAAGGGCTTTTACTTGAATTGTGA
XM_014123983.1      ------------------------------------------------------------


XM_014175106.1      GACCAAATTCCATGATCAAATTGCAACGTTCTTCAAAACGTGTTAAGACTCTGCCCCTTG
XM_014126406.1      GACCAAATTCCATGATCAAATTGCAACTTTCTTCAAAACGTGTTAAGACTCT------TG
XM_014147648.1      GTTTTTTTCAGTCCTTCTCTTCACATAGGCGTGACCGATAGACAAGGTTACTCCATTCAA
XM_014123983.1      ------------------------------------------------------------


XM_014175106.1      CTCACTGTTAGCAAGATGCTGGTAT----AGTAGAAGCACATTTTAT-------------
XM_014126406.1      ATCACTATTAGCATGAAGCTGATATGCTGAGTAGAAGCACATTTTATTAACCAATCACAT
XM_014147648.1      AGTGCCATGTAAGGGTAATTGTAAAGAACAAAAAAAAAACAGAGTGTCATGTTCTGGTGC
XM_014123983.1      ------------------------------------------------------------


XM_014175106.1      ---------------------------GATTCTGT--------------------CTATA
XM_014126406.1      TGGTACAGTTTGATTGTGAAACTGGATGATTCTGTATCATTTTTATTTTTTATGTCTATG
XM_014147648.1      CATGTGATTGAATTTGACATGAAATAGTGTGTGTAGGACGTGTTATAGGACGTGCATTTG
XM_014123983.1      ------------------------------------------------------------


XM_014175106.1      CGTTATATT-TATTGTGTGTGAAGGAG--GATGTTCTATGTCACCCTTGGTAGTTACAAA
XM_014126406.1      AATTATATTCTATTGTGTGAGAGAGAGAGGATGTTCTATGTGACCCTTTGTGGTTACAAA
XM_014147648.1      TAATGTGAT-GATAATGCATAGCGGGAAAATAGTCCTATCGTTGTATGAATCATGATTTG
XM_014123983.1      ------------------------------------------------------------


XM_014175106.1      ATGTTTCATTCT--------AGTCGTCATGGTTGATTAGTGGTTTGCAAAGATGTAGTT-
XM_014126406.1      ACTTTTCATTCTTCCGTTATAGTCGTCATGGTTGATTAGTGTTTCGCAAAGATGTAGCT-
XM_014147648.1      ATGTGTGTGGCATG-ACTAAAGCCATGCCTTTCAAGAGAGAATCTGCAGCTGCCTGCTTT
XM_014123983.1      ------------------------------------------------------------


XM_014175106.1      -GACCCAAGATAACTAT-GAATCTCTTGTACTACATAGGAGAGCAGCATTACATTACAGA
XM_014126406.1      -AACCCAAGATAACTTTTGAATCTCTTGTACTACATAGGAGAGCAGCGTTACATTACAGA
XM_014147648.1      CATTATAGATTGACTATGTCAGAGGGGTCCCCCCCAAAAGGATCATGGTCATATTACCCA
XM_014123983.1      ------------------------------------------------------------


XM_014175106.1      GTAGAATTTAGGG----------------AGACCA------ATTCCACAATATGCTACT
XM_014126406.1      GTAGAATTTAGGGTTGGGCAATATTTCGGGAGACCA------ATTCCACCATATGCTACT
XM_014147648.1      TGAGTCCCCAAGTGTACAAAGCATCTGATTCGATGGTACAAGACTATGCCCCTTATTGCT
XM_014123983.1      ------------------------------------------------------------
```

```
XM_014175106.1      CCAAATATGAGCGATGTTCGTTATTATCGCTGT-------TAATGACAAAATAATTCCAG
XM_014126406.1      CCAAATATGGGCGATATTCGATATTATCGCTGTGCGCTGTTAATGACAAAATAATACGCG
XM_014147648.1      TCATGTTCAAACAGGATACAGTGGACTTATACAAAATGGTGGATGTGATGTTTCAATGTC
XM_014123983.1      ------------------------------------------------------------

XM_014175106.1      ACTTATTTTTTTGCTGATTTTAT-GATATCATAATCACCTTCTCATTTAGACTTCTTTTG
XM_014126406.1      ACTCCTTTCTTTGCTA--TTTAT-GATCTCATAACCTCCTTCTCATTTAGACTTCTTTTC
XM_014147648.1      TAGTCAATCATTTCTGACTGTATTGATACTGTAGTATTGTTCTACTGCATGTTTTCAATA
XM_014123983.1      ------------------------------------------------------------

XM_014175106.1      AA--CACATTGATACTGTCTAACTGATAAA-CAGCCCGTTTTTGAT----TTGTAAAGTT
XM_014126406.1      AA--CATATTGATACTGTCTAACTGATAAAACTGCACGGTTTTGAC----TTGTAAAGTT
XM_014147648.1      ATGCTGCATCGTTTCTGAAGAAAAAAGGGAACGCCATCGGTACAAAAGTATTGTGAAATA
XM_014123983.1      ------------------------------------------------------------

XM_014175106.1      CAAATAGAGTATTGTCTAGTGCA----GGTATTCCCATACTGGGGGTACCCCTGGGGGGT
XM_014126406.1      CAAATAGAGTATTGTCTAGTGCGTCACAATAGCTCGACCATATAGTTGATGACAAATATT
XM_014147648.1      TGGACAAATCGGCATTGAGGGAGGGAGGGAAAGGGTGTCGGGGGAACTAACTCGTACACC
XM_014123983.1      ------------------------------------------------------------

XM_014175106.1      ACACACAATGCCGTCGGGGGTACGCCAAATCATTTCT---TTATTTTA--AAACAGTACA
XM_014126406.1      GCCGATCCTCGAGTTTGTCATATGTTAAGCCAAACCTA-GTAGCATTAC-AAGCCGTTGC
XM_014147648.1      TGTTTGGGGAGAGTTAGGCAGCTCTCTAGACATGCTTGTGTCACGCTAAGAGGCAGCACC
XM_014123983.1      ------------------------------------------------------------

XM_014175106.1      TTTTATATTTTCCAATGGGGCTGTA-CATTTGGGTGAGGTTTTTTTCTCACCT-------
XM_014126406.1      TACCAGATAATACCATTACTCTTTC-CTCCCCAGTGTAATGCCTGAATCTGGTGTACAGT
XM_014147648.1      GTCATTCCTCTTCTGTTGCACTACAGTGACTGGGTTTGGTGCCAAGAGGCGTGCAGTGAG
XM_014123983.1      ------------------------------------------------------------

XM_014175106.1      ------------------------------------------------------------
XM_014126406.1      ATGCGGTCGGATGTGTGTGTGACAATGGTTCATCTGTACCCAGAAAAGAAAACAAACCGC
XM_014147648.1      GCCTGGGTCAGGATTTGTTTACCATCTGGTCCCTGGTCACCCATCCCACAAACATCTACC
XM_014123983.1      ------------------------------------------------------------

XM_014175106.1      ------------------------------------------------------------
XM_014126406.1      ATGACTTATGTATTTCAAAGGTTGATTTAATAATAAC----AGGGTACAGTACACATGTA
XM_014147648.1      ATCCCCTCCTAAATGTGTGATGTAGACCAAGACCAGACCCAGAGCCCCCTGTCGCCTGCA
XM_014123983.1      ------------------------------------------------------------

XM_014175106.1      ------------------------------------------------------------
XM_014126406.1      CTAGTGGCATGTAGATCATGAGCCCTAGATCCGCAGCCTCGTAACTTCCAAACTTGTTTT
XM_014147648.1      CTCACCACTGGTGCTCCAGGGACACGTGAGAGCCGAGAAGTTACCACTAGGATTCAGTGA
XM_014123983.1      ------------------------------------------------------------

XM_014175106.1      ------------------------------------------------------------
XM_014126406.1      GAAACACCGAAATGCTGTGCCAAGTATTTGAAGTGAAGCATTTTGACAATAGTGCATCTC
XM_014147648.1      AACAAACCTGACTGTTGACTTGTTACGCCCAGTGACCCTTTCCACAAAGACTCTGAGGAG
XM_014123983.1      ------------------------------------------------------------

XM_014175106.1      ------------------------------------------------------------
XM_014126406.1      TTCCACGCTGTCAAACTGTCACCCCATGTCTTGTGATGGTTGTCTTTTATACAGTAGGAT
XM_014147648.1      GAGCGAGCAGGGGGCTAGTTGGATTGTCTCAGGTGTAGCTGTCTGTCAACAGGGGGAGGG
XM_014123983.1      ------------------------------------------------------------

XM_014175106.1      ------------------------------------------------------------
XM_014126406.1      TCCTTCTCTAGATCCACTGTGGTATGTTGAGCCCCGTCATCTAAGACTAAATGGAATTTG
```

```
XM_014147648.1      TCTTCACTGAGCTTCTTTACAGTTAAATCCCCAGCACAAACACACCCAGTGCTTAGGAAG
XM_014123983.1      ------------------------------------------------------------

XM_014175106.1      ------------------------------------------------------------
XM_014126406.1      AAAAGGAGATGCATCCAGAAGGCTCTCTTTAAAATGGCCAGTTTGCCTCCCACAAGCCGC
XM_014147648.1      CACTGAGGTGGAGCTGTTCAGTGTGTGTATGCTTGTCTCTGTGTATGCGTGTGCAGTTGT
XM_014123983.1      ------------------------------------------------------------

XM_014175106.1      ------------------------------------------------------------
XM_014126406.1      CCCGGTGTCTTGAGGGTTGTGTTGTTGTGACAGAGAGGCAGTTGGAGGTGCACAGGGGTG
XM_014147648.1      AAGTGTGTGTGGAGGTGGTGTATGTTTATTGGCTCCGCAAAATGTAGGCATTCTGCAGCG
XM_014123983.1      ------------------------------------------------------------

XM_014175106.1      ------------------------------------------------------------
XM_014126406.1      ATGATCGCATTCCGAGTCACCACAGGGGCAAAGCTCTCTAGGTTGAATGGACT-------
XM_014147648.1      GAAAAACAGGAGAGAAAGTTTGCCTTTGGCCTATAACCGTGGGTGGGTGGGCCAGAAGGC
XM_014123983.1      ------------------------------------------------------------

XM_014175106.1      ------------------------------------------------------------
XM_014126406.1      TTGTGTGCTTCAAACAGATTTGTCAGTTCTTAATTCTTTTTAAAACTGGTTTGGCCATTA
XM_014147648.1      TTGTATATGTAGCATCTCTTAGTCAAAGTTTCTTTGCCCTAATGGTGTGGTTTGTCACTT
XM_014123983.1      ------------------------------------------------------------

XM_014175106.1      ------------------------------------------------------------
XM_014126406.1      GCAGTAATCTGCAAAAAATAATACATTTTGTTTATTGTTTTTCCTATCAAGGTGGTGGGT
XM_014147648.1      GGGGTACGTCGGGGTGAGAGAGCTTTCGATTATAATTTCAATCAGTCCCGTGCTGAACCA
XM_014123983.1      ------------------------------------------------------------

XM_014175106.1      ------------------------------------------------------------
XM_014126406.1      TTTGACAGTGAAGGAAACTGTGTTGAATTAATGAAT------TTGGAAGTGCTTACTGCA
XM_014147648.1      GGAAATGGATAAAAAAATCAACTTGAATTATCGATTACCATTTAGATCGAGTTTTGTACT
XM_014123983.1      ------------------------------------------------------------

XM_014175106.1      ------------------------------------------------------------
XM_014126406.1      GCTCCATGGTGAATGGGTTACTTTTTTGATATTTGGATGTGAAAAGTTACGCATTATGAG
XM_014147648.1      GTAATGTCTTCAGGGGTGTGCCTGGCCTATTGTGACAGGCAGAGTGTCCTTAGCATATAA
XM_014123983.1      ------------------------------------------------------------

XM_014175106.1      ------------------------------------------------------------
XM_014126406.1      CAGGTTTTTGTCTCTCAGCCACTGTTGGTCCAGCCAACTTGCACTGCTTATATCTGTCTC
XM_014147648.1      TTTCACTAAAGCCCCCCCCGACCACGTCGGGTGCTTACAGACAAGAAAAATAAATGCCCT
XM_014123983.1      ------------------------------------------------------------

XM_014175106.1      ------------------------------------------------------------
XM_014126406.1      TCTCTCAAAAGTATATTGTGATTGTATCTGTTGAGTTCATGTTTTAGAAAGATATTGGTT
XM_014147648.1      GCACACTATAACTTTTATTTTTAAAACCCATAATGCTAAGCAATTTACATTTTAATCGCT
XM_014123983.1      ------------------------------------------------------------

XM_014175106.1      ------------------------------------------------------------
XM_014126406.1      ATAGAAGAAGATATGCCAACTTAAGCAATAACGTAAGGTTGATCATAATCTTTTTTCATA
XM_014147648.1      TTGTAGGGTTATTATATTGAGAGAGAGAGAGAGAGAGAGAATTGGTGGTTTTTTGAGCTG
XM_014123983.1      ------------------------------------------------------------

XM_014175106.1      ------------------------------------------------------------
XM_014126406.1      TACATTTACCAGAGTTGCATTAGTCATGTGACCCTGCAGCAGCACTGTCTATCTCTGTTG
XM_014147648.1      TGCAGCGACTCCAAGCAAGCTGTTTACTGCATGTATGAAAACTGTCTTACTGATTAGAAG
XM_014123983.1      ------------------------------------------------------------
```

```
XM_014175106.1      ------------------------------------------------------------
XM_014126406.1      TTCAGATCACTTCTAGGAGAGCAATGCACCATTTGTCGGTTCATATGCTGATCTGACTAC
XM_014147648.1      AAAGTCAAATTGCCTGGCAGGTGAAAAAGAAACAAACTATCTGAATGGTCCTCCTGGCTT
XM_014123983.1      ------------------------------------------------------------

XM_014175106.1      ------------------------------------------------------------
XM_014126406.1      AAACAACTTCAGGAAGATTGTCAAAAAGATAAATATATTATGTTCATTGTTTATCCGATT
XM_014147648.1      TACTTGATTAATGAAGATTTAAAAAAATGTTTGTCGGCTGTTCATGTGTGCTTTGTGTGA
XM_014123983.1      ------------------------------------------------------------

XM_014175106.1      ------------------------------------------------------------
XM_014126406.1      GTGAAATTGTCTCCCCAGTGTTTTCCTATACTGTATGACTGACCACTTACTTGTACTGTG
XM_014147648.1      AAAAGCACTGTCTGCCATATGTCCCTCACCCTGAATGATACACATACTGTAGGTCCACAA
XM_014123983.1      ------------------------------------------------------------

XM_014175106.1      ------------------------------------------------------------
XM_014126406.1      CCGATCTTCATTTTA---ATGTCCTCAATTAAGGGGTCCCATTATGGCTTGTAATGACTC
XM_014147648.1      CAACACGTTTAACAATGCAAATCAATGGTATACAAACTCTCACCTTATATTACAAAACTT
XM_014123983.1      ------------------------------------------------------------

XM_014175106.1      ------------------------------------------------------------
XM_014126406.1      CTGTAAGGTTATGGGAAATACTAGATTACCTCTCATAGCGTCGGACTCTGAGTGAAATTA
XM_014147648.1      CTATATCAAACTATCAAGTTTGTTCACTTCTTTGGCTTTTCTGTGATGTAATTAAAAAGA
XM_014123983.1      ------------------------------------------------------------

XM_014175106.1      ------------------------------------------------------------
XM_014126406.1      GATTTGGACAAATATATGCTTGTTATTCAAGCATTAATGTGTTTCATGTTAGCGTATGAT
XM_014147648.1      GATTTGTTGACATAAAGATGAAAAATGTAACAATTATGTTCTGTGAAGAGATGGCAATCT
XM_014123983.1      ------------------------------------------------------------

XM_014175106.1      ------------------------------------------------------------
XM_014126406.1      CCATGAGGGACAATACCATTCTGATTTTA-TACAAAAATCATCTGTTTACAATCGACCCT
XM_014147648.1      TATTGGTTCTTAATGGAAAGCTACAGCTACTGTAAATTGTATACAGTTGCTATGACCTGC
XM_014123983.1      ------------------------------------------------------------

XM_014175106.1      ------------------------------------------------------------
XM_014126406.1      TGGAAAGAGCAGGAAGACCGGAGTTTCGTTTACAGTTCTTGGGCTGAATATCAGAGTGAT
XM_014147648.1      GGTATTGAACACCTTAAAGGCCCAAAGCAGCTGTTTTATTACCCGTTTTTGGGTAACAAT
XM_014123983.1      ------------------------------------------------------------

XM_014175106.1      ------------------------------------------------------------
XM_014126406.1      CTCGGTAGGAGTAGAAAGGGATGTAATGGGAAAGTGTTTGCGACAGACAAACCAAGAGAC
XM_014147648.1      TTTGTACATTACTGTAATAGATTTCCATTAAAAAGGGCAAAAATAGCTTTATAGCAAAAA
XM_014123983.1      ------------------------------------------------------------

XM_014175106.1      ------------------------------------------------------------
XM_014126406.1      CCATCAGGCATCAGAATGGTTCAGGCTAACCCAGATCTGTCAGACTCTCTGCTCCTGAAA
XM_014147648.1      AATATTTCTCAAGCAAGAATTTTGCAAGGACTGTCTGGGAATGGTGGAAGGGGACAACTG
XM_014123983.1      ------------------------------------------------------------

XM_014175106.1      ------------------------------------------------------------
XM_014126406.1      AGACCCCCCTCCCCTTTGGAAAGTGGAGCAGACAGCTGACACAGTTTTCTTTTGTTTCCC
XM_014147648.1      AAAACCTGATGTTATTGGCAGAGAGGTTTGAAACTTTTTTTAAAATTGGTATATTAAAGC
XM_014123983.1      ------------------------------------------------------------
```

```
XM_014175106.1      ------------------------------------------------------------
XM_014126406.1      CCCTTTTTTATTTCTTTTTGAGCACCTTCATCTCGTGTTTTTCTTGTTTTTGGTTTTTAA
XM_014147648.1      TGTTAGGGCGACCCAACTAAATTCACATAGAAATGTGAGTTATAGATCTGTCTTTCTCAT
XM_014123983.1      ------------------------------------------------------------

XM_014175106.1      ------------------------------------------------------------
XM_014126406.1      CGTGTGGAATTGACTGTTTTCTTTCACCTGTTTTCTATTGTATTTGAAGAGGATGTGATT
XM_014147648.1      TTAAAGCAAGTCTACGAAGCGGTATATCTGTTCTATGTGTTCTATTTTTATGCTTCCTGG
XM_014123983.1      ------------------------------------------------------------

XM_014175106.1      ------------------------------------------------------------
XM_014126406.1      GACTGCATTCTAGTGTCCTGTTGGTCAGGAGGGTTCATGGAAAGGATGTGTTGTAATCAG
XM_014147648.1      GTCTTTTACTTTCGGTTTTGTACACCAGCTTCAAACAGATGAAAATACAGCATTTCTGGT
XM_014123983.1      ------------------------------------------------------------

XM_014175106.1      ------------------------------------------------------------
XM_014126406.1      AAGGCAGGAGCTTACAGTGGCTTCGTGGTGACTGGTGGGTGAGATGGAGAGAGAAATGGA
XM_014147648.1      TATTGAAATATATTTACAGTGGTTTATATTGTACAATGATTCTCTACACTATGCATTGCT
XM_014123983.1      ------------------------------------------------------------

XM_014175106.1      ------------------------------------------------------------
XM_014126406.1      ATTGGAGCAGGAGAACTGAACAACTGATCCCTGTGGGCTCCAGTCCTGCTGGGCTTGAAG
XM_014147648.1      TGTTTTTTCACATACATTGATATGCCACACTGATATGGCAAACTATTAGAATTTTTACAA
XM_014123983.1      ------------------------------------------------------------

XM_014175106.1      ------------------------------------------------------------
XM_014126406.1      TGGGAGAAAAACAGTGAGCGAAACAGCAGCGTTGGATTTGGTCACCTTGATGCTCATACA
XM_014147648.1      CCAGGAAATGGCACAGCG----ATTTCTGCATCTTGCACCTTTAACCAATTTACTGCATG
XM_014123983.1      ------------------------------------------------------------

XM_014175106.1      ------------------------------------------------------------
XM_014126406.1      GCATTGCGTTAGATGATCTCATCTGTTTCAGCTTTCACCTATCTGTGCTCTTTATTACTC
XM_014147648.1      GCGATGTCACCATTGAAAGTCTAAACTCCTGCCCATGCAAACCTGCTCATTAGAAGGTCC
XM_014123983.1      ------------------------------------------------------------

XM_014175106.1      ------------------------------------------------------------
XM_014126406.1      AGTTCCACTAGCGGTTCTGGAGGGGGAA-ATAACAATTTTGGACCCCCTTGTGGCCCCCC
XM_014147648.1      TGTGTAGATTGTGTTTTCAACCAGCAACTATCAAGAAATAACACTGGTACATTTTTTTTT
XM_014123983.1      ------------------------------------------------------------

XM_014175106.1      ------------------------------------------------------------
XM_014126406.1      TAAATGTGGAGTATGAAATAATTTGTACCTAACAAATTTTTTGCTGTCATTCTTTTTTTA
XM_014147648.1      TTAATACTTTTCCTGTGTTAGTTTTACTATATGATACAAAACACAGGAAAAATTGAATTT
XM_014123983.1      ------------------------------------------------------------

XM_014175106.1      ------------------------------------------------------------
XM_014126406.1      CATCCGTTATTAGACGGTGGCAACGATGATGATTATGAACATGGTCTTTTGCCTGCTAAT
XM_014147648.1      TGATGGCACTGGGCCTTAAAGTAACCAAATGTCATCTATACGGTTATTGTATGCATTACC
XM_014123983.1      ------------------------------------------------------------

XM_014175106.1      ------------------------------------------------------------
XM_014126406.1      GCCTGCAATGCAGTGAAGAAAACAATATGACA-ACAATGTCTAATGTAACTGGCCCCTCT
XM_014147648.1      AGCAGTGAACAAATCCTATTCTTAAGGTGAAATATAATTGGTTTTCTACATCTCATATAT
XM_014123983.1      ------------------------------------------------------------

XM_014175106.1      ------------------------------------------------------------
XM_014126406.1      AACAGTACAACTGGCCCCAGCTTGCCCCCCCAGTTGAAATGGTCTAGAACCGCCACTGCT
```

```
XM_014147648.1      GCAAATAACAGTGGTTTGAGTTCTGTGGAATGGAATAAATTAAAGTGTATTTAACATAGT
XM_014123983.1      ------------------------------------------------------------

XM_014175106.1      ------------------------------------------------------------
XM_014126406.1      CAGTTCACCAACATACAATAATCAGCAGCGTGTGTATCAATTCAAAATTCAATCCAGACC
XM_014147648.1      GGTAGTATAAACTAATTGGATACATGACACTGCATTTAGACACTAATGCTTTGTCAAACA
XM_014123983.1      ------------------------------------------------------------

XM_014175106.1      ------------------------------------------------------------
XM_014126406.1      ATAGTTAAATAGTGGAACTACAGTATATAGAGTAGAAATCTCCACTTACAGTTCTCCACA
XM_014147648.1      A--ATCATACTCCAGACTCCCAATATACATAGGTGTTTTTGAATGTTTAGTATAAGTTGG
XM_014123983.1      ------------------------------------------------------------

XM_014175106.1      ------------------------------------------------------------
XM_014126406.1      GCCCAGGGTGTTTTGCGACTACCCCTGTTTTTAGTTTCTGTGCAATTAAGGGCAATATGT
XM_014147648.1      GAATACTGCTGATTCATCTTAACAAGTGGAGTTGGCCCTCTGTACTTGTGCTTGGCAGGT
XM_014123983.1      ------------------------------------------------------------

XM_014175106.1      ------------------------------------------------------------
XM_014126406.1      AATCACATTACATACTCAGCGTATTTAGTTATAATGCTTTACACTGTTCACAGATCATCA
XM_014147648.1      AATTAATTTAATTAATAGAGATCCAAGAATGGATCCTTGTGGAACACCACATTTGATTTC
XM_014123983.1      ------------------------------------------------------------

XM_014175106.1      ------------------------------------------------------------
XM_014126406.1      CAAAATCAGTGTGGTCAAGACATTACACTGTCATATGTTCACACAGGTGATATTCACATG
XM_014147648.1      AAGAGGGGGGAGTTTATTCCTTTTAATGTGACAGATTGCATTCTTCCTTTTCCTTTTAAG
XM_014123983.1      ------------------------------------------------------------

XM_014175106.1      ------------------------------------------------------------
XM_014126406.1      TTTGTGCACCAGAATAAAAATGTCTGTGACCTTCTGGATATGATTGAAATTTGGATCAAA
XM_014147648.1      AATGCAGATAATGGTCCCCATGTACTTATTTGGTTTTATATTATGAATGTCATGTTTTAC
XM_014123983.1      ------------------------------------------------------------

XM_014175106.1      ------------------------------------------------------------
XM_014126406.1      TCTGTTTAGGAGTTTTTTAAATATATTCAAGTCTGAAATGTGTTTATAAATTGAGGATAA
XM_014147648.1      ACTCCATATTCTTTGTTAAGGCACTTGTGGGGACACCTGTGGTATGCAGTATGTGTGAAA
XM_014123983.1      ------------------------------------------------------------

XM_014175106.1      ------------------------------------------------------------
XM_014126406.1      TGAGC---TTCCTATACAAATACTTTCAGGGAAATTCTAAAGGTCATGAGTGTATTTGTT
XM_014147648.1      CATACGGATACAAACACAGTTTTTCCCACTGATACAATCTATTTTGCTTGACAGTGATCA
XM_014123983.1      ------------------------------------------------------------

XM_014175106.1      ------------------------------------------------------------
XM_014126406.1      CTGTTGTCGCTGACTCACCATAGTGTCTTTTTCTCACACTTATCAATATGTGATCTCCTG
XM_014147648.1      CAGCATTGTCCCAATCATGTTTAATGTCATCCTTGGTTTTTATGAATTTATATCTTTCAC
XM_014123983.1      ------------------------------------------------------------

XM_014175106.1      ------------------------------------------------------------
XM_014126406.1      AGTGTACGTGAACTCAACAAGGAAGTGTCTTCAGCGTGCATGTTTTTTGTAGGAGATTGT
XM_014147648.1      TACTTTGCCCCTTTAGGGCCACCACATTTCACAGTCTCTGAGCTGTTTGTGAGGCTTATT
XM_014123983.1      ------------------------------------------------------------

XM_014175106.1      ------------------------------------------------------------
XM_014126406.1      GATTTGAAGGGTTTTCTATCTACTTTAAAGGGTAAAATGCAGTATATTACAGTAGTCAAT
XM_014147648.1      TTGTTGTGATATTTTTTAAACCATGTGAGGCAGAGTCTTTGGCAAAATGGTTTAGTTCAT
XM_014123983.1      ------------------------------------------------------------
```

```
XM_014175106.1      ------------------------------------------------------------
XM_014126406.1      GTGCAACTAAGTGCTGTTGGAACACCAAAGATCCTAGAAAATATATGTTGAACATAAACA
XM_014147648.1      TTGAGGGAGAGCGGTTGAGGTTTTGCTCAGTGCCTGTATGGTTCACACTAGTTACTGCCA
XM_014123983.1      ------------------------------------------------------------

XM_014175106.1      ------------------------------------------------------------
XM_014126406.1      AATAAAAATGTAA-----------------------------------------------
XM_014147648.1      CTACTTATTCTAAATCTACTAAAACTCTTGGCGCTAGTATGTAGCCAAACAGTCACTTGT
XM_014123983.1      ------------------------------------------------------------

XM_014175106.1      ------------------------------------------------------------
XM_014126406.1      ------------------------------------------------------------
XM_014147648.1      TGATTATACTAATCAATTACAAATTATTGGTCTTGTTGCTTCCTTTGTTAGTGTACAATA
XM_014123983.1      ------------------------------------------------------------

XM_014175106.1      ------------------------------------------------------------
XM_014126406.1      ------------------------------------------------------------
XM_014147648.1      GTCTCTGGTCTAGCCTCATGCTATTCTTCCTATGTTTGGAAGGTTGATTTTATATAGATT
XM_014123983.1      ------------------------------------------------------------

XM_014175106.1      ------------------------------------------------------------
XM_014126406.1      ------------------------------------------------------------
XM_014147648.1      TGAATGTAGTGTATTTTTGCCCAACACATTTCTGTTATAAATTACACCTGTGGTGCTTCA
XM_014123983.1      ------------------------------------------------------------

XM_014175106.1      ------------------------------------------------------------
XM_014126406.1      ------------------------------------------------------------
XM_014147648.1      GTCAGCTACTTGTTCAACTGCTTTCACAGAAGATCTATGCTGCGTATTAAAACTAGGAAC
XM_014123983.1      ------------------------------------------------------------

XM_014175106.1      ------------------------------------------------------------
XM_014126406.1      ------------------------------------------------------------
XM_014147648.1      AAACTAGGGCTGTTTGAAAAAGCAGTATCACACATGACTTCAGTATGAAATTGTCCAGGG
XM_014123983.1      ------------------------------------------------------------

XM_014175106.1      ------------------------------------------------------------
XM_014126406.1      ------------------------------------------------------------
XM_014147648.1      AGCTAATGTTTAGGGTGTGTTGCTGTGTCACAATACTTTAAAAACATACTTTCCTTATCC
XM_014123983.1      ------------------------------------------------------------

XM_014175106.1      ------------------------------------------------------------
XM_014126406.1      ------------------------------------------------------------
XM_014147648.1      TCTTTCATGACCACTGACCAGTGAAAGGACTCAATAGGTTTTCACTATACTGCTGTGCTC
XM_014123983.1      ------------------------------------------------------------

XM_014175106.1      ------------------------------------------------------------
XM_014126406.1      ------------------------------------------------------------
XM_014147648.1      TCATCTATCGAGTCCTCAGGTCAGTAGTGAAGAAAAGGATACCAGGTAAGATATTTACAG
XM_014123983.1      ------------------------------------------------------------

XM_014175106.1      ------------------------------------------------------------
XM_014126406.1      ------------------------------------------------------------
XM_014147648.1      TATGTGTATGACTTGGGATATAGTCTTGACTGCTATGTCTCTTCCCCCTAAACTGTTATC
XM_014123983.1      ------------------------------------------------------------
```

```
XM_014175106.1      ------------------------------------------------------------
XM_014126406.1      ------------------------------------------------------------
XM_014147648.1      AATGGTCTCACATGTTTTAGAGTGCTGATGTGATGAAGATTTTCTGTTCTTTTAAAATAA
XM_014123983.1      ------------------------------------------------------------

XM_014175106.1      ------------------------------------------------------------
XM_014126406.1      ------------------------------------------------------------
XM_014147648.1      ATTAAGATTATAGTAAAAATCCAACAAGTTCTATATCATATATAGGAACACCAAAGATCC
XM_014123983.1      ------------------------------------------------------------

XM_014175106.1      ------------------------------------------------------------
XM_014126406.1      ------------------------------------------------------------
XM_014147648.1      TATGTAATGTTGACAATGAAAAAAGGCAAATAAACAATAAACTTCACAAAGGTGTTATTA
XM_014123983.1      ------------------------------------------------------------

XM_014175106.1      -
XM_014126406.1      -
XM_014147648.1      A
XM_014123983.1      -
```

CLUSTAL 2.1 Multiple Sequence Alignments for *col2a1a*

Sequence type explicitly set to DNA
Sequence format is Pearson
Sequence 1: XM_014134054.1 (*col2a1aa*) 5771 bp
Sequence 2: XM_014168236.1 (*col2a1ab*) 6936 bp
Sequence 3: XM_014135018.1 (*col2a1ac*) 4769 bp
Sequence 4: XM_014145553.1 (*col2a1ad*) 5964 bp

Sequences (1:2) Aligned. Score: 77.3696
Sequences (1:3) Aligned. Score: 61.8578
Sequences (1:4) Aligned. Score: 59.0366
Sequences (2:3) Aligned. Score: 74.3133
Sequences (2:4) Aligned. Score: 67.0523
Sequences (3:4) Aligned. Score: 91.9899

```
CLUSTAL 2.1 multiple sequence alignment


XM_014134054.1      -CGGACACATAAAACTGCAGTACCTGATCTGACGCGGGTCGGTACTAGATTTCTGGAGGG
XM_014168236.1      --GGACACATAAAACTGCAGTTCCTGACCTGACGCGGGCCGGTACTACGTTTCTGCAGCG
XM_014135018.1      CAGTGGACATAAAACCACGGTTCTTGCTCTGACGCGG-TGGGTGTAAAGTGCCGGCTGGG
XM_014145553.1      ---TGGACATAAAACCGCGGTTCTTGCTCTGACGCGG-TGGGTGTTAAGTGCCGGCTAGG
                          *********  * ** * **  *********   ***   *  *  * *    *

XM_014134054.1      GTCTGGAATCCCCATTGGGTTTTTTTTTTTTGTTTTGAAGGTGGTTGAATATATGGTTCG
XM_014168236.1      GTCTGGGTC------TGGCTTCCCCATCGTTTTTTGGAAGGTGGTTGAATGTATGGTTCT
XM_014135018.1      GCCCCGCGT------TGGATTCCACTTCGAT-TTTGGAAGG---TTGACTGCATGGTTCC
XM_014145553.1      GCCCCGCGT------TGGATTCCACTTCGAT-TTTGGAAGG---TTGACTGCATGGTTCG
                    * *  *         *** **     *   * *** *****   **** *  ******* 

XM_014134054.1      GCTCT-GTAACTGGGAACACACAGAACAACAACAAAAAGAGAGAAGTCCAATTCCTTTCG
XM_014168236.1      GCTCT-GTAACTTGAAACACAAAAAGAGAGAGAGAAGCCTCCGTCCGCCAAC----TGCG
XM_014135018.1      CTTGTAGTCTCTGGGAACACACAT-TACACACCGCTGAAAGAGAGCTGCGTCTGC-TCTG
XM_014145553.1      GTTGTAGTCTCTGGGAACACACAAATACACTCCATTAAAAGAGAGCTGCGTCTGA-TCCG
                      * * **  ** * ****** *     *             *     *       *  *

XM_014134054.1      TCCTTTCTTTTTCTTCTCCCCTAAAAGGCCCTGCATGTCTAACATTTAGACATGTTTAGC
XM_014168236.1      TCCCTTCTTCTGCTTCTCCCCCAAA-GGCACTGCATGTCTAATATTTAGACATGTTCAGC
XM_014135018.1      TCCCATCTTTTTCGTCACCC----AAGGTCCTGCATGTCTAATATTTAGACATGTTCAGC
XM_014145553.1      TCCCATCTTTTTCCTCACCC----AAGGTCCTGCATGTCTAATATTTAGACATGTTTAGC
                    ***  **** * * ** ***    * **  ************ ************* ***

XM_014134054.1      TTTGTGGATTCGAGGACTGTACTGCTACTTGTAGCAACCCAAGTCATTCTATTAGCCGTT
XM_014168236.1      TTTGTGGATTCGAGGACTGTACTGCTACTTGTAGCAACTCAAGTCGTTCTATTTGCCGTT
XM_014135018.1      TTTGTGGATTCACGGACTGTTCTGCTACTTGTAGCATCCCAAGTTGTTTTACTATCCATT
XM_014145553.1      TTTGTGGATTCACGGACTGTTCTGCTACTTGTAGCATCCCAAGTTGTTTTAATATCCATT
                    ***********  ******* *************** * *****  ** ** *  ** **

XM_014134054.1      GTCAAATGTCAGGACGAAGA---CCAGGACCCAGGCAGCTGTATGCAGGACGGACAGAGC
XM_014168236.1      GTCAAATGTCAGGACGAAGA---CCAGGACCCAGGCAGCTGTATGCAGGACGGACAGAGC
XM_014135018.1      GTCAGATGTCAGGAAGAGGATGACCACGTGGC----------------------------
XM_014145553.1      GCCAGATGCCAGGAAGAGGATGACCAGGAGGCAGGCAGCTGCATCCAGGATGCCCATCGC
                    * ** *** ***** ** **   *** *   *

XM_014134054.1      TATAGTGACAAGGACGTATGGAAACCAGAGCCGTGTCGTATTTGTGTGTGCGACACGGGA
XM_014168236.1      TATAGTGACAAGGACGTATGGAAACCAGAGCCGTGTCGTATTTGTGTGTGCGACACGGGA
XM_014135018.1      ------------------------------------------------------------
XM_014145553.1      TATAACGATAAGGATGTGTGGAAGCCGGAGCCATGTCGTATCTGTGTGTGTGACAGCGGG


XM_014134054.1      ACTGTCCTGTGTGATGAAATAATCTGCGAGGAGATTAAAGACTGCCCCAAACCCGAGATC
```

```
XM_014168236.1      ACTGTCCTGTGTGATGAAATAATCTGCGAGGAGATTAAAGACTGCCCCAAACCCGAGATC
XM_014135018.1      ------------------------------------------------------------
XM_014145553.1      TCAGTGCTGTGTGATGAGATCATCTGCGAGGAGATCAGAGAGTGTGCCAACCCCATCATC

XM_014134054.1      CCATTCGGAGAGTGTTGCCCCATCTGCGTGCGGGACGGGGAAGCTCCCCCAAGTGGGGCC
XM_014168236.1      CCATTCGGAGAGTGTTGCCCCATCTGCGTGCGGGACGGGGAAGCTCCCCCAAGTGGGGCC
XM_014135018.1      -----------------------------------------------------------G
XM_014145553.1      CAACCTAGAGAGTGCTGCCCCATCTGCCCTGCGGACGCCAGCGCCCCCACTGAGGCGGTG

XM_014134054.1      CCCGGAGCTAAGGGTCAGAAAGGGGAACCAGGAGATATCACAGATGTCGTAGGACCAAGA
XM_014168236.1      CCGGGAGCTAAGGGTCAGAAGGGGGAACCAGGAGATATCACAGATGTTGTAGGACCAAGA
XM_014135018.1      ATTGGTGCTAAGGGCCAGAAGGGGGAACCCGGCGAGATTGCAGATGTTGTAGGAGCAAAT
XM_014145553.1      ATTGGTGCTAAGGGCCAGAAAGGGGAACCAGGCGAGATTGCAGATGTTGTAGGACCGAAT
                       ** ******** ***** ******** ** ** **  ******* ****** * *

XM_014134054.1      GGACCTGGAGGCCCTTTGGGCCCCCCAGGAGAGCAGGGACCACGTGGAGACAGAGGAGAC
XM_014168236.1      GGACCTGGAGGCCCTATGGGCCCCCCAGGAGAGCAGGGACCACGTGGAGACAGAGGAGAC
XM_014135018.1      GGACCAGCAGGCCCTATGGGCCCCTCAGGCGAGCAGGGACCTCGTGGATCAGCCGGAGCT
XM_014145553.1      GGACCAGCAGGCCCTATGGGCCCCTCGGGCGAGCAGGGACCACGTGGAGAAGCTGGAGCT
                    ***** * ******* ******** * ** *********** ******      ****

XM_014134054.1      AAAGGCGATAAGGGCAGCCCCGGGCCCCGTGGCAGAGATGGCGAGCCTGGGACCCCCGGT
XM_014168236.1      AAAGGCGATAAGGGCAGTCCCGGGCCCCGTGGCAGAGATGGCGAGCCTGGTACCCCTGGT
XM_014135018.1      AAGGGTGATAAGGGAAATGTTGGCCCCAGAGGCAGAGATGGCGAGCCTGGCACCCCCGGG
XM_014145553.1      AAGGGTGATAAGGGAAATGTTGGCCCCAGAGGCAGAGATGGCGAGCCTGGAACCCCCGGG
                    ** ** ******** *     ** *** * ******************** ***** **

XM_014134054.1      AACCCCGGCCCTCCTGGACCTCCAGGACCTAACGGACCCCCCGGCCTTGGAGGAAACTTT
XM_014168236.1      AACCCCGGCCCCCCTGGACCCTCAGGCCCTAACGGACCTCCCGGCCTTGGAGGAAACTTT
XM_014135018.1      AACCCCGGACCCCCTGGCCCACCCGGACCTAACGGACCTCCAGGCCTTGGTGGGAACTTT
XM_014145553.1      AACCCCGGACCCCCTGGCCCTCCAGGACCCAACGGACCCCCAGGTCTTGGCGGGAACTTC
                    ******** ** ***** **  * ** ** ******** ** ** ***** ** *****

XM_014134054.1      GCTGCTCAGATGGCGGGAGGATTCGACGAGAAGGCTGGCGGAGCACAGATGGGCGTGATG
XM_014168236.1      GCTGCTCAGATGGCTGGAGGATTTGACGAGAAGGCTGGCGGAGCACAGATGGGCGTGATG
XM_014135018.1      GCTGCTCAGATGGCTGGTGGATTCGACGAGAAAGCTGGAGGTGCACAGATGGGAGTGATG
XM_014145553.1      GCTGCTCAGATGGCCGGTGGATTCGATGAGAAAGCTGGAGGCGCACAGATGGGAGTGATG
                    ************** ** ***** ** ***** ***** ** *********** ******

XM_014134054.1      CAAGGACCAATGGGGCCTATGGGTCCTCGAGGACCACCTGGCCCTTCTGGAGCTCCTGGC
XM_014168236.1      CAAGGACCAATGGGCCCTATGGGTCCTCGAGGGCCACCTGGCCCCTCTGGAGCTCCTGGT
XM_014135018.1      CAAGGACCTATGGGCCCTATGGGTCCCCGTGGTCCACCTGGCCCTTCTGGAGCCCCTGGC
XM_014145553.1      CAAGGACCTATGGGCCCTATGGGTCCCCGTGGTCCACCTGGCCCTTCTGGAGCCCCTGGC
                    ******** ***** *********** ** ** *********** ******** *****

XM_014134054.1      CCACAAGGTTTCCAAGGCAACCCCGGAGAGGCTGGAGAGCCCGGATCAGCTGGCCCGTTG
XM_014168236.1      CCACAAGGTTTCCAAGGCAATCCCGGAGAGGCTGGAGAGCCCGGATCAGCTGGCCCGTTG
XM_014135018.1      CCACAAGGTTTCCAAGGTAACCCCGGAGAGACTGGAGAGCCAGGCCCAGCTGGCCCACTG
XM_014145553.1      CCACAAGGTTTCCAAGGTAACCCCGGAGAGACTGGAGAGCCAGGCCCTGCTGGTGCACTG
                    ***************** ** ********* ********** **  * *****  *  **

XM_014134054.1      GGTCCCCGTGGTCCACCCGGACCCTCTGGTAAACCTGGAGATGATGGTGAGGCTGGCAAA
XM_014168236.1      GGTCCCCGTGGTCCACCTGGACCCTCTGGTAAACCTGGAGATGACGGTGATGCTGGCAAA
XM_014135018.1      GGACCACACGGACCACCCGGACCTTCTGGCAAACCAGGAAGTGATGGTGAGGCTGGCAAA
XM_014145553.1      GGACCCCAAGGACCCCCTGGACCTTCTGGCAAACCAGGAAGTGATGGTGAGGCTGGCAAA
                    ** ** *  ** ** ** ***** ***** ***** ***  *** ***** *********

XM_014134054.1      CCTGGCAAAGGTGGTGAACGTGGGCCTTCTGGTCCTCAGGGAACTCGTGGATTCCCAGGA
XM_014168236.1      CCCGGCAAGGGTGGTGATCGTGGACCTTCTGGTCCTCAGGGAACCCGTGGATTCCCAGGA
XM_014135018.1      CCTGGAAAGGCTGGTGAGCGTGGACCTGCAGGACCTCAGGGAGCTCGTGGATTCCCCGGA
XM_014145553.1      CCTGGAAAGGCTGGTGAACGTGGACCTGCAGGACCTCAGGGAGCTCGTGGATTCCCCGGA
                    ** ** ** * ****** ***** *** * ** ********* * *********** ***

XM_014134054.1      ACTCCCGGTCTGCCTGGAATCAAGGGGCACAGAGGCTATCCAGGTCTTGATGGACTCAAG
XM_014168236.1      ACTCCCGGTCTGCCTGGAATCAAAGGGCATAGAGGTTATCCAGGTCTTGATGGGCTCAAG
XM_014135018.1      ACTCCTGGTCTTCCTGGAATCAAAGGACACAGGGGTCATCCTGGTCTGGATGGAGCTAAG
```

```
XM_014145553.1      ACTCCTGGTCTTCCCGGAATCAAAGGACACAGAGGTCATCCTGGTCTTGATGGAGCTAAG
                    ***** ***** ** ******** ** ** ** **  **** ***** *****    ***

XM_014134054.1      GGAGAGGGTGGAGCTGCTGGTTCTAAGGGTGAGTCTGGTGCTCCCGGTGAGAATGGCGCT
XM_014168236.1      GGAGAGGGTGGAGCTGCTGGTTCTAAGGGGGAGTCTGGTGCGCCCGGTGAGAATGGCGCT
XM_014135018.1      GGAGAAGGCGGTGCTGCTGGAGCCAAGGGTGAGGGTGGTGCTCCCGGAGAGAACGGTGCG
XM_014145553.1      GGAGAGGGTGGTGCTGCCGGAGCCAAGGGTGAGGGTGGCGCTCCCGGAGAGAATGGCGCC
                    ***** ** ** ***** **  * ***** ***  *** ** ***** ***** ** **

XM_014134054.1      CCTGGACCAATGGGACCACGTGGTCTGCCCGGTGAGAGAGGTCGTCCCGGACCCTCTGGT
XM_014168236.1      CCCGGACCCATGGGACCACGTGGTCTGCCCGGTGAGAGAGGTCGCCCCGGTCCCTCTGGA
XM_014135018.1      TCTGGACCAATGGGACCACGTGGTCTGCCTGGCGAGAGGGGCCGTCCCGGACCTTCTGGA
XM_014145553.1      CCTGGACCAATGGGACCACGTGGTCTGCCCGGAGAGAGAGGCCGTCCCGGAGCTTCTGGC
                     * ***** ******************** ** ***** ** ** *****  * *****

XM_014134054.1      TCTGCTGGCGCTCGTGGTAATGACGGTCTGTCTGGCCCTGCTGGTCCTCCCGGCCCAGTT
XM_014168236.1      GCTGCTGGTGCTCGTGGTAATGACGGTCTGCCCGGCCCTGCTGGTCCTCCTGGCCCAGTT
XM_014135018.1      ACTGCTGGTGCCCGAGGTAATGATGGCTTGCCTGGCCCTGCTGGTCCTCCCGGGCCCGTT
XM_014145553.1      TCTGCTGGTGCCCGTGGTAATGATGGCTTGCCTGGCCCTGCTGGTCCTCCTGGGCCTGTT
                     ******* ** ** ******** **  ** * ***************** ** ** ***

XM_014134054.1      GGTCCTGCTGGTGCTCCAGGCTTCCCCGGCTCTCCAGGTTCTAAGGGAGAAGCTGGCCCC
XM_014168236.1      GGTCCATCTGGTGCTCCAGGCTTCCCCGGCTCTCCAGGTTCTAAGGGAGAAGCTGGCCCC
XM_014135018.1      GGGCCTGCTGGAGCACCCGGTTTCCCTGGATCCCCAGGAGCCAAGGGAGAAGCTGGCCCC
XM_014145553.1      GGACCTGCTGGAGCACCTGGTTTCCCTGGATCCCCTGGTTCCAAGGGAGAAGCTGGCCCC
                    ** **  **** ** ** ** ***** ** ** ** **  * ******************

XM_014134054.1      ACCGGTGCTCGTGGACCTGAGGGCGCTCAGGGACCCCGTGGAGAGTCAGGAACCCCTGGA
XM_014168236.1      ACTGGTGCTCGTGGACCTGAGGGCGCCCAGGGACCACGTGGAGAGTCAGGAACCCCCGGA
XM_014135018.1      ACTGGTGCACGTGGAACTGAGGGTGCACAGGGGCCCCGTGGAGAGGCTGGTACACCCGGA
XM_014145553.1      ACTGGTGCCCGTGGAGCTGAAGGCGCACAGGGACCCCGCGGAGAGGCTGGCACACCCGGA
                    ** ***** ****** **** ** ** ***** ** ** ****** * ** ** ** ***

XM_014134054.1      TCTCCCGGACCCGCCGGTGCATCTGGTAACCCTGGTACTGATGGTATTGGTGGATCTAAA
XM_014168236.1      TCTCCCGGACCCGCCGGCGCTTCTGGTAACCCTGGTACTGATGGTATTGGTGGAGCTAAA
XM_014135018.1      TCTCCTGGACCTGCTGGTGCCTCTGGCAACCCTGGTACTGATGGTATCCCTGGAGCTAAA
XM_014145553.1      TCTCCCGGTCCCGCTGGCGCCTCTGGCAACCCTGGTACTGATGGTATCCCCGGAGCTAAA
                    ***** ** ** ** ** ** ***** ********************    *** *****

XM_014134054.1      GGATCAGCTGGTGCTCCTGGTATCGCTGGCGCGCCTGGCTTCCCCGGGCCCCGTGGCCCT
XM_014168236.1      GGATCAGCTGGTGCTCCTGGTATCGCTGGCGCGCCTGGCTTCCCTGGACCCCGTGGCCCT
XM_014135018.1      GGATCTGCTGGAGCTTCTGGAATTGCTGGTGCACCTGGTTTTCCCGGACCCCGTGGCCCC
XM_014145553.1      GGATCTGCTGGCGCTTCTGGTATTGCTGGTGCACCTGGTTTCCCAGGACCCCGTGGCCCC
                    ***** ***** *** **** ** ***** ** ***** ** ** ** ***********

XM_014134054.1      CCAGGACCCCAGGGAGCCACTGGACCTCTCGGACCAAAGGGAACATCTGGTGACCCCGGT
XM_014168236.1      CCAGGACCCCAGGGAGCCACTGGGCCTCTCGGACCAAAGGGAACATCTGGTGATCCCGGT
XM_014135018.1      CCCGGACCTCAGGGAGCAACAGGACCTCTTGGACCCAAGGGACAGTCTGGAGACCCCGGT
XM_014145553.1      CCCGGACCTCAGGGAGCAACAGGACCTCTTGGGCCCAAGGGCCAGTCGGGAGACCCCGGT
                    ** ***** ******** ** ** ***** ** ** *****    ** ** ** ******

XM_014134054.1      ATCCCAGGTTTCAAGGGTGAGGCTGGACCCAAGGGAGAGCTTGGACCAGCCGGTCCCCAA
XM_014168236.1      ATCCCAGGTTTCAAGGGTGAGGCTGGACCCAAGGGAGAGATTGGACCCGCGGGTCCCCAA
XM_014135018.1      ATTCCTGGCTTCAAAGGAGAGGCTGGTCCCAAGGGAGAGCTTGGCCCTGCTGGTCCCCAA
XM_014145553.1      ATTCCTGGTTTCAAAGGAGAGGCTGGACCCAAGGGAGAGCTTGGCCCTGTTGGTCCCCAA
                    ** ** ** ***** ** ******** ************ **** ** *  *********

XM_014134054.1      GGAGCACTTGGCCCTGCAGGAGAGGAAGGGAAGAGGGGACCCAGAGGAGAGCCCGGCGCT
XM_014168236.1      GGAGCCCTTGGCCCTGCAGGAGAGGAGGGGAAGAGGGGACCCAGAGGAGAGCCTGGCGCT
XM_014135018.1      GGCGCCCTTGGCCCCGCTGGCGAGGAGGGCAAGAGAGGAGCAAGAGGAGAGCCCGGTGCT
XM_014145553.1      GGCGGCCCTGGCCCCGCTGGCGAGGAGGGCAAAAGAGGAGCAAGAGGAGAGCCGGGTGCT
                    ** *  * ****** ** ** ***** ** ** ** *** * *********** ** ***

XM_014134054.1      GCTGGACCTCTTGGACCTCCCGGAGAGAGAGGCGCCCCCGGTAACCGTGGTTTCCCAGGT
XM_014168236.1      GCTGGACCTCTCGGACCTCCCGGAGAGAGAGGCGCCCCCGGTAACCGTGGTTTCCCAGGT
XM_014135018.1      GCTGGACCACTTGGACCTCCTGGCGAGAGGAGCTCCTGGTAACCGTGGTTTCCCAGGT
XM_014145553.1      GCTGGACCACTTGGACCTCCTGGCGAGAGGAGCTCCCGGTAACCGTGGTTTCCCGGGT
                    ******** ** ******** ** ******** ** ** ***************** ***
```

```
XM_014134054.1      CAGGATGGCCTGGCTGGTCCCAAGGGAGCCCCTGGCGAGCGTGGAGTCGGCGGCGTGTCT
XM_014168236.1      CAGGATGGTCTGGCTGGTCCCAAGGGAGCCCCTGGCGAGCGTGGAGTCGGCGGCGTGTCT
XM_014135018.1      CAGGATGGTCTTGCTGGTTCTAAGGGAGCCCCTGGTGACCGTGGTGTTCCCGGCGTGGGT
XM_014145553.1      CAGGATGGTCTTGCTGGTGCTAAGGGAGCCCCTGGTGACCGTGGTGTTCCCGGCGTGGGT
                    ******** ** ****** * ************** ** ***** **   *******  *

XM_014134054.1      GGCCCTAAGGGAGCCGGCGGTGACCCTGGACGCCCTGGCGAGCCCGGTCTTCCTGGCGCA
XM_014168236.1      GGCCCTAAGGGAGCCGGTGGTGACCCTGGTCGCCCTGGCGAGCCTGGTCTTCCTGGTGCA
XM_014135018.1      GGGCCTAAGGGAGGCACTGGTGACCCCGGCCGCACAGGAGAGCCTGGTCTTCCTGGAGCC
XM_014145553.1      GGGCCTAAGGGAGGCACTGGTGACCCCGGCCGCGCAGGAGAGCCTGGTCTTCCCGGAGCC
                    ** ********** *   ******** ** *** * ** ***** ******** ** **

XM_014134054.1      AGAGGTCTTACTGGTCGTCCTGGCGATACTGGTCCTCAAGGCAAAGTTGGACCTGGTGGT
XM_014168236.1      AGAGGTCTTACTGGTCGTCCCGGTGATGCAGGTCCTCAAGGAAAAGTTGGACCCGGTGGT
XM_014135018.1      AGAGGTCTTACTGGTCGCCCTGGAGATGCTGGTCCTCAAGGCAAAGTTGGTCCTGGTGGT
XM_014145553.1      AGAGGTCTCACTGGTCGCCCTGGAGATGCTGGTCCTCAAGGCAAAGTTGGTCCTGGTGGT
                    ******** ******** ** ** *** * *********** ******** ** ******

XM_014134054.1      GCTGCTGGTGAGGATGGTCGCCCCGGACCCCCTGGCCCTATGGGAGCGCGCGGTCAGCCT
XM_014168236.1      TCTTCTGGTGAGGATGGTCGCCCCGGACCCCCTGGCCCTATGGGAGCCCGCGGCCAGCCT
XM_014135018.1      GCTGCTGGTGAGGACGGTCGCCCCGGCCCACCTGGTCCTCAGGGAGCCCGTGGCCAACCT
XM_014145553.1      TCTGCTGGTGAGGACGGTCGCCCCGGCCCACCTGGTCCTCAGGGAGCACGTGGCCAGCCT
                     ** ********** *********** ** ***** ***  ****** ** ** ** ***

XM_014134054.1      GGTGTGATGGGATTCCCCGGACCCAAGGGAGCCAACG-----------------------
XM_014168236.1      GGCGTGATGGGATTCCCCGGACCCAAGGGAGCCAACGGCGAACCTGGCAAGGGCGGAGAG
XM_014135018.1      GGTGTGATGGGATTCCCTGGACCAAAGGGAGCCAATGGTGAGCCTGGCAAGGCAGGAGAG
XM_014145553.1      GGTGTGATGGGATTCCCCGGACCAAAGGGAGCCAATGGTGAGCCTGGCAAGGCAGGAGAG
                    ** ************** ***** *********** *

XM_014134054.1      -------------------------TGAG-------------------------------
XM_014168236.1      AAAGGATTGCTCGGTGCGCCAGGTCTGAGAGGTCTTCCTGGTAAGGATGGTGAGACCGGT
XM_014135018.1      AAGGGTCTTGTGGGTCGTCAAGGTCTGATAGGTCTGGCTGGTAAAGATGGTGAGACTGGT
XM_014145553.1      AAGGGTCTTGTGGGTCGTCAAGGTCTGATAGGTCTGTCTGGTAAAGATGGTGAGACTGGC
                                             ***

XM_014134054.1      ------------------------------------------------------------
XM_014168236.1      GCTGCTGGACCCCCTGGCCCTGCTGGCCCTGTTGGAGAGAGAGGAGAGCAAGGACAACCT
XM_014135018.1      GCTGCTGGACCTCTCGGCCCTCCTGGACCTGCTGGAGAGAGAGGAGAGCAAGGAGCTGCT
XM_014145553.1      GCTGCTGGACCTAACGGCCCTGCTGGACCTGCTGGAGAGAGAGGAGAGCAGGGAGCTGCT


XM_014134054.1      ------------------------------------------------------------
XM_014168236.1      GGACCTTCTGGCTTCCAGGGTCTGCCCGGACCTCCCGGCCCCCCTGGTGAGGGTGGCAAG
XM_014135018.1      GGACCTTCAGGCTTCCAGGGTCTGCCCGGACCTGCTGGCCCCCCAGGAGAGGGAGGCAAG
XM_014145553.1      GGGCCATCTGGCTTCCAGGGTCTGCCTGGACCTGCTGGCCCCCCGGGAGAGGGAGGCAAG


XM_014134054.1      -----------------------------------------TGTCACTGGA---------
XM_014168236.1      CCCGGTGACATGGGAGTTCCAGGAGAGGGTGGAGCTGCTGGTGCCACTGGACCAAGAGGT
XM_014135018.1      CCTGGTGATGCGGGTGTTCCTGGAGAGGCTGGTGCTGCTGGTGCCACTGGACCTAGAGGC
XM_014145553.1      CCTGGTGATGCGGGTGTTCCTGGAGAGGCTGGTGCTGCTGGCGTTACGGGACCCAGAGGC
                                                              *  ** ***

XM_014134054.1      ------------------------------------------------------------
XM_014168236.1      GAGCGTGGTTTCCCAGGAGAGAGAGGAGGTGCTGGGCCTCAGGGGCTGCAGGGACCCCGT
XM_014135018.1      GAGCGTGGTTTCCCTGGTGAGAGAGGCGGTGGTGGACCTCAGGGTCTGCAGGGACCTCGT
XM_014145553.1      GAGCGTGGTTTCCCTGGTGAGAGGGGCGGTGCAGGAACTCAGGGTCTGCAGGGACCTCGT


XM_014134054.1      ---------------------------------------GCGATCGGACCAGGTGGTGCA
XM_014168236.1      GGTCTTCCTGGAACTCCTGGAACTGACGGACCTAAGGGCGCGATCGGACCAGGTGGTGCA
XM_014135018.1      GGACTTCCTGGAACTCCCGGAAGTGATGGACCGAAGGGAGCCATTGGGCCAGCTGGTGGT
XM_014145553.1      GGACTTCCTGGAACTCCCGGAACCGATGGACCCAAGGGAGCCATTGGGCCAACTGGTGGT
                                                           ** ** ** ***  *****

XM_014134054.1      TCGGGTGCCCAGGGACCCCCCGGTCTGCAGGGTATGCCCGGAGAGAGAGGAGCCTCGGGC
```

```
XM_014168236.1        TCGGGTGCCCAGGGACCCCCCGGTCTGCAAGGTATGCCCGGAGAGAGAGGAGCCTCAGGC
XM_014135018.1        GCTGGAGCTCAGGGACCCCCAGGTCTGCAGGGTATGCCAGGAGAGAGGGGAGCTGGTGGA
XM_014145553.1        GCTGGATCTCAGGGACCCCCCGGCCTGCAGGGTATGCCAGGAGAGAGAGGAGCTGGTGGT
                       * **  * *********** ** ***** ******** ******** *****    **

XM_014134054.1        ATCCCCGGACCTAAGGGAGACAGAGGTGACAACGGAGAGAAGGGACCTGAGGGTGCTTCT
XM_014168236.1        ATTCCCGGACCTAAGGGAGACAGAGGTGACAACGGAGAGAAGGGACCTGAGGGTGCTTCT
XM_014135018.1        ATCTCCGGCGCCAAGGGTGACAGAGGTGACAATGGAGAGAAAGGACCTGAGGGCGCTTCT
XM_014145553.1        ATCCTCGGAGCCAAAGGTGACAGAGGTGACAACGGCGAGAAAGGACCTGAGGGCGCTCCT
                      **   ***  * ** ** ************** ** ***** *********** *** **

XM_014134054.1        GGCAAAGACGGCTCAAGAGGTTTGACTGGTCCCATTGGTCCTCCCGGCCCAGCTGGTCCC
XM_014168236.1        GGCAAAGACGGTTCAAGAGGTTTGACTGGTCCAATTGGTCCTCCTGGCCCAGCTGGTCCC
XM_014135018.1        GGAAAAGACGGTTCTAGAGGTTTGACTGGTCCCATTGGTCCTCCTGGCCCAGCTGGTCCC
XM_014145553.1        GGAAAGGACGGTTCTAGAGGTTTGTCTGGTCCCATTGGTCCTCCTGGCCCAGCTGGTCCC
                      ** ** ***** ** ********* ******* *********** ***************

XM_014134054.1        AACGGAGAGAAGGGTGAATCTGGTCCATCTGGACCTTCCGGTGCTGCCGGTGCCCGTGGT
XM_014168236.1        AATGGAGAGAAGGGTGAATCTGGTCCCTCTGGACCTTCCGGTGCTGCTGGTGCCCGAGGT
XM_014135018.1        AACGGCATAAAGGGTGAGGGTGGACCTTCTGGCCCAACTGGTGCTGCTGGTGCCCGTGGT
XM_014145553.1        AACGGCGTGAAGGGTGAGGGTGGACCTTCTGGCCCAACTGGTGCTGCTGGTGCCCGTGGT
                      ** **    ********   *** ** ***** **  * ******** ******** ***

XM_014134054.1        GCTCCTGGAGACAGGGGTGAGACCGGACCTCCTGGGCCTGCTGGCTTCGCTGGGCCTCCT
XM_014168236.1        GCTCCTGGTGACAGGGGTGAGACTGGACCTCCCGGGCCTGCTGGCTTCGCTGGGCCTCCT
XM_014135018.1        GCTCCTGGTGACCGTGGTGAGGGTGGTCCTCCCGGGCCTGCTGGCTTCGCTGGGCCACCT
XM_014145553.1        GCTCCTGGTGACCGTGGTGAGGGTGGTCCTCCTGGGCCTGCTGGCTTCGCTGGGCCTCCT
                      ******** *** * ******   ** ***** *********************** ***

XM_014134054.1        GGATCTGATGGTCAGCCCGGAGCCAAGGGAGAGCAGGGAGAGGGCGGCCAGAAGGGAGAC
XM_014168236.1        GGATCTGATGGTCAGCCCGGAGTCAAGGGAGAGCAGGGAGAGGGTGGACAGAAGGGAGAC
XM_014135018.1        GGTGCAGATGGTCAGCCTGGAGCCAAGGGAGAGTTAGGCGAGGCTGGACAGAAGGGAGAT
XM_014145553.1        GGTGCAGATGGTCAGCCTGGAGCCAAGGGAGAGTTTGGCGAGGCTGGACAGAAGGGAGAT
                      **  * *********** **** **********   ** ****  ** ***********

XM_014134054.1        GCTGGTGCTCCTGGACCCCAGGGACCCTCTGGAGCTCCTGGACCTTCCGGACCTACTGGC
XM_014168236.1        GCTGGTGCCCCTGGACCACAGGGACCCTCCGGAGCTCCTGGACCTTCCGGACCTACTGGC
XM_014135018.1        GGTGGTGCCCATGGACCTCAAGGACCCTCTGGGGCCCCTGGACCTGTGGGTCCCACTGGT
XM_014145553.1        GGTGGTGCCCCTGGACCTCAGGGACCCTCTGGGGCCCCTGGACCTGTGGGTCCCACTGGT
                      * ****** * ****** ** ******** ** ** *********   ** ** *****

XM_014134054.1        GTTTCTGGACCTAAAGGTGCTCGCGGTGCTCAGGGACCCCCTGGTGCCACTGGTTTCCCG
XM_014168236.1        GTTTCTGGACCTAAAGGTGCTCGCGGTGCTCAGGGACCCTCTGGTGCCACTGGTTTCCCT
XM_014135018.1        GTTTCTGGACCTAAAGGAGCTCGTGGTGCCCAGGGGGCTTCTGGTGCTACTGGTTTCCCT
XM_014145553.1        GTTTCTGGATCTAAAGGAACCCGTGGTGCCCAGGGTGGCGCTGGTGCTACTGGTTTCCCT
                      ********* *******  * ** ***** *****     ******* ***********

XM_014134054.1        GGTGCTGCCGGCAGAGTCGGACCCCCTGGTCCCAACGGTAACCCTGGTGCTGCTGGTCCT
XM_014168236.1        GGAGCTGCCGGCAGAGTCGGGCCCCCTGGTCCCAATGGTAACCCTGGTGCTGCTGGTCCC
XM_014135018.1        GGTGCTGCTGGAAGAGTTGGTTCTCCCGGGCCTAACGGTAACCCTGGTGCTGCTGGCCCC
XM_014145553.1        GGTGCTGCTGGAAGAGTTGGATCTCCCGGTCCCAACGGTAACCCTGGTGCTGCTGGCCCC
                      ** ***** ** ***** **  * ** ** ** ** ******************** **

XM_014134054.1        TCTGGCCCTGCTGGTAAAGATGGTCCTAAGGGTGTGCGTGGAGACGGTGGACCCCCAGGC
XM_014168236.1        TCTGGCCCTGCTGGTAAAGATGGTCCTAAGGGTGTGCGTGGAGACGGTGGACCCCCAGGC
XM_014135018.1        GCTGGTCCTGCTGGTAAAGACGGTCCTAAGGGAGTTCGTGGTGATGCTGGAACCCCAGGA
XM_014145553.1        GCTGGTCCTGCTGGTAAAGACGGTCCTAAGGGAGTTCGCGGTGACGCTGGCACCCCAGGA
                       **** ************** *********** ** ** ** ** * ***  *******

XM_014134054.1        AGGCAGGGAGACGCTGGGCTGCGTGGAGCCGCTGGAGCCCCTGGAGAGAAAGGAGATGCT
XM_014168236.1        AGACAGGGAGACGCTGGGCTGCGTGGAGCCGCTGGAGCCCCTGGAGAGAAGGGAGATGCT
XM_014135018.1        AGACAGGGAGACGCTGGGCTGCGTGGAGTTGCTGGAGCCCCTGGCGAGAAGGGAGATGCT
XM_014145553.1        AGACAGGGAGACGCCGGGCTGCGTGGAGTTGCTGGAGCCCCTGGCGAGAAGGGAGATGCT
                      ** *********** *************  ************** ***** *********

XM_014134054.1        GGAGAGGATGGTCCCTCTGGTCCCGATGGTCCTTCAGGTCCTTCAGGTCTGGCTGGACAG
XM_014168236.1        GGAGAGGATGGTCCCCCTGGTCCCGATGGGCCTTCAGGTCCTTCAGGTCTGGCTGGACAG
XM_014135018.1        GGAGGGGATGGTCTCCCTGGTCCTGATGGTCCTTCAGGTCCCCAGGGTCTGGCTGGGTCA
```

```
XM_014145553.1      GGAGAAGATGGTCCCCCTGGTCCTGATGGTCCTTCAGGTCCCCATGGTCTGGCTGGGTCT
                    ****  ******* * ******* ***** ***********    ***********

XM_014134054.1      CGTGGTATTGTTGGTCTTCCAGGACAGCGTGGAGAGAGAGGTTTCCCTGGCTTGCCCGGA
XM_014168236.1      CGTGGTATTGTTGGTCTTCCAGGACAGCGTGGAGAGAGAGGTTTCCCTGGCCTGCCAGGA
XM_014135018.1      CGTGGTATTGTTGGTCTGCCTGGGCAGCGTGGAGAGAGGGGCTTCCCTGGCCTTCCTGGT
XM_014145553.1      CGTGGTATTGTTGGTCTGCCTGGGCAGCGTGGAGAGAGGGGCTTCCCAGGCCTTCCTGGA
                    ***************** ** ** ************** ** ***** *** * ** ** 

XM_014134054.1      CCCTCTGGAGAGCCTGGTAAACAAGGAGCTCCTGGTGGAAGCGGAGACCGTGGACCCCCT
XM_014168236.1      CCCTCTGGTGAGCCTGGTAAACAAGGATCTCCTGGTGGAAGCGGAGACCGTGGGCCCCCT
XM_014135018.1      CCTTCTGGAGAGCCCGGTAAGCAGGGAGCTCCTGGTGCTGGTGGTGACCGTGGACCCCCT
XM_014145553.1      CCTTCTGGAGAGCCCGGTAAGCAGGGAGCTTCTGGTGCTGGTGGTGACCGTGGACCCCCT
                    ** ***** ***** ***** ** *** ** ******   * ** ******** ******

XM_014134054.1      GGCCCAGTGGGACCCCCTGGACTGACTGGACCCGCTGGAGAGACCGGTAGAGAGGGCAAC
XM_014168236.1      GGCCCTGTGGGGCCCCCTGGACTGACTGGACCCGCTGGAGAGCCCGGTAGAGAGGGCAAC
XM_014135018.1      GGGCCTGTGGGGCCTCCTGGACTGTCGGGACCTTCTGGCGAGCCCGGAAGAGAGGGCAAC
XM_014145553.1      GGCCCTGTGGGACCCCCTGGACTTTCTGGACCTGCAGGAGAGCCCGGAAGAGAGGGCAAC
                    ** ** ***** ** ********  * *****  * ** *** **** ************

XM_014134054.1      GCTGGTTCTGATGGACCACCCGGTAGAGATGGATCCACTGGAATCAAGGGTGACCGTGGT
XM_014168236.1      GCTGGTTCTGATGGACCCCCTGGTAGAGATGGAGCCACTGGAATCAAGGGTGACCGCGGT
XM_014135018.1      GCTGGATCTGATGGGCCCCCTGGTAGAGATGGATCCACCGGAGTCAAGGGTGAGCGTGGT
XM_014145553.1      GCTGGATCTGATGGACCCCCTGGTAGAGATGGATCCACTGGAGTCAAGGGTGAGCGTGGT
                    ***** ******** ** ** ************ **** *** ********** ** ***

XM_014134054.1      AACACCGGTCCTGCTGGTGCTCCTGGCTCCCCAGGAGCTAATGGTTCCCCCGGCCCTGTC
XM_014168236.1      AACACCGGTCCTGCTGGTGCTCCTGGCGCCCCAGGAGCTAATGGTTCCCCCGGCCCTGTC
XM_014135018.1      AACACTGGTCCTGCAGGTGCACCTGGTGCCCCTGGCGCTCCAGGTGCCCCCGGCCCTGTT
XM_014145553.1      AACACTGGCCCTGCTGGTGCTCCTGGTGCCCCAGGCGCTCCCGGTGCCCCCGGCCCTGTC
                    ***** ** ***** ***** *****  **** ** ***   *** ************* 

XM_014134054.1      GGCCCCACCGGCAAGCAAGGAGACAGGGGAGAGGCTGGAGCTCAAGGACCGGCTGGGCCT
XM_014168236.1      GGCCCCACCGGCAAGCAGGGAGACAGGGGAGAGGCTGGAGCTCAAGGACCAGCTGGACCT
XM_014135018.1      GGCCCCCTGGGCAAGCAGGGAGACAGAGGAGAGGGAGGTGCTCAAGGACCTGCCGGACCC
XM_014145553.1      GGCCCCCTGGGCAAGCAGGGAGACAGAGGAGAGGGAGGTGCTCAAGGACCTGCCGGACCC
                    ******   ******** ******** *******  ** *********** ** ** ** 

XM_014134054.1      TCCGGGCCTGCTGGAGCCAGAGGAATGGCTGGACCCCAAGGACCCCGTGGAGACAAGGGT
XM_014168236.1      GCCGGGCCTGCTGGAGCCAGAGGAATGGCTGGACCCCAAGGACCCCGTGGAGACAAGGGT
XM_014135018.1      GCTGGACCAGCTGGCGCTAGAGGAATGGCTGGACCCCAAGGACCCCGTGGAGACAAGGGA
XM_014145553.1      GCTGGACCAGCTGGCGCTAGAGGAATGGCTGGACCCCAAGGACCCCGTGGAGACAAGGGA
                     * ** ** ***** ** ***************************************** 

XM_014134054.1      GAGGCTGGAGAGGGTGGCGAGAGAGGACAGAAGGGACACAGAGGATTCACTGGTCTGCAG
XM_014168236.1      GAGGCTGGAGAGGGTGGTGAGAGAGGACAGAAGGGACACAGAGGATTCACTGGTTTGCAG
XM_014135018.1      GAGGCTGGCGAGACAGGAGAGAGGGGACAGAAGGGACACCGTGGCTTCACCGGTCTGCAG
XM_014145553.1      GAGGCTGGCGAGACAGGAGAGAGGGGACAGAAGGGACACCGTGGCTTCACTGGTCTGCAG
                    ******** ***   ** ***** *************** * ** ***** *** *****

XM_014134054.1      GGTCTGCCCGGACCTCCCGGTCAATCTGGAGACCAGGGTGCCTCTGGACCTGCCGGACCA
XM_014168236.1      GGTCTGCCTGGACCTCCCGGTCAAGCTGGAGACCAGGGTGCATCTGGACCTGCCGGACCA
XM_014135018.1      GGTCTTCCCGGACCTCCAGGCTCCGCTGGAGACCAGGGAGCTGCTGGACCTGCCGGACCG
XM_014145553.1      GGTCTTCCCGGACCACCCGGCTCCGCTGGAGACCAGGGAGCTGCTGGACCTGCCGGACCT
                    ***** ** ***** ** **     ************* **  **************** 

XM_014134054.1      AGTGGATCAAGAG-------------TGAGATTCTATCT-----------TTCTGTCTGT
XM_014168236.1      AGTGGATCAAGAGGACCCCCCGGACCTGTTGGTCCCTCTGGAAAGGATGGTTCCAACGGT
XM_014135018.1      AGTGGCGCTAAGGGACCCCCTGGCCCAGTCGGCCCCGCTGGTAAGGATGGATCTAACGGT
XM_014145553.1      AGTGGTGCTAAAGGACCCCCTGGCCCAGTCGGTCCCGCTGGTAAGGATGGATCTAACGGT
                    *****  * *  *              *     *   **            **   * **

XM_014134054.1      GTATC--------TCAG-------------------------------------------
XM_014168236.1      ATGCCCGGCCCCATCGGACCTCCCGGACCCCGTGGTCGCTCTGGAGAGACTGGTCCCTCT
XM_014135018.1      CAGCCTGGACCCCTCGGACCCCCTGGACCTCGTGGTCGTTCTGGAGAAACTGGCTCTGCT
XM_014145553.1      ACCCCTGGACCCCTCGGACCCTCTGGGCCTCGTGGTCGTTCTGGAGAGACTGGTCCTGCT
                        *        ** *
```

```
XM_014134054.1      GGTCCACCTGGAAACTCTGGACCCCCTGGTCCTCCTGGTCCTCCCGGCCCTGGTATCGAC
XM_014168236.1      GGTCCCCCTGGAAACTCCGGACCCCCTGGTCCTCCTGGTCCTCCCGGCCCTGGTATCGAC
XM_014135018.1      GGTCCACCTGGTAACCCCGGACCCCCTGGTCCACCTGGTCCTCCCGGCCCTGGCATCGAC
XM_014145553.1      GGTCCTCCTGGTAACCCCGGACCCCCTGGTCCTCCTGGTCCTCCCGGCCCTGGCATCGAC
                    ***** ***** *** * ************** ******************** ******

XM_014134054.1      ATGTCTGCCTTCGCTGGTTTGTCTCAGCCTGAGAAGTCCCCCGATCCCCTGAGGTACATG
XM_014168236.1      ATGTCTGCCTTCGCTGGCCTGTCTCAGCCTGAGAAATCCCCCGATCCCCTGAGGTACATG
XM_014135018.1      ATGTCTGCCTTTGCTGGCCTGTCTCAGCCTGAGAAGTCTCCGGATCCCCTGAGGTACATG
XM_014145553.1      ATGTCTGCCTTCGCTGGCCTGTCTCAGCCTGAGAAGTCTCCGGATCCCTTGAGGTACATG
                    *********** *****  **************** ** ** ****** ***********

XM_014134054.1      AGGGCCGACCAGGCCTCCGGAAACCTGAGGACGCACGACGCCGAGGTGGACGCCACGCTC
XM_014168236.1      AGGGCCGACCAGGCCTCCGGAAACCTGAGGACGCACGACGCCGAGGTGGACGCCACACTC
XM_014135018.1      AGGGCTGATGAGGCGTCCAGCTCCCTGAGGCAGCACGACGTGGAGGTGGACTCCACACTC
XM_014145553.1      AGGGCTGATGAGGCATCCAGCTCCCTGAGGCAGCATGACGTGGAGGTGGACTCCACACTC
                    ***** **  **** *** *   *******  *** ****  ********* **** ***

XM_014134054.1      AAGTCCCTCAACAACCAGATCGAGAACATCCGCTCCCCCGAGGGCTCCAAGAAGAACCCT
XM_014168236.1      AAGTCTCTCAACAACCAGATTGAGAACATCCGCTCCCCTGAGGGCTCCAAGAAGAACCCT
XM_014135018.1      AAGTCCCTCAACAACCAGATCGAGAACCTGCGCAGCCCTGATGGTAGCCAGAAGAACCCT
XM_014145553.1      AAGTCCCTCAACTACCAGATCGAGAACCTGCGCAGCCCCGACGGTAGCCAGAAGAACCCC
                    ***** ****** ******* ****** * ***  *** ** **   * **********

XM_014134054.1      GCACGTACTTGCAGAGACCTGAAGCTGTGCCACCCCGACTGGAAGAGCGGAGAGTACTGG
XM_014168236.1      GCACGTACTTGCAGAGACCTGAAGCTGTGCCACCCTGACTGGAAGAGTGGAGAGTACTGG
XM_014135018.1      GCCCGCACCTGCAGAGACCTCAGACTGTGCCACCCAGAGTGGAAGAGCGGTGACTACTGG
XM_014145553.1      GCCCGCACCTGCAGAGACATCAAACTGTCCCACCCCGAGTGGAAGAGTGGTGACTACTGG
                    ** ** ** ********* * *  **** ****** ** ******** ** ** ******

XM_014134054.1      ATCGACCCTAACCAGGGCTGCACCATAGACGCCATCAAGGTCTACTGTAACATGGAGACT
XM_014168236.1      ATCGACCCTAACCAGGGCTGCACCATAGATGCCATCAAGGTCTACTGTAACATGGAGACT
XM_014135018.1      GTGGATCCTAACATTGGTAGCACAGCTGATGCCATCAAGGTCTTCTGCAACATGGAGACT
XM_014145553.1      GTGGATCCTAACATTGGCAGCACAGCTGACGCCATCAAGGTCTTCTGCAACATGGAGACT
                     * ** ******   **  ****    ** ************* *** ************

XM_014134054.1      GGAGAGTCCTGCGTCTACCCCAAGCCTGCCAGCATCCCCAAGAAGAACTGGTGGTCCAGC
XM_014168236.1      GGAGAGTCCTGCGTCTACCCCAAGCCTGCCAGCATCCCCAAGAAGAACTGGTGGTCCAGC
XM_014135018.1      GGCGAGACCTGTGTGTACCCCAGCATAGCCAACGTGCCTCAAAAGAACTGGTGGACAAGC
XM_014145553.1      GGCGAGACCTGTGTGTACCCCAGCATAGCCAATGTACCGCACAAGAACTGGTGGACAAGC
                    ** *** **** ** *******     ****   * **  * ************ * ***

XM_014134054.1      AAGAGCAAGGCTGCCAAACACGTCTGGTTCGGAGAGACCATGAACGGAGGATTCCACTTC
XM_014168236.1      AAGAGCAAGGCTGCCAAACACGTCTGGTTCGGGGAGACCATGAACGGAGGATTCCACTTC
XM_014135018.1      AAGAGCAAGGACCGCAAACACGTCTGGTTCGGAGAGACTATGAATGGAGGATTCCACTTC
XM_014145553.1      AAGAGCAAGGACCGCAAACACGTCTGGTTCGGAGAGACCATGAACGGAGGATTCCACTTC
                    **********    ****************** ***** ***** ***************

XM_014134054.1      AGCTATGGTGATGACAGTCTGGCAGCCAACACTGCCAGCGTCCAGATGACCTTCCTGCGG
XM_014168236.1      AGCTATGGCGATGATAGCCTGGCTGCCAACACGGCCAGCATCCAGATGACCTTCCTGCGG
XM_014135018.1      AGCTACGCTGAGGACGGT------ACCAACGCTGCCAGTATCCAGCTGACCTTCCTAAGG
XM_014145553.1      AGCTACGCTGAGGACGGT------ACCAACGCCGCCAGTATCCAGCTGACCTTCTTGAGG
                    ***** *  ** **  *        ***** * *****  ***** ******** *  **

XM_014134054.1      CTGCTGTCCACCGAGGCTAGCCAGAACCTCACCTACCACTGCAAGAACAGCGTGGCCTAC
XM_014168236.1      CTGCTATCCACCGAGGCTAGCCAGAACCTCACCTACCACTGCAAGAACAGTGTGGCCTAC
XM_014135018.1      CTGCTGTCCACGGAAGCATCTCAGAACCTCACCTACCACTGCAAGAACAGCGTGGCCTAC
XM_014145553.1      CTGCTGTCCACGGAAGCATCTCAGAACCTCACCTACCACTGCAAGGACAGCGTGGCTTAC
                    ***** ***** ** **    ************************ **** ***** ***

XM_014134054.1      ATGGACGGGGCCACGGGAAACCTGAAGAAGGCCGTGCTGCTCCAGGGCTCCAACGACGTG
XM_014168236.1      ATGGACGGGGCCACGGGCAACCTGAAGAAGGCTGTGCTGCTCCAGGGCTCCAACGATGTG
XM_014135018.1      ATGGACGCATCCACCAGCAACCTGAAGAAGGGTCTGCTGCTCCAGGGCTCCAACGACGTG
XM_014145553.1      ATGGACGCGTCCACTGGCAACCTGAAGAAGGCTCTGCTGCTCCAGGGCTCCAACGACGTG
                    *******   ****  * *************   ********************** ***

XM_014134054.1      GAGATAAGAGCTGAGGGCAACAGCCGCTTTACATACGCCGTTATGGAGGACGGCTGCACG
```

```
XM_014168236.1      GAGATCAGGGCCGAGGGAAACAGCCGCTTCACGTACACCGTCATGCAGGACGGCTGCACG
XM_014135018.1      GAGATCAGAGCTGAGGGAAACAGCCGCTTCACCTACAGCGTTGTGGAGGATGGCTGCAAG
XM_014145553.1      GAGATCAGAGCTGAGGGCAACAGCCGCTTCACTTACAGCGTGGTGGAGGACGGCTGCAAG
                    ***** ** ** ***** *********** ** ***  ***  ** **** ******* *

XM_014134054.1      AAACATAAAGGAACGTGGGGAAAGACAGTGATTGAGTACAGATCACAGAAGACCAGCCGG
XM_014168236.1      AAACACACAGGAGCATGGGGCAAGACAGTGATTGACTACAGATCACAGAAGACCAGCCGG
XM_014135018.1      AAACACACAGGCCAGTGGGGAAAGACTGTCTTCGAGTACAAAACACAGAAGACCTCCCGT
XM_014145553.1      AAACACACAGGCCAGTGGGGCAAGACTGTCTTCGAGTACAAAACACAGAAGACCTCCCGT
                    ***** * ***    ***** ***** **  * ** **** * ***********  ***

XM_014134054.1      CTGCCCATCGTGGACATTGCTCCTGTGGATATTGGAGGAGCGGACCAGGAGTTTGGTGTT
XM_014168236.1      CTGCCCATCGTGGACATTGCTCCCATGGATATTGGAGGAGCGGACCAAGAGTTTGGAGTC
XM_014135018.1      CTGCCCATCGTGGACATTGCTCCTATGGACATTGGAGGAGCGGACCAGGAGTTCGGTCTG
XM_014145553.1      CTGCCCATCGTGGACATTGCTCCCATGGACATCGGAGGAGCGGACCAGGAGTTCGGTGTG
                    ***********************  **** ** ************** ***** **  *

XM_014134054.1      GACGTCGGCGCAGTTTGCTTCTTGTAAAGAAAGAGGGATGAAAAGAAGGAGAAGTAGAGG
XM_014168236.1      GACGTCGGTGCAGTCTGCTTCTTGTAAAGACAAAGGAATGAAAA--AGGAAAAGCTGAGA
XM_014135018.1      GATGTGGGTGCAGTCTGCTTTTTGTAAAGTGAAATAAACACAACGGCAAAAAAACTAAAA
XM_014145553.1      GACGTGGGCGCAGTCTGCTTCTTGTAAAGTGGAATAAACAAGACGACAAAGAAACTAAAA
                    ** ** ** ***** ***** ********    *   *    *      * **    *

XM_014134054.1      GGAAAAAC-ACAAAATATGAGAGAGA------------GGAACACACACTTCTATCAAAA
XM_014168236.1      AAAAACCCCATAAAAGATGAGAGAGACAGAGAGAGAGAGGAACACACACTTCTAGCAAAA
XM_014135018.1      CATGAAAGAAATAACTCAACTATGAA------------ACAATACACACACACACTCTAC
XM_014145553.1      CATGAAAGAAATAACT-----ATGAA------------ACAATACACACTCCAAAAAAAA
                        *    *  **       *   *              ** ******    *    *

XM_014134054.1      AAAAGGAAGAATGAAAGAAAGAAAAGAGAAAGATAACAACACTTTTATATAATTTTTTGT
XM_014168236.1      T-------GAATGAAAGAGAGAAAAGAAAAAGATCAAGTCACTTTTTT-----------T
XM_014135018.1      A-------AAAAAAGAAGACTAACTTAAAAAAAGGA---AACTTTT-------------T
XM_014145553.1      A-------GACCGGAACTAAAAAAATAAATAATTGA---TACTTTC-------------T
                             *     *     **   * * *    *    *****              *

XM_014134054.1      TTAAGTAAAAAGTGCTTTTTCCAACTAAGTC--CTGCACTGAATGGCACCGGCAATATTC
XM_014168236.1      TTAAGTAAAAAGTGATTTTTCCAACTAGGATATCTGCACTGAATGGCACCGGCAATATTC
XM_014135018.1      CTC-TGGCAAAGTGCTCT------CCAAGTTGTACTCCATGGATGTTAGCACTGAAGGGC
XM_014145553.1      CTTACAAAGAAGTGCTCT------CCAAGTTGTACTCCCTGGATGTTAGCACTGAAGGAC
                     *       ***** * *      * * *       *  ** ***  * *    *    *

XM_014134054.1      ATCTGGGATTTCATGGCCTTCCAGTCTCACTCCATCAGACCCCCCCTCCCATGGTGCCTC
XM_014168236.1      ATCTGTGATTTCATCGACTTCCAGTCTCCCTCCGTCAACCCCTCCCTCC-----------
XM_014135018.1      ACCGGCGTTC-----------CAGCAGTCCCTGTCAACCCACTTCCTGAG----------
XM_014145553.1      ACCAGCAAT-------------ATCCGTATGTAATTACACCATTCC--------------
                    * * *   *             *             *  *    **

XM_014134054.1      AGCCCTATCCCTCCATCCGCCCCACCCCCCACCCTGGGAGCACCTCCCCACTAACAAGAA
XM_014168236.1      ------ATTCTCCCATCTACTTCACCCCCCAGCCTGGGAGCACCTCCCCACCAACAAGAA
XM_014135018.1      ------ATCCAGTCATGTTTCCCATGGCTCACAGTTGAAGGG----------AATGAG--
XM_014145553.1      --------CCAGTCATGTTTCCTGTGGCCCACAGTTGGCGGG----------AATGAGTT
                             *   ***           * **   * *  *            **  **

XM_014134054.1      GGATC----GAGGAGAAAGAGGAGAAAGAGGAGAAAGAGG--AGAAAGAGGCAGCCAA--
XM_014168236.1      GGAACAAAGGAAGAGGAGAAGGAGAGAAAGGGGGAGCATGTCAGAGCATGACAGAAAGAG
XM_014135018.1      ----CC---AGGAGGAAGAGGGACCAAGCCAAGAGACGAGTGATGAA-------------
XM_014145553.1      TGAGCC---AGAAGGAAAAGGGACCAAGCCAAGGGACGAGTGATGGAGAAACAGAAACA-
                        *         * *   ***   *     *      *  *

XM_014134054.1      ---TGAGTTGGTGTTATTTATTTATTGTTCAGGTGTGGGTCCCAGGTGTGTTAGGACTGA
XM_014168236.1      AGCCGAGTTGGTGTTATTTATTTATTGTTCAGGTGTAGGTCCCAGGTGTGTTAGGACTGA
XM_014135018.1      ------------------------------------------------------------
XM_014145553.1      ---TGACTCGATGTCACTTATTTATTGATGAGGTACAG-------------TGGGACTGG


XM_014134054.1      GTGTGTTACCCTGTAAAAAAGGCCCCACGCACAACAACATACCTATTCTATAAGTTCCCC
XM_014168236.1      GTGTGTTACCCTGTAAAAA-GGCCCCACGCACAACAACATACATAACCTATCAGTTCCCC
XM_014135018.1      ------------------------------------------------------------
```

```
XM_014145553.1      GGGGGGCTTCACCATAAGAACCGCAAACTCCTCCCACTACAGAACACCTAGAACCTCCCA

XM_014134054.1      ----------------CTTTTCTTGTTCCTCTTCCATACCTCCACACCCTAAACATTATA
XM_014168236.1      ACCTCCCCAATCTCCTCTTTTCTTTCTCCTATTCCATACCTTCTCACCCCAAACATTATA
XM_014135018.1      ------------------------------------------------------------
XM_014145553.1      ----------------CACCACCTCCCTCTCCGACCTAGCTACAAATACGCCATTTTGAA

XM_014134054.1      TCAAATCCAAAACCAAAACGTCTAACAGTGTGATAGGTGCAGGTGTTCCTACTTCCAACC
XM_014168236.1      TCAAATCCAAACCAAAAATGG--AGCAGTGTGATAGGTGCAGGTGTTCCTAGTTCCAACC
XM_014135018.1      ------------------------------------------------------------
XM_014145553.1      TCAA-TGTAGACCAAAAAAAT---ATTTTCAGTAATTATAAGGTGCTCAAAATGGTTTTT

XM_014134054.1      GCCGCCCCAGTGTGTATCAGGAAGTTACTGTGTGTTATAATAAAGTCAAATGGTGCTATT
XM_014168236.1      GCCGCCCCAGTGTGTATCAGGAAGTTACTGTGTATTATAATATAGTCAAATGGTGCTATT
XM_014135018.1      ------------------------------------------------------------
XM_014145553.1      TTTACCATCAAGACCAGGAGCTAAACTTTAACTGT-GTATTAAAAATACTTGGTTCTATT

XM_014134054.1      GTTGTAAAACAAGTCTGTATTCTTTAACAACCAGATACTAATGTATTAGTAACATGTTTA
XM_014168236.1      GCTGTAAAACCAGTCTGTATTCTTTAACAACCAGATACTGATGTATTAGTGACATGTTTA
XM_014135018.1      ------------------------------------------------------------
XM_014145553.1      GTTGTAAAA---GTCTGTGTTTATAAACAACCAGATGCTTTTCTAT-----ATATTTCCA

XM_014134054.1      AAACGTGTGTACATATATGTATATGTGTGTATACATATATATATATATACATTTTTCA
XM_014168236.1      TAATGTGT----ATATATGTATATGTGTGT------GTATACATATATCTACAGTTTTCA
XM_014135018.1      ------------------------------------------------------------
XM_014145553.1      CATTATGT--------GTGTACATGTGTCT--------ATATGCACACCAACACTTTTTG

XM_014134054.1      GAGAGTACATTTCACGCTGAGATCATTTTTCAGAGTGGTAAATGTTTCTGCCCCTCATCC
XM_014168236.1      GAGAGTACATTTCACACTGAGATTTGTTTTCAGAGTGGTAAATGTCACTGCCCCT-----
XM_014135018.1      ------------------------------------------------------------
XM_014145553.1      GAAAGTGAGGTTACATTTGGGATATGTTTT--------------TCATTGTCCTT-----

XM_014134054.1      CCATCCCAACCCAACCCTGACCCTCCACCATGGTAGCCCAATGACACATAAACCATGTCC
XM_014168236.1      ---TCCC--CCTGACCACAACCCCCCTCATTGGCTGCCCAATGAAACATAAACCGTGTCT
XM_014135018.1      ------------------------------------------------------------
XM_014145553.1      ---TACC-------CCTTGATTGCCCATTAGAGGAA---AATGTCTTACTCAGTTGACAA

XM_014134054.1      ATAATATATTTTGTAAGAGACATGTTCAATTCGGAAT--CAATCCCAGCTCTAAACTTTT
XM_014168236.1      ATCAT---TTTTGTAAGAGACATGTTCAATTCGGGATTTCAATCTCAGATCTAAACTTTT
XM_014135018.1      ------------------------------------------------------------
XM_014145553.1      ACAAC----CCCCCAAAAAATCTAATCAAATTTGGGAAATTAAATCAGTTTTGTTTTGTT

XM_014134054.1      TTGGTTTGTTTTATTTTGTTTTAATTATAATATTTTAAGCTACCTCACACACACACACAC
XM_014168236.1      TT---------TGTTTTGTTTTAATTAT---ATTATAAGCTACCTCACGCTAAAAAA---
XM_014135018.1      ------------------------------------------------------------
XM_014145553.1      GT-----------AGTCATCCTGATTGT-----TTAAAGCTACCTCACGCTGAAAAAC--

XM_014134054.1      ACAAGAAAGAAACAACAAAACATGGTTAAACCCTGTATCCAAGTTTACAAATCCAGCTTG
XM_014168236.1      ---AGAAAGAAACAACTAAGCAAAGTTA---------TCCAAGTTTACAAATCCAGCTTG
XM_014135018.1      ------------------------------------------------------------
XM_014145553.1      ---AAAGTGAAAACGTTGACCTTAAAAA---------TTTTTGCTTTCGGGTGGAACTGG

XM_014134054.1      GGCGATCTTCTCTTTGGTGCATTAGACCCCATGTGGTGAGAAGCCCACCAGAAGTTGACT
XM_014168236.1      GGTGATCATATCTTTGGTGCATGAGACCCCATGTTGTGAGAAGACCACCAGAAGGTGCCT
XM_014135018.1      ------------------------------------------------------------
XM_014145553.1      TGCTGAACAGCCATCTTTGCTTTGGGT----TGCTGT--------CTTCAGAAGGTGCTA
```

```
XM_014134054.1      TTGACCGTTGACCTATCCCACTCCACTTCCTCCCATAAACCCCCCGCTGTCCCCTCACAC
XM_014168236.1      TTAACCCTTGACCTATCCCACTCCACTTTGTCCCATATGCCCCC-ACTCTCCCCTCACAC
XM_014135018.1      ------------------------------------------------------------
XM_014145553.1      TGAGTTCTAGAATTCAGTCTCTGCCATTGTTCCTCCAAACCCCC-ACTAGCCTCACACCA

XM_014134054.1      AGGAAGTACCTTCTGCCCTGGTAGGAAGTACCTCGGCCATGCCC-----CTCTAGGAACA
XM_014168236.1      AGGAAATACCCTCTACCCTGGCAGGAAGTACCTTGGCCCTGGCCACGCCCCGTAGGAACA
XM_014135018.1      ------------------------------------------------------------
XM_014145553.1      CACACACACACACCACCTTGGACGACAGG--TGCAGCCAATCAA-----GGCAGGGAACC

XM_014134054.1      GGA------------CATTAGACTTGA---------------------TATCGCTACT--
XM_014168236.1      GGAACAGGGCAGGGCTGTTAGACTTGAGTGAGTTCTTTCTCACAGTGATATCACTACTAG
XM_014135018.1      ------------------------------------------------------------
XM_014145553.1      AAG---------------TGGCTCTGGAT------------------ATTCTCCTATGGA

XM_014134054.1      -------------CATTTACACACAGAGGGAAAGCACC-ATGACCAGGGCAGAGGGTGCA
XM_014168236.1      ACAATCCAGACCTCCTTTACACACAGAGGAAAACCACCTACGACCAGGCCAGAGGGTAAT
XM_014135018.1      ------------------------------------------------------------
XM_014145553.1      G------------TCTACGCCCCACAAATAGAATCACGGGAGTCCAAGG--AGAGCTACC

XM_014134054.1      TTCGTTTGTCAGGGTGTGTATATATATTTGTTATATTAATTATAATAGTAATATTGTTAA
XM_014168236.1      TTTATTTTTGAGGGTGTGTATAT-TTTGTATTAT-TTAATAATAATAATAGTATTATTGT
XM_014135018.1      ------------------------------------------------------------
XM_014145553.1      CAAAACAACCAGGGTG-GCTTGTCTCTGTGTGTGTGAGGGTGTGTCATTTCTTTTGTTGT

XM_014134054.1      TAACAACAACATCATTACTATTATTATTAGTATTATTAAATATGATGATCATGATTTTGT
XM_014168236.1      TAATAATGATAGCATTACTATTATTATT---------AAATATGATGATCATGATTTTGT
XM_014135018.1      ------------------------------------------------------------
XM_014145553.1      TATTAATTTTATTATGATCATGATCATT-----------------TTTTGTTTGTTTTTA

XM_014134054.1      TTTTGTTACAGTTTTGATTCATGTAAATTGGAAATAAAAGGAAAAAAACACAAGTAA---
XM_014168236.1      TTTTGTTACAGTTTTGATTCATGTAAATTGGAAATAAAAGAAAAAACAAGTAACAGACTG
XM_014135018.1      ------------------------------------------------------------
XM_014145553.1      TTTTGTTACACTTTTAATTGATGTAAATTGGAAATAAAAAAGGAAAAAC-CAAGTAA---

XM_014134054.1      ------------------------------------------------------------
XM_014168236.1      AAGTGGCTTTTTTAATTTTATTTTTGGGGGGATAATTGTTATTTATCCCCATATTGCTCC
XM_014135018.1      ------------------------------------------------------------
XM_014145553.1      ------------------------------------------------------------

XM_014134054.1      ------------------------------------------------------------
XM_014168236.1      AGAGAAAAGTGTGCATCTGGGAAGGTCTTTTTTCTGTACCCTGAAAGTATGATTAAAACC
XM_014135018.1      ------------------------------------------------------------
XM_014145553.1      ------------------------------------------------------------

XM_014134054.1      ------------------------------------------------------------
XM_014168236.1      TTTTCTGTTGCTATTATGTTCCCAGAGATAGAAGCAATACCAAATCTTGATCCTGTGTCC
XM_014135018.1      ------------------------------------------------------------
XM_014145553.1      ------------------------------------------------------------

XM_014134054.1      ------------------------------------------------------------
XM_014168236.1      TGTATGCACTCTGCCTTGGTCTGTGTGTTTGGGCTAAATGCATATCGAGGGGTGGGATGC
XM_014135018.1      ------------------------------------------------------------
XM_014145553.1      ------------------------------------------------------------

XM_014134054.1      ------------------------------------------------------------
```

```
XM_014168236.1      AGTCAGAACACACAAGGAGAGACAGTGGAGAGGTGGATGATGGTGAGCCGCCATTTTCTT
XM_014135018.1      ------------------------------------------------------------
XM_014145553.1      ------------------------------------------------------------

XM_014134054.1      ------------------------------------------------------------
XM_014168236.1      TTTGTTTTATTTGTTGGAGAGAAAAGGCAAAATTTAGTGAGGAAGTGGAAAGTTCATATC
XM_014135018.1      ------------------------------------------------------------
XM_014145553.1      ------------------------------------------------------------

XM_014134054.1      ------------------------------------------------------------
XM_014168236.1      ATGTACTATTTTGTATGTGTAAAATAGTTTTGCAATATTTTCAGATCGTTTTTAAGCATG
XM_014135018.1      ------------------------------------------------------------
XM_014145553.1      ------------------------------------------------------------

XM_014134054.1      ------------------------------------------------------------
XM_014168236.1      GTAGATAGAGTAGGAATGGGAAACATTGCCAACCACAATAATCAGTAACTGTATGGGTTT
XM_014135018.1      ------------------------------------------------------------
XM_014145553.1      ------------------------------------------------------------

XM_014134054.1      ------------------------------------------------------------
XM_014168236.1      ATTATGTCATCCTGTACTTTTTATTTCATTTTCTTTCCTGACTGTTCACCATAGCAATAA
XM_014135018.1      ------------------------------------------------------------
XM_014145553.1      ------------------------------------------------------------

XM_014134054.1      ------------------------------------------------------------
XM_014168236.1      AAGATAGAAGCAGTTCTCTTAACACAGAGACACGTTTGTTTCATTTTTTATATACATTTA
XM_014135018.1      ------------------------------------------------------------
XM_014145553.1      ------------------------------------------------------------

XM_014134054.1      ------------------------------------------------------------
XM_014168236.1      TTTCCTAAACTTGGTTTTAAAGTTCATTTCATATTTAATGTATTGGTTTCCAAATCTATG
XM_014135018.1      ------------------------------------------------------------
XM_014145553.1      ------------------------------------------------------------

XM_014134054.1      ------------------------------------------------------------
XM_014168236.1      ATGTAATCTTTGTTTTAATAACTATTGACATAAAGAACAACTGGTAAACAAATCAACAAA
XM_014135018.1      ------------------------------------------------------------
XM_014145553.1      ------------------------------------------------------------

XM_014134054.1      -----------------------------------
XM_014168236.1      AATACATCACAAATAAAACAACTTTGTAAAAATGA
XM_014135018.1      -----------------------------------
XM_014145553.1      -----------------------------------
```

CLUSTAL 2.1 Multiple Sequence Alignments for *btc*

Sequence 1: XM_014129351.1 (*btca*) 2887 bp
Sequence 2: XM_014138426.1 (*btcb*) 1792 bp

Sequences (1:2) Aligned. Score: 56.5848

```
CLUSTAL 2.1 multiple sequence alignment


XM_014129351.1      CCATTCAAACCTCTAGCCTGCGCAGTATCTCTTGCCGG-TGTAGTTCATTCACCCGTACG
XM_014138426.1      -------AACCTCTAAACTGCGCA--ATCTCTAGCCTACTGTAGTT----CACCAGTGCG
                           ********  *******  ****** ***   *******    **** ** **

XM_014129351.1      CGATCACGCATGAAGGAACCACATTACTCACCACACGTGAGTCGGGAAGCAGGGTATCCT
XM_014138426.1      CGATTTTGCT-----GGACTACATTATTC-------GTGAGTTGGGACACAGAGTATCCT
                    ****   **      * ** ****** **       ****** ****  *** *******

XM_014129351.1      ACTAAACCGAGGATATAGTGAACTTTTTGACCGGATTTTATAGAAGAAACGAAATCTACG
XM_014138426.1      ACTAAGAAGATATTTTGGTGAACTTTT-AAGAGGATTGGATAAAATGAACTCAATCTGCT
                    *****   **   * * **********  *  *****  *** **  ***  ***** *

XM_014129351.1      TTTACCCAT--TTATCCACCCGGAAAAGTATATTCGTTGCTGACGGACATGGCAAAGGCA
XM_014138426.1      TTTAACTATATTTATCCACCTGGAAAAGTAGATAACTTGCCCACGAACATGGCAAAGGCA
                    **** * **  ********* ********* **   ****  *** **************

XM_014129351.1      TACAAACTGTATGTCGGAATAGCCACAGCTTTGGCCCTATGCAAATGCTCTCTGGCTGAA
XM_014138426.1      TACAATCTGTATGTAGGAATGGTAACAGCTTTGGCCCTGTGTAAATACTCTCTGGCTGAA
                    ***** ******** ***** *  ************** ** **** *************

XM_014129351.1      TGGAATGCAACTGAGGAGACGGCCAGCAAGACTGTGTCCCGCCATCACCAAGGCAACACA
XM_014138426.1      TTGAATACAACTGAGCAGCCAGCGAACAACACTGTGTCCCTCCATCACCAAGGCAACACA
                    * **** ******** ** * ** * *** ********** *******************

XM_014129351.1      AACAACTTCACAGACTCAATAGAAACTGCAACTCAAGCCAAATGGAGCGGCCATTTCACC
XM_014138426.1      AACAACTTCAAAGACTCAATAGAAACTGCAACTCAAGCCAAAAAGAGCGGCCATTTCACC
                    ********** *******************************  ****************

XM_014129351.1      AAATGTCCAAAGGAGTTCAGGAATTACTGTATCCATGGGTCGTGCCGCTTTGTGAAGGAA
XM_014138426.1      AAATGTACAAAGGAGTTAAGGCAATACTGTATCCATGGGTCGTGTCGCTTTGTAAAGGAA
                    ****** ********** *** * ******************** ******** ******

XM_014129351.1      CATGACACTCCTTCATGCAGATGTGAAAAAGGGTACATTGGGTCCAGGTGTGAGTATGTT
XM_014138426.1      CAGAATACTCCTTCATGCAGATGTGAGAGAGGGTACATTGGGTCCAGGTGTGAGTATGTT
                    **  * ******************** * *******************************

XM_014129351.1      GATTTGGCCTGGCGTATAGGAGACCAGAGGCAGATCATCATAGCCTGTGTGATAGCAGCG
XM_014138426.1      GATCTGGCCTCGCGTCTAGGTGACAAGAGGCAGATCATCGTAGTCTGTGTGATAGCAGCG
                    *** ****** **** **** *** ************** *** ****************

XM_014129351.1      TTGGTCTTCCTCATACTGCTCATCATATTCATCTGCATCTGTGCACATCGACATAAACTT
XM_014138426.1      TTGGTCTTCCTCATACTGCTCATCACATTCATCTGCATCTGTGCACATCGACATAAACTT
                    ************************* **********************************

XM_014129351.1      TGCAGGCGGAAGAGGAGAAGGAAAGAGGAGACGAGGAATGGAACAGAGAAGCTCAATATG
XM_014138426.1      TGCAGACGGAAGAGGAAAAGGAAAGAGGAGACGAGGAATGGAACAGAGAAGCTCAATATG
                    ***** ********** *******************************************

XM_014129351.1      ATCATGATGA---ACACAAATGGAACACATGGAGCTTCATCAGATTCAGTAGAAACCTCA
XM_014138426.1      ATTATGATGATGAACACAAATGTAATGCATGTAGCTTCATCAGATTCAGTAGAAACCTCA
                    ** *******   ********* **  **** ****************************

XM_014129351.1      GACATCAATGCAATATGAT--GCGGTATGTACTGCATGTCTTGAGTGTCCAAGACACCTT
XM_014138426.1      GACACCAATGCAGTATGACTAGCGGCGGGGACT--ATAATTGGTATTCTCCTGCCGGACG
                    **** ******* *****   ****   * ***  **   * *  *   *  * *
```

```
XM_014129351.1      GGACGTTTCTTCTCACCTTTGATGCCCCTTTATTATCCTTAGTTTGTTCTCTTGTTGTGC
XM_014138426.1      GGATGAGGGCCTTGGTTGTAAGTGCCTGTTGGGAAGAGAGAGCGTG--CTCTAACAGCAC
                    *** *       *     *   ****  **    *     **  **  ****    *  *

XM_014129351.1      CAAGACACACAGCGTCTCCGGCTGTTCTGATTCCAGTGGTATTGTAAAAGACAATTCAAA
XM_014138426.1      TGCGG-ATGTATACTCTTCAGCTGCTTCT----------CACTGCCAACCACAGTCTACA
                       *  *   *   *** * **** *              * **  **  *** *  * *

XM_014129351.1      AACTGACTAGACCCCTCTTGTTGCTGTTGGAAGGTGATATTCCCCAGTTGGATCAAGCCA
XM_014138426.1      GTATG----GACTGTTCTGTGCAGAAATGTAGGAGAACA----CAAGAAAGACTGGACCA
                       **    ***   ***         ** * *   * *    * **   **     ***

XM_014129351.1      AGCATCACATTCTAATTCAGCTCACATCTGAAGGGAGGCCCTGAATGGGAATAAAGCCTG
XM_014138426.1      GCTCCTGGGAACTCATACAACCTTTATGGTCAACATGTAGCTGGATCAATAGAACAGCAA
                               ** ** ** *    **    *    *   *** **    * **   *

XM_014129351.1      GTGCTTGGCACTGTCTAAGCCCATCCTTGAACACATTGATACATATTACACAGACAGAGA
XM_014138426.1      CAACTCTGTGCATTTGGAA---ATGTGATAATGTGATAAGGTTTTTCTCAAAGACTCATT
                       **  *  *  *   *    **     **     * *    * *  ** ****  *

XM_014129351.1      GAGAGAGAGCGTTAGACAGAGAGGGAGAGCATTACACAATGTTT---CCACACCCTA-TA
XM_014138426.1      T-GTGTAAGCAAAATATACAAGGTCATCAAATCACAATAAGTTCAAGTAAAACCATAATT
                      * *  ***   * * * *  *  *    ** ***  * ***      * *** ** *

XM_014129351.1      TAGCCCATTTTCTGTGTGGGCCAGGCCGGTTGCAGGGTGACTTAGTATCTGAATTTGGCT
XM_014138426.1      TAGAGGATATTTTGATGGACTCTGGGCTCTGAAGGAGTAGGCCTTTTTATGAACTGTCGT
                    ***   ** ** **   *   * ** *  *    * **       * * **** *    *

XM_014129351.1      GTAACAGTCAGATCAAAGCAGACGTGCAGCTGGTTCCTCACTATGCCTGCCTGGCTGCCT
XM_014138426.1      GTAA---TGTAACCTACTCTTATTTGGGCCTGGATTT----AATGATTAACATG-TATTC
                    ****   *   * * *  *  *  **   **** *       ***  *  *  * *

XM_014129351.1      GCCCCTACAAAAGGTAATATGAACACACATGGACCTAACAAGCCTCGGGCATAATGATGG
XM_014138426.1      AAGCTAACAGTCCA-GGTATGCTTACTCCTGGATGTGATACGTGTCATA-ATGAGGACTC
                       *  ***        ****   ** * ****  * * * *  **    ** * **

XM_014129351.1      ATG---TGGAGCTGTTAAAGTGGTGTTCAG-AGGCTGGGTCTGACAAGGAATCTGGGGTC
XM_014138426.1      ATGCCATGTAGGCCCCTATGTGGGACAGATTATACTAGGTCTAATA------CTGGTAAA
                    ***   ** **      * ****     *  *  ** ***** * *      ****

XM_014129351.1      ATTTTAAACATGCTATCCATCTCTCTTTTAACTGCTTATTCAGTGCTTATACAAACGCTG
XM_014138426.1      TGATGTAACATACTATTCGTACACTTGAAAAGGTGTGATTTA----TCAAACAATCCTAC
                       *  ***** **** * *     *   **    * *** *    * * **** *

XM_014129351.1      CTCTGTAAT-TGTGTGGCAAAAACAAATTTCACTTCTATAGTAACAACTGTCGCTTTTAT
XM_014138426.1      CAGCGTCATACGCACACCGGTAGGAGATAAAATTGATA-AATACCAATTGT---TCTAAT
                    *   ** **  *     *   *  * **   * *  ** * ** *** ***   * * **

XM_014129351.1      CTTCCAGCGACGACGGGGTCTATAAATGGTAATCTCCTGCCGGACGGGATGATGGCCTTG
XM_014138426.1      CCTC----AGTGCTTGTGCATAACCTTGGCTATTTGCGTTTCGA------AACGCCTCTA
                    * **       *   * *  **    ***  ** * *     **       * * *  *

XM_014129351.1      GTTATGAGTGAATGTTGGAAAGAGAGGGTGCTTTAACGTCACTGAGGAGATATACAAGCC
XM_014138426.1      ATTAACCATATATGTTCAAAAT---CAGCTCTTTAAAACCTGTGGGGA-ATATACAACGC
                     ***    *  *****  ***      *  ******   *  ** *** ********  *

XM_014129351.1      TGTACTCTCCAGCTGCTTTTCACTGCCGACCATAGTCTACAATATGGACAGTATGGACTC
XM_014138426.1      AGTAATGATATACAACAGTGCAAC-CCAAACATTTTTTTCCCTCAAGACTGAATTAGAGG
                     *** *      *  *  * **   ** * ***  * * *  *   *** * **

XM_014129351.1      TGTTCTGTGCAGAAATATAGTACAATACAAGAGAGACTGTTATGGTCACCATGAATCCAC
XM_014138426.1      TGCTAGTTTCAGAGATTGAGTACAACCAAAG-----TCGTGGCAGTTACGTGGAGT----
                    ** *   * **** **  *******   ***       **    ** **   ** *

XM_014129351.1      AGAGATGGATCAACTAAACCAGTATCTCCCTGTGCGTTCTGTAAATGTGATAATGTGATG
XM_014138426.1      -GGAACAGGGAAACCCAAG-AGTAGACTCAGATGAG----GAGACTGGGATGAAGTACCG
                     *  *  *   ***  **  ****    *   ** *    *  * ** *** * **   *
```

```
XM_014129351.1      AAGTTGCCCTTATAGACTCATTTATGCAAACAAAATGTGCATGGTCATCAAATCACAATA
XM_014138426.1      AAGGTA--TTTATTGAAACA---GGGGGAAGATGGAGTGCAGGC----------------
                    *** *    **** **  **     *  ** *    ***** *

XM_014129351.1      AGTTAGAGTTAAACCTTTTTTTAGTTTATGTATCATGGACACTGAGCTCTGAAGGAGTAG
XM_014138426.1      ------------------------------------------------------------

XM_014129351.1      GCCTTTTTATGGACTGTAACCACCTCTTATTTGGGCATAGATTTAATGATTAACATGTAT
XM_014138426.1      ------------------------------------------------------------

XM_014129351.1      TCAAGCTGAAAGCCCAGGTATGCTTACTCGCAGATGTGGCGTGTTATAATGATGACTCAT
XM_014138426.1      ------------------------------------------------------------

XM_014129351.1      GCCATGTATGTGGGACAGATTATACTATGTCTAATGTAGGTAAATTATCAATATAATAAA
XM_014138426.1      ------------------------------------------------------------

XM_014129351.1      TAGGTGGGTGCTTACATTTGCTCTATTTTACACATGTATAGTGTGTATTATACACATGTG
XM_014138426.1      ------------------------------------------------------------

XM_014129351.1      TATTGGACTTTTATTTTGCATATCCCACTCCAACTGAGAGTGGGGTCACAGCCAGGGATC
XM_014138426.1      ------------------------------------------------------------

XM_014129351.1      CACCATTATTGACAGAAACCCTGGAGCAATTAGGGTTAAGTGCCTTGCTCAAGAGCATAT
XM_014138426.1      ------------------------------------------------------------

XM_014129351.1      CTGCAGATTTTTCACCTAGTTGGCTCGGGGATTTGAACCAGTGGCCTTTTGGTTACTGGC
XM_014138426.1      ------------------------------------------------------------

XM_014129351.1      CTAACGCTCTTAACTGCTAGGCTACCTGCCGACCCTGATGTCACCTTATGTAACACACTG
XM_014138426.1      ------------------------------------------------------------

XM_014129351.1      TACATTGTGGTCCATTTGACATTCCTTCAACACATGCTATTATAATGATGTTAATATTGT
XM_014138426.1      ------------------------------------------------------------

XM_014129351.1      TATTAAGCTCTAATAGTTTCCAGCAACTGTTTGATGTATATACATACTGTATATTGTTAC
XM_014138426.1      ------------------------------------------------------------

XM_014129351.1      ATGTAATCAATGTAAAGAGACTCCTTCTATGGGCCAGGGTTGATGTTACTGACTTAAGTT
XM_014138426.1      ------------------------------------------------------------

XM_014129351.1      GGAGGGTGCCCAAATTTGGGTGAGAAGGCTACAACGATAACTTGTAGTGAGCCCATTTGA
XM_014138426.1      ------------------------------------------------------------

XM_014129351.1      TCTTTATATTTCGAGACATGTAGTCTCCTTGCTACCAGAACAACCCTGCCTGTGTGTTAA
XM_014138426.1      ------------------------------------------------------------

XM_014129351.1      TTTGTGCAGCTGTAAAGTAGTCCACTGTTGAAGAAGATTTTGGGGTTTCTCAGCATCCCA
XM_014138426.1      ------------------------------------------------------------

XM_014129351.1      GAGTTTCACATCGTGTATGCAGCCCTTCGCTTCTTCAGCTATGCAAAAGTTTAACATTTT
XM_014138426.1      ------------------------------------------------------------
```

```
XM_014129351.1      CATTAAAACATTTATTTGTGCAGA
XM_014138426.1      ------------------------
```

CLUSTAL 2.1 Multiple Sequence Alignments for *ryr1a*

Sequence 1: XM_014196267.1 (*ryr1aa*) 15647 bp
Sequence 2: XM_014129250.1 (*ryr1ab*) 12869 bp

Sequences (1:2) Aligned. Score: 79.1126

```
CLUSTAL 2.1 multiple sequence alignment


XM_014196267.1      TCATGGTGGACGCCTCCTTCATGCAGACGCTGTGGACCATGAGCCCTGTGATGTCTGGCT
XM_014129250.1      ------------------------------------------------------------


XM_014196267.1      GTGAGCTTGCTGAAGTGTAACATCAACCCCTCAGGCTTTCTGACTGGAGGCTATGTTCTT
XM_014129250.1      ------------------------------------------------------------


XM_014196267.1      AGACTGTTCCATGGTCACATGGATGAGTGCTTGGCTATCCCTGCTGCCGAACAAGGAGAC
XM_014129250.1      ------------------------------------------------------------


XM_014196267.1      GACCAGCGCAGAATTGCTCATTATGAAGGGGGTGCTGTCTGTAGCCATGCCCGGTCACTA
XM_014129250.1      ------------------------------------------------------------


XM_014196267.1      TGGAGACTAGAACCCCTCCGGATTGGATGGAGTGGAGGTCACATGAAATGGGGCCAGTCG
XM_014129250.1      ------------------------------------------------------------


XM_014196267.1      TTCCGAGTGCGTCATATAACCACAGGCCGCTACCTCTGCCTGGATGAAGAGAAAGGACTG
XM_014129250.1      ------------------------------------------------------------


XM_014196267.1      CTGGTGGTGGACCCTGAAAAAGCCAACGCCAAGATGTCAGCCTTCTGCTTCAGAATCTCC
XM_014129250.1      ------------------------------------------------------------


XM_014196267.1      AAGGAAAAGATCGAGGTGGCTCAGAAGCGAGATGTGGAGGGCATGGGCACACCAGAGATT
XM_014129250.1      ------------------------------------------------------------


XM_014196267.1      AAGTATGGGGAGTCCATGTGTTTTGTACAGCACGTCTCCTCTGGCCTCTGGCTTACATAC
XM_014129250.1      ------------------------------------------------------------


XM_014196267.1      GCCTCTGTCGATGCCAAGTCCGCTCGCCTGGGACCTCTTAAGAGGAAGGCCATCCTTCAT
XM_014129250.1      ------------------------------------------------------------


XM_014196267.1      AAGGAGGGCCACATGGACGATGCCCTGACCGTGGCCCGCTCCCAGACAGAGGAGTTCCAG
XM_014129250.1      ------------------------------------------------------------


XM_014196267.1      GCTGCTCGTATGATCTACAACACAGCAGGCCTCTTCACTCAGTTCATTAAAGCGCTGGAC
XM_014129250.1      ------------------------------------------------------------


XM_014196267.1      TCTCTGAGTGGGAAGAACAAGTCATCTTCAGGCCCCCCGTCTCTGCCCATGGACTCAGTG
XM_014129250.1      ------------------------------------------------------------


XM_014196267.1      GCCCTCTCCCTGCAGGACCTCATCTTCTACTTCCGGCCCCCCGAGGAGGAGCTGGAGCAC
XM_014129250.1      ------------------------------------------------------------
```

```
XM_014196267.1      GAGGAGAAGCAGACCAAGCTACGCTCCCTCAAGAACAGACAGAACCTCTTCCAAGAGGAG
XM_014129250.1      ------------------------------------------------------------

XM_014196267.1      GGCATGATCACCCTGGTGCTGGACTGTATTGACCGACTCAATGTCTACAACACGGCGGCC
XM_014129250.1      ------------------------------------------------------------

XM_014196267.1      CACTTCTCAGAGTTTGCAGGGGAGGAAGCTGCCGAGTCTTGGAAGGAAATCGTTAATCTC
XM_014129250.1      ------------------------------------------------------------

XM_014196267.1      CTGTACGAGCTGCTGGCCTCTCTCATCAGAGGTAACCGGGCCAACTGTGCTTTGTTCTGT
XM_014129250.1      ------------------------------------------------------------

XM_014196267.1      GATAACCTGGATTGGCTGGTCAGCAAACTGGACCGTTTAGAGGCCTCCTCAGGCATCCTG
XM_014129250.1      ------------------------------------------------------------

XM_014196267.1      GAGGTGCTGTACTGCGTTCTGATTGAGAGTCCAGAGGTCCTGAACATCATCCAGGAGAAC
XM_014129250.1      ------------------------------------------------------------

XM_014196267.1      CACATTAAATCTATCATTTCTCTACTGGATAAGCATGGACGCAATCACAAGGTGTTGGAT
XM_014129250.1      ------------------------------------------------------------

XM_014196267.1      GTGCTCTGCTCTTTGTGTGTGTGTAACGGTGTGGCTGTAAGGTCCAATCAGAATCTCATC
XM_014129250.1      ------------------------------------------------------------

XM_014196267.1      ACAGAGAATCTGCTTCCCGGTCGCGACCTCCTCCTGCAGTCCAACATCATCAATTATGTC
XM_014129250.1      ------------------------------------------------------------

XM_014196267.1      ACCAGTGTGAGACCCAACATCTTCCTTGGAACCTGTGAGGGATCCACTCAGTATAAGAAG
XM_014129250.1      ------------------------------------------------------------

XM_014196267.1      TGGTATTTTGAGGTGATGGTGGATTATGTGGAGCCCTTCCTGACCGCCCAGGCCTTCCAC
XM_014129250.1      ------------------------------------------------------------

XM_014196267.1      CTGCGTGTGGGCTGGGCTCTGACAGAGGGCTACAGCCCGTACCCTGGGGGTGGTGAGGGC
XM_014129250.1      ------------------------------------------------------------

XM_014196267.1      TGGGGCGGCAACGGGGTTGGCGACGACCTTTACTCCTACGCCTTTGATGGACTCCACCTG
XM_014129250.1      ------------------------------------------------------------

XM_014196267.1      TGGTCAGGACGAGTCCTCCGCCATGTCGCCTCCCCCAACATGCACATCCTAGCGGCAGAC
XM_014129250.1      ------------------------------------------------------------

XM_014196267.1      GATGTCGTCAGCTGCTGCCTGGATCTGAGCGTGCCCAGTATCTCGTTCCGTATCAACGGG
XM_014129250.1      ------------------------------------------------------------

XM_014196267.1      CACCCGGTGCAGGGCATGTTTGAGAACTTCAACCTGGACGGCCTCTTCTTCCCTGTCGTC
XM_014129250.1      ------------------------------------------------------------

XM_014196267.1      AGCTTCTCTGCAGGCGTCAGGGTTCGTTTTCTCCTGGGAGGGCGTCATGGGGACTTCAAA
XM_014129250.1      ------------------------------------------------------------
```

```
XM_014196267.1      TTCCTCCCACCGCCAGGCTATGCACCGTGCTATGAGGCTGTGCTGCCCAAGGACCGACTG
XM_014129250.1      ------------------------------------------------------------

XM_014196267.1      CGTATTGAGCCCATCAAGGAGTATAAGCATGATTTCAATGGTGTCCGCAATCTGTTGGGG
XM_014129250.1      ------------------------------------------------------------

XM_014196267.1      CCTACACAGTCCCTCTCGCACACCGCCTTCACCCCCTGCCCTGTGGACACAGTACAGATT
XM_014129250.1      ------------------------------------------------------------

XM_014196267.1      GTGCTTCCGCCTCACTTGGAGCGCATTCGGGAGAAGCTGGCGGAAAACAGCCATGAGCTA
XM_014129250.1      ------------------------------------------------------------

XM_014196267.1      TGGGCCGCCACTCGCATTGAACAAGGATGGACCTACGGGTCGTTCCGAGATGACAACAAG
XM_014129250.1      ------------------------------------------------------------

XM_014196267.1      AAACTGCACCCCTGCCTGGTAGATTTCCAGAGCCTGCCAGAGCCAGAGAAGAATTACAAC
XM_014129250.1      ------------------------------------------------------------

XM_014196267.1      CTTGCAATGTCTGGAGAAACACTCAAGACTCTGCTGGCGCTGGGCTGTCACGTGGGCATG
XM_014129250.1      ------------------------------------------------------------

XM_014196267.1      GGGGATGAGAAAGCAGAGGAGAACCTGAAGAACATCAAGATGCCCAAAACGTATATGATG
XM_014129250.1      ------------------------------------------------------------

XM_014196267.1      AGTAGTGGATACAAGCCTGCCCCTCTGGACCTCAACCATGTCAAGCTGACACCCAACCAG
XM_014129250.1      ------------------------------------------------------------

XM_014196267.1      ACCAACCTAGTGGAGAGACTGGCAGAGAATGGGCACAACGTGTGGGCTCGTGACAGGGTC
XM_014129250.1      ------------------------------------------------------------

XM_014196267.1      CACCAGGGATGGACCTACAGCATTGTCCAGGACATTATGAGCAAGCGCAACCCGCGTCTG
XM_014129250.1      ------------------------------------------------------------

XM_014196267.1      GTGCCGTACAACCTGCTGGACGAAAAGACCAAGAAGACCAACAGAGACACTGTCTGTGCA
XM_014129250.1      ------------------------------------------------------------

XM_014196267.1      GCTGTCCGCACTCTCATCGGCTACGGTTACAACATTGAGCCACCTGACCAGGAGAGCAGT
XM_014129250.1      ------------------------------------------------------------

XM_014196267.1      GGTAATGGGGAAGGTCATTCCCGTGGAAATAAGATCCGGGTGTTTCGGGCTGAGAAGTCA
XM_014129250.1      ------------------------------------------------------------

XM_014196267.1      TACGCGGTGACCCAGGGGAAGTGGTACTTTGAGTTTGAGGCTGTCACCGTGGGGGATATG
XM_014129250.1      ------------------------------------------------------------

XM_014196267.1      AGAGTGGGTTGGGCCAGACCCAGTGTCCGCGCTGACACAGAGCTGGGGGCGGATGAGCTC
XM_014129250.1      ------------------------------------------------------------

XM_014196267.1      GCCTACGTCTTCAATGGTTTCAAGGCTCAGCGCTGGCATGTGGGCAATGAGCCGTTTGGT
XM_014129250.1      ------------------------------------------------------------
```

```
XM_014196267.1      CGTAGCTGGTTGCCAGGTGACGTGGTAGGCTGTATGATCGACCTGGTAGAGCAGAACATC
XM_014129250.1      ------------------------------------------------------------

XM_014196267.1      TTCTTCACCCTGAACGGAGAGATGCTGATCAGCGACTCTGGCTCTGAGATGGCCTTCAAG
XM_014129250.1      ------------------------------------------------------------

XM_014196267.1      GACATAGACACAGGAGATGGCTTTATCCCTGTGTGTAGTCTGGGCCTGTCTCAGGTTGGC
XM_014129250.1      ------------------------------------------------------------

XM_014196267.1      CGGCTCAACCTGGGCCAGAACGTCAGCAGCCTGCGCTACTTCACCATCTGTGGCCTGCAG
XM_014129250.1      ------------------------------------------------------------

XM_014196267.1      GAGGGCTTTGAGCCTTTCGCCATCAACATGAAGCGAGACATCACCATGTGGTTCAGCAAG
XM_014129250.1      ------------------------------------------------------------

XM_014196267.1      AGCCTGCCCCAGTTCATCCCTGTTCCTACAGAACACCCTCACATTGAGGTGTCCCGTGTG
XM_014129250.1      ------------------------------------------------------------

XM_014196267.1      GATGGGACAGTGGACAGCGCTCCGTGTCTGAAGCTGACCCATAAGACCTTTGGCTCTCAG
XM_014129250.1      ------------------------------------------------------------

XM_014196267.1      AACGCCAACACAGACCTGCTGTTCCTGAGGCTCAGCATGCCCGTGGAGTTCCACGAGACC
XM_014129250.1      ------------------------------------------------------------

XM_014196267.1      TTTAAGGTCATGGCCGGGACCACCCCCCTCACCCGAGCTCTGACCATCCCTGAGGACCAG
XM_014129250.1      ------------------------------------------------------------

XM_014196267.1      GTTCTGGAGGTTGACCCCGACTCTGACTTTGAGGTACTGAAGAAGTCTGCCAGCCGCACG
XM_014129250.1      ------------------------------------------------------------

XM_014196267.1      GAGAAGGAGGAGGAGAAGAAGGAGCCCTCTGTGCCTAAGGAGATCCCTGTCAACGAAGGA
XM_014129250.1      ------------------------------------------------------------

XM_014196267.1      GGAGAGAACGTGAAAGATGCCTCTACAGAGAAGAGCAAGAAGAAGGGGTTCCTGTTCAAG
XM_014129250.1      ------------------------------------------------------------

XM_014196267.1      GCCAAGAAGGCAGCTTTCACTCCACCCCCTGTTGTTCCCACCATGCCCCGCCTAATGGAG
XM_014129250.1      ------------------------------------------------------------

XM_014196267.1      GAAGTTGTACCAGACGATCGAGATGACGACGACATCATCCTCAACACCACCACATACTAT
XM_014129250.1      ------------------------------------------------------------

XM_014196267.1      TATTCAGTTCGAGTGATTGCGGGACAAGAACCCAGCGGTGTGTGGGTGGGCTGGATCACC
XM_014129250.1      ------------------------------------------------------------

XM_014196267.1      CCGGACTACCACCAGTACGACCTGCACTTTGACCTCAGCAAAGTCCGAAATGTCACTGTC
XM_014129250.1      ------------------------------------------------------------

XM_014196267.1      ACCGTGGGAGATGACAAAGGCAACATCCATGACAGTATGAAGCGCAGTAACTGCTACATG
XM_014129250.1      ------------------------------------------------------------
```

```
XM_014196267.1      GTGTGGGGAGGGGAGTTCAGCAGCTCCCAGCAGACCCGTGTCAGTCAGGAGGACTTTGTG
XM_014129250.1      ------------------------------------------------------------

XM_014196267.1      ATTGGCTGCCTTATTGACCTGGACACTGGCCTCATGACTTTCACAGCCAACGGGAAAGAG
XM_014129250.1      ------------------------------------------------------------

XM_014196267.1      ATCAACACCTTCTACCAGGTGGAGCCCAACACTAAACTGTTCCCAGCTGTCTTTGTCCTG
XM_014129250.1      ------------------------------------------------------------

XM_014196267.1      CCCTCCAGTCAGAACATGCTGCAAGTGGAGCTGGGCAAGCTCAAGAACATCATGCCCATC
XM_014129250.1      ------------------------------------------------ATCATGCCTATT
                                                                    ******** ** 

XM_014196267.1      TCGGCAGCCATGTTCCGCAGTGAGCGTAAGAACCCGGTCCCCCAGTGTCCTCCCAGGCTG
XM_014129250.1      TCAGCAGCCATGTTCCGCAGTGAGCGTAAGAACCCGGTCCCTCAGTGTCCTCCCAGGCTG
                    ** ************************************** ******************

XM_014196267.1      GACGTCCAGATGCTGACCCCAGTCATCTGGAGCCGTATGCCCAACCACTTCCTGTCCCCA
XM_014129250.1      GATGTCCAGATGCTGACCCCAGTCATCTGGAGCCGTATGCCCAACCACTTCCTGTCCCCA
                    ** *********************************************************

XM_014196267.1      GAGACGGGCCGTGTGAACGAGAGGCACGGCTGGATGGTGGAGTGTAGAGAGCCTCTCACC
XM_014129250.1      GAGACGGGCCGTGTGAACGACAGGCACGGTTGGATGGTGGAGTGTAGAGAGCCTCTCACC
                    ******************** ******** ******************************

XM_014196267.1      ATGATGGCCCTGCACATCCCTGAGGAGAACAGATGTATTGACGTCCTGGAGTTGTCGGAG
XM_014129250.1      ATGATGGCCCTGCACATCCCTGAGGAGAACAGATGTATTGACGTCCTGGAGTTGTCGGAG
                    ************************************************************

XM_014196267.1      CGTATGGACCTGCTGAAGTTCCACTACCACACTCTGAAGCTGTACGGCTCGGTCTGTGCT
XM_014129250.1      CGTATGGACCTGCTGAAGTTCCACTACCACACTCTGAAACTGTACGGCTCGGTCTGTGCT
                    ************************************** *********************

XM_014196267.1      CTGGGGAACAACCGCGTGGCTCACGCCCTATGCAGTCATGTTGATGAGTCCCAGCTGTTC
XM_014129250.1      CTGGGGAACAACCGCGTGGCTCACGCCCTGTGCAGCCATGTTGATGAGTCCCAGCTATTC
                    ***************************** ***** ******************** ***

XM_014196267.1      TACGCCATAGAGAACACCTACCTGCCTGGACCAATGAGGAGCGGCTACTACGACCTGCTC
XM_014129250.1      TACGCCATAGAGAACACCTACCTGCCTGGACCGATGAGGAGCGGCTACTACGATCTGCTC
                    ******************************** ******************** ******

XM_014196267.1      ATCAGCATGCACCTGGAGTCGGCCAAGAGGAACCGCCTCATGACCAACAAGGAGTTCATT
XM_014129250.1      ATCAGCATGCACCTGGAGTCGGCCAAGAGGAACCGCCTCATGACCAATAAGGAGTTCATT
                    *********************************************** ************

XM_014196267.1      GTGCCCATGACAGACGAGACGCGCAGCATCAACCTCTACTCAGACACTGACAACTCCCAT
XM_014129250.1      GTGCCCATGACTGAAGAGACGCGCAGCATCAACCTCAACTCAGACACTGACAAATCCCAT
                    *********** ** ********************* **************** ******

XM_014196267.1      GCTTTACCAGGGGTGGGCCTCACTACCTGCCTGCGCCCCAAACTCCACTTCTCCCCCACT
XM_014129250.1      GCTTTACCAGGGGTGGGCCTCACTACCTGTCTGCGCCCCAAACTCCACTTCTCCCCCACT
                    ***************************** ******************************

XM_014196267.1      GGCTTTGTGGGGACAGACCTGGACATCTACACCCTCAGCCCATTCATCCCCCTACAGGTG
XM_014129250.1      GGGTTTGTAGGGACAGACCTGGATATCTACACCCTCAGCCCCTTCATCCCCCTACATGTG
                    ** ***** ************** ***************** ************** ***

XM_014196267.1      CTGAAGGCGAAGGCCCTGACCATGCTGACAGAAGCTGTACAAGACGGTGGTCAGGCCATG
XM_014129250.1      CTGAAGGCGAAGGCCCTGACCATGCTGACCGAAGCTGTCCAGGATGGTGGTCAAGCCATG
                    ***************************** ******** ** ** ******** ******

XM_014196267.1      AGGGATCCTGTAGGAGGCAGTGTTGAGTTCCACTTTGTCCCCATCCTCAAGCTCATCAGC
XM_014129250.1      AGGGATCCTGTAGGAGGCAGTGTTGAGTTCCACTTTGTCCCCATCCTCAAGCTTATCAGC
                    ***************************************************** ******
```

```
XM_014196267.1      ACCCTGCTCATCATGGGCGTGTTCGAGAATGAAGATGTCAAGCACATCCTGAAGATGATA
XM_014129250.1      ACCCTGCTCATCATGGGTGTGTTTGAGAATGCAGATGTCAAGCACATCCTGAAGATGATT
                    ***************** ***** ******* *************************** 

XM_014196267.1      GAGCCCACGGTGTTCAGTGGCGAGGCAGAGGCTGCTGCAGAAGAAGCAGAAGCCACAGCC
XM_014129250.1      GAGCCCACAGTGTTCAGTGGAGAGGCAGAGGATGCTGC---------------------C
                    ******** *********** ********** ******                     *

XM_014196267.1      AAGGGCGCGGGGAGCCAAACGCTGGCGCTCAAAGAAGGGGGGGAGGAGGTGAAGGAGGAA
XM_014129250.1      CAGGGAGAGGGGGACCAAACACTGGCCCTCAAAGAACGGGGATTGGAGGTGAAGGAAGAA
                     **** * ****  ****** ***** ********* ****   ************ ***

XM_014196267.1      GAGGAGGGAGCAGTAGAGAGTGGACATGAAACAGAAATGGAGGACGAGGGGATGGGTGAG
XM_014129250.1      GAGAAGGGAGCAGTAGAGAGCGGACATGAAACAGAAATGGAGGATGAGGGGATGGGTGAG
                    *** **************** *********************** ***************

XM_014196267.1      GAAGATGAGGAACTGGAGGCAGAGCTGGAGGAGCCGGTAGAAAAAGAAGAAGAAGAGGAA
XM_014129250.1      GAAGATGAGGAAATGGAGGCAGAGCTGGAGGAGTTGG---------------AGCCTATA
                    ************ ********************  **               *      *

XM_014196267.1      GAAGAGGAGGAGGATGGAGAGAAAGTGGATGGAGAGAAAGGTGCTGAGGAGACGGAGAAG
XM_014129250.1      GAAAAAGAGGAAGACGGAGAGAAAGTGGATGGAGAGAAAGGTGCTGAGGAGACGGAGAAG
                    *** * ***** ** *********************************************

XM_014196267.1      GAGGCGACGCCTGGAGAGGCAGACGGAGAGGCAGAAGAACAAGTAGGTCTGGAAGAGGGA
XM_014129250.1      GAGGTGAAGCCTGGAGAGGCAGG------------AGAACAAGTAGGTCTGGAAGAGGGA
                    **** ** **************             *************************

XM_014196267.1      TTACTGCACATGAAGCTGCCAGAGTCTGTCAAGCTACAGATGTGCACTCTGCTTCAGTAT
XM_014129250.1      TTACTGCACATGAAGCTGCCAGAGTCTGTCAAGCTACAGATGTGCACTCTGCTGCAGTAT
                    ***************************************************** ******

XM_014196267.1      TTCTGTGACTGTGAGTTGCGCCACAGGGTGGAGGCCATCATAGCCTTCTCAGACCAGTTT
XM_014129250.1      TTCTGTGACTGTGAGTTGCGCCACAGGGTGGAGGCAATCATAGCCTTCTCAGACGAGTTT
                    *********************************** ****************** *****

XM_014196267.1      GTGAGCCAGGTGCAGGCCAACCAGAGAGCCCGCTACAACGAGCTCATGCTGGCGTTCACC
XM_014129250.1      GTGAGCCAGGTGCAGGCCAACCAGAGAGCCCGCTACAATGAGCTCATGCTGGCGTACACC
                    ************************************** **************** ****

XM_014196267.1      ATGAGCGCTGCCGAGACTGCCCGCAAGACCCGCGAGTTCCGCTCCCCACCGCAGGAGCAG
XM_014129250.1      ATGAGCGCTGCTGAGACCGCTCGCAAGACCCGCGAGTTCCGCTCCCCACCACAGGAGCAG
                    *********** ***** ** ***************************** *********

XM_014196267.1      GTCAACATGCTGATGAACTTTAAGAGCATTGCGGAGGATGAGGAGTGTCCTGTGCCTGAC
XM_014129250.1      GTCAACATGCTGATGAACTTGAAGAACATTGCGGAGGATGAGGATTGTCCTGTGCCTGAC
                    ******************** **** ****************** ***************

XM_014196267.1      GAGGTCCGCGATGCCCTGCTGTCTTTTCACAAGAACCTGCTTTCTCACTGCGGTGTACAC
XM_014129250.1      GAGGTCCGCGATGGCCTGCAGGCTTTTCACAAGAACCTGCTGTTTCACTGCAGTGTACAT
                    ************* ***** * ******************* * ******* ******* 

XM_014196267.1      ATTGAAGAGGAAGAGGTGGAGGAGGAGTTGGATATGTCTCTCAAAGGACGACTCTTCAGA
XM_014129250.1      ATCGAAGAGGAAGAGGTAGAGGAGGAGTTGGATATGTCTCTCAAAGGACGACTCCTCCGA
                    ** ************** ************************************ ** **

XM_014196267.1      ATGTTGGACAAGTTGAGGCACCTCCGCAAGAAGAAGGTAGAGGAGGAGCCGGAACCTGTA
XM_014129250.1      ATGCTGGACAAGTGGAGGCACCTCCGTAAGAAGAAGGTAGAGGAGGAG---GAACCTGAG
                    *** ********* ************ *********************   *******  

XM_014196267.1      GAAGAGACCAAACCCAGCACCCTCCAGGAGCTAATCTCCCACACTATGATCCACTGGGCC
XM_014129250.1      GAGGAGACCAAACCCAGCACCCTCCAGGAGCTGATCTCCCACACCATGGTCCACTGGGCC
                    ** ***************************** *********** *** ***********

XM_014196267.1      CAGGAGTCGTTCATTCAGAACCCTGAGCTGGTGCGTCTGATGTTCAGCCTGCTGCACCGT
XM_014129250.1      CAGGAGTCATTCATCCAGAACCCTGAGCTGGTGCGTCTGATGTTCAGCCTGCTGCACCGT
                    ******** ***** *********************************************
```

```
XM_014196267.1      CAGTATGACGGGCTGGGGGAGCTGATCCGGGCCCTGCCCAAGGCCTACACCATCAACGCC
XM_014129250.1      CAGTATGACGGGCTGGGGGAGCTGATCCGGGCCCTGCCCAAAGCCTACACCATCAATGCC
                    ***************************************** ************** ***

XM_014196267.1      ATCTCCATAAAGGATACCATGGACCTGCTGGAGTGCCTGGGACAGATCCGCTCGCTGCTC
XM_014129250.1      ATCTCCATAAAGGATACCATGGACCTGCTGGAGTGCCTGGGACAGATCCGCACGCTGCTC
                    *************************************************** ********

XM_014196267.1      ATTGTCCAGATGGGCCCCGAGGAGGAGAGACTCATGATCCAGAGCATCGGAAACATCATG
XM_014129250.1      ATCGTCCAGATGGGCCCCGAGGAGGAGAGGCTCATGATCCAGAGCATCGGGAACATCATG
                    ** ************************** ******************** *********

XM_014196267.1      AGCAACAAAGTGTTTTATCAGCACCCCAACCTGATGCGAGCGCTGGGCATGCACGAGACT
XM_014129250.1      AGCAACAAAGTGTTTTATCAGCACCCCAACCTGATGCGAGCGCTGGGCATGCACGAGACT
                    ************************************************************

XM_014196267.1      GTCATGGAGGTGATGGTCAACGTGCTGGGTGGTGGCGACTCCAAGGAGATCAGATTCCCT
XM_014129250.1      GTCATGGAGGTGATGGTCAACGTGCTGGGTGGTGGCGACTCCAAGGAGATCAGATTCCCT
                    ************************************************************

XM_014196267.1      CGTATGGTCACCAACTGCTGTCGTTTCCTGTGCTACTTCTGTCGTATCAGTCGCCAGAAC
XM_014129250.1      CTTATGGTCACCAACTGCTGTCGTTTCCTGTGCTACTTCTGTCGTATCAGTCGTCAGAAC
                    * *************************************************** ******

XM_014196267.1      CAGCGTTCCATGTTCGACCACCTCAGCTACCTGCTACAGAACAGTGGCATTGGCCTTGGA
XM_014129250.1      CAACGCTCCATGTTCGATCACCTCAGCTACCTGCTTCAGAACAGTGGCATCGGCCTTGGA
                    ** ** *********** ***************** ************** *********

XM_014196267.1      ATGCGTGGCTCCACCCCTCTAGACGTGGCTGCAGCATCCTGCATTGACAACAATGAGCTG
XM_014129250.1      ATGCGTGGCTCCACCCCTCTAGACGTAGCTGCAGCATCCTGTATTGATAACAATGAGCTG
                    ************************** ************* ****** ************

XM_014196267.1      GCTCTGGCTCTACAGGAACAAGACCTGGAGAAGGTGGTGAAGTACCTCGCTGGCTGTGGG
XM_014129250.1      GCTCTGGCTCTACAGGAACAAGACCTGGAGAAGGTGGTGAAGTACCTTGCAGGCTGTGGG
                    *********************************************** ** *********

XM_014196267.1      CTCCAGAGCTGTCCCCAGCTCCTGGCTAAGGGTTACCCTGACATCGGCTGGAACCCGTGT
XM_014129250.1      CTTCAGAGCTGTCCCCAGCTCCTGGCTAAGGGTTACCCTGACATTGGCTGGAACCCTTGT
                    ** ***************************************** *********** ***

XM_014196267.1      GGCGGAGAGAAATACCTGGACTTCCTCCGCTTCGCTGTCTTCGTCAATGGAGAGAGCGTA
XM_014129250.1      GGCGGAGAGAAATACTTGGACTTCCTCCGCTTCGCTGTCTTTGTCAACGGAGAGAGCGTA
                    *************** ************************* ***** ************

XM_014196267.1      GAGGAGAATGCCAACGTGGTGGTGCGTCTTCTGATCCGTCGGCCAGAGTGTTTTGGCCCG
XM_014129250.1      GAGGAGAATGCCAATGTGGTGGTGCGTCTTCTGATCCGTCGGCCAGAGTGTTTTGGCCCG
                    ************** *********************************************

XM_014196267.1      GCCCTGAGAGGAGAGGGTGGGAACGGCCTGCTGGCTGCTATAGAGGAGGCCATCAAGATC
XM_014129250.1      GCTCTGAGAGGAGAGGGTGGGAACGGCCTGCTGGCTGCTATAGAGGAGGCCATCAAGATC
                    ** *********************************************************

XM_014196267.1      TCTGAAGATCCTGCCAGGGACGGCCCCACTGTGAAGAAAGACAGGCGCTTCCCCGGCATG
XM_014129250.1      TCTGAGGATCCTGCCAGGGACGGCCCCACTGTCAAGAAAGACAGGCGCTTCCCCGGCATG
                    ***** ************************** ***************************

XM_014196267.1      TTCCCTGGTGGGGAGGAGCAGCACGAGGAGAACAAGGTTCACCTGGGGAATGCCATCATG
XM_014129250.1      TTCCCTGGTGGAGAAGGGCAGCATGAGGAGAACAAGGTGCATCTTGGGAATGCCATCATG
                    *********** ** * ****** ************** ** ** ***************

XM_014196267.1      TCCTTTTACTCAGCTCTCATTGACTTGCTGGGACGCTGCGCTCCAGAGATGCATTTGATC
XM_014129250.1      TCCTTCTACTCAGCTCTCATTGACTTGCTGGGACGCTGCGCTCCAGAGATGCATTTAATC
                    ***** ************************************************** ***

XM_014196267.1      CAGGCTGGGAAGGGCGAGGCTCTGAGGATCAGGGCCATCCTCAGGTCCCTGGTGCCTATG
XM_014129250.1      CAGGCTGGGAAGGGTGAGGCTCTGAGGATCAGGGCCATCCTGAGGTCCCTGGTGCCCATA
                    ************** ************************** ************** ** 
```

```
XM_014196267.1      GAGGACCTGGTGGGAGTCATCAGCCTGTCTGTCCAGATACCAGACTTTGGAAAAGATAAC
XM_014129250.1      GAGGACCTGGTGGGAGTCATCAGCCTGTCTGTCCAAATCCCAGACTTTGGAAAGGATAAC
                    *********************************** ** ************** ******

XM_014196267.1      AGCGTCATTGAACCCAAGATGTCTTCCAGTTTTGTGCCGGACCACAAGGCTCCCATGGTG
XM_014129250.1      AGCATCATTGAACCCAAGATGTCTTCCAGTTTTGTGCCGGACCACAAGGCTCCCATGGTG
                    *** ********************************************************

XM_014196267.1      CTGTTCCTTGACAGAGTGTATGGTATTGACAACCAAGACTTCCTGCTTCATGTGCTGGAG
XM_014129250.1      CTGTTCCTTGACAGAGTGTATGGTATTGACAACCAGGACTTCCTGCTCCATGTGCTGGAG
                    *********************************** *********** ************

XM_014196267.1      GTGGGCTTCCTGCCTGACATGAGGGCAGCTGCTTCTCTGGACACAGCGGCTTTCTGCACC
XM_014129250.1      GTGGGCTTCCTGCCTGACATGAGGGCAGCTGCTTCTCTGGACACAGCGGCTTTCTGCACC
                    ************************************************************

XM_014196267.1      ACAGAGATGGCCCTGGCTCTGAACCGCTACCTCTCTCTGGCCGTGCTGCCCCTCATCACC
XM_014129250.1      ACAGAGATGGCCCTGGCTCTGAACCGCTACCTCTCTCTGGCCGTGCTGCCCCTCATCACC
                    ************************************************************

XM_014196267.1      AAGTGTGCCTTTTTGTTCGCTGGCACTGACCACCGGGCCATCATGATCGACTCTATGCTG
XM_014129250.1      AAGTGTGCCTTCCTGTTTGCCGGCACAGACCACCGGGCCATCATGATCGACTCAATGCTG
                    ***********  **** ** ***** ************************** ******

XM_014196267.1      CACACCATCTACCGTCTGTCCCGCGGACGAGCCTTCACTAAGGCCCAGAGAGATGTTATC
XM_014129250.1      CACACCATCTACCGTCTGTCCCGCGGACGAGCCTTCACTAAGGCCCAGAGAGATGTTATT
                    *********************************************************** 

XM_014196267.1      GAGGAGTGTCTCATGGCTTTGTGCAAGAATCTGCGGCCATCCATGTTACAACACCTGTTG
XM_014129250.1      GAGGAGTGTCTCATGGCTTTGTGCAAAAATCTACGGCCATCCATGTTACAACACCTGTTG
                    ************************** ***** ***************************

XM_014196267.1      AGAAGGCTGGTATTTGATGTGCCAATTCTCAATGAATATGCCAAAATGCCACTCAAGCTT
XM_014129250.1      AGAAGGCTGGTGTTTGATGTACCAATTCTCAATGAATATGCCAAAATGCCACTTAAGCTT
                    *********** ******** ******************************** ******

XM_014196267.1      TTGACCAATCACTATGAGCGTTGTTGGAAGTACTATTGCCTGCCCAACGGCTGGGGCAAC
XM_014129250.1      TTGACCAATCACTATGAGCGTTGTTGGAAGTACTATTGCCTGCCCAACGGCTGGGGCAAC
                    ************************************************************

XM_014196267.1      TTTGGAGTGTCATCAGAGGAGGAGCTGCATCTCACCCGTAAACTCTTCTGGGGCATCTTT
XM_014129250.1      TTTGGAGTGTCATCAGAGGAGGAGCTGCATCTCACCCGCAAACTCTTCTGGGGCATCTTT
                    ************************************** *********************

XM_014196267.1      GAGTCCCTGGCCCACAAGAAATTTGATGCTGAGCTGTTCAAAATTGCCATGCCGTGCATA
XM_014129250.1      GAGTCCCTGGCCCACAAGAAATTTGATGCTGAGCTGTTCAAAATTGCCATGCCGTGCATA
                    ************************************************************

XM_014196267.1      TGCGCCATTGCTGGCGCCATCCCTCCAGATTATGTAGATGCAAGCTACTCATCCAAGACT
XM_014129250.1      TGTGCCATTGCTGGTGCCATTCCTCCAGATTATGTAGATGCCAGCTACTCCTCCAAGACT
                    ** *********** ***** ******************** ******** *********

XM_014196267.1      GAGAAGAAAGCTTTGGTGGATGCAGAGGGAAACTTTGATCCCAAACCAGTGGAGACCACA
XM_014129250.1      GAGAAGAAAGCCTCGGTGGATGCAGAGGGAAACTTTGATCCCAAACCAGTGGAGACCACA
                    *********** * **********************************************

XM_014196267.1      AATACTATCATCCCTGAGAGATTGGATGGCTTCATCAACAGATATGCTGAGTACACACAT
XM_014129250.1      AATACTATCATCCCTGAGAGATTGGATGGTTTCATCAACAAATATGCTGAATACACACAT
                    ***************************** ********** ********* *********

XM_014196267.1      GACAAGTGGGCCTTTGAAAAGATTCAGAATAACTGGACGTATGGAGAGATGTTGGATGAA
XM_014129250.1      GACAAGTGGGCTTTTGAAAAGATTCAGAATAACTGGACGTATGGAGAGATGTTGGATGAA
                    *********** ************************************************

XM_014196267.1      AATTCAAAAACTCACCCCATGCTCCGACCGTACAAAACGTTCTCCGAGAAGGACAAAGAG
XM_014129250.1      GATTCTAAAACTCACCCCATGCTCCGACCGTACAAAACATTCTCAGAGAAGGACAAAGAG
                     **** ******************************** ***** ***************
```

```
XM_014196267.1      ATCTACCGTTGGCCCATCAAAGAGTCCATGAAGGCCATGATTGCATGGGAGTGGACTCTG
XM_014129250.1      ATCTACCGTTGGCCCATCAAAGAGTCCATGAAGGCTATAATTGCGTGGGAGTGGACTCTG
                    *********************************** ** ***** ***************

XM_014196267.1      GATCAAACGAGGGAAGGAGATGAAGCCAAGGCTGAGCTGAAGAAGGCAGCTCGGAAGATC
XM_014129250.1      GATCAAACGAGGGAAGGAGAGGAAGCCAAGACTGAGCAAAAGAAGGCGGCTCGGAAAATC
                    ******************** ********* ******  ******** ******** ***

XM_014196267.1      TCTCAGACTGCGCAGGCAACATATGACCCCAGCCATGGCTACAGTCCCCAACCAATTGAA
XM_014129250.1      TCTCAGACTGCACAGGCAACGTACGACCCCAGCCAAGGCTACAGTCCCCAACCGATTGAA
                    *********** ******** ** *********** ***************** ******

XM_014196267.1      ATATCCCACATGGCACTGTCAAGAGAGCTGCAGTCAATGGCAGAACAACTTGCAGAGAAC
XM_014129250.1      ATCTCCCACATGGCACTGTCAAGAGAGCTGCAGTCCATGGCAGAACAGCTTGCAGAGAAC
                    ** ******************************** *********** ************

XM_014196267.1      TATCACAACACCTGGGGTCGAAAGAAGAAGATGGAGCTGCAGTCCAAAGGAGGAGGCGCC
XM_014129250.1      TATCACAATACCTGGGGTCGCAAGAAGAAGATGGAGCTGCAGTCCAAAGGAGGAGGCTCC
                    ******** *********** ************************************ **

XM_014196267.1      CACCCTTTGCTTGTACCCTATGACACCCTGACAGCAAAGGAGAAGGCACGAGACAGGGAA
XM_014129250.1      CACCCTTTGCTTGTACCCTACGACACCCTGACAGCAAAGGAAAAGGCACGAGACAGGGAA
                    ******************** ******************** ******************

XM_014196267.1      AAGGCTCACGAGCTTCTCAAATTTCTTCAACTCAATGGATTTGCAGTCACCAGGGGAATG
XM_014129250.1      AAGGCTCACGAGCTTCTTAAATTCCTTCAACTCAATGGATTTGCAGTCACCAGGGGGATG
                    ***************** ***** ******************************** ***

XM_014196267.1      AAAGACATGGAGTCAGACATCTCATCTATCGAGAAGCGCTTTGCCTATGGTTTCCTGCAG
XM_014129250.1      AAAGACATGGAGTCAGACATCTCATCTATCGAGAAGCGCTTTGCTTATGGTTTCCTGCAG
                    ******************************************** ***************

XM_014196267.1      AAGCTGTTGAAGTGGATGGAGATCGCCCAAGAGTTCATAGCTCATCTTGAGGCTGTGGTG
XM_014129250.1      AAGTTGTTAAAGTGGATGGAGATCGCCCAGGAGTTCATAGCTCATCTTGAGGCTGTGGTG
                    *** **** ******************** ******************************

XM_014196267.1      AGCAGTGGCAGAGTGGAGAAGTCGCCTCATGAGCAGGAGATCAAATTCTTTGCCAAGATC
XM_014129250.1      AGCAGTGGCAGAGTGGAGAAGTCGCCTCATGAGCAGGAGATCAAATTCTTTGCAAAGATC
                    ***************************************************** ******

XM_014196267.1      TTGTTGCCCCTGATCAACCAGTACTTTAAAAACCACTGTCTGTACTTCCTGTCCACACCG
XM_014129250.1      CTGTTGCCCCTGATCAATCAGTACTTTAAAAACCACTGTCTGTACTTCCTGTCCACACCG
                     **************** ******************************************

XM_014196267.1      GCCAAAGTCCTGGGTAGTGGAGGACATTCCTCTAACAAGGAGAAAGAGATGATTGCCAGT
XM_014129250.1      GCCAAAGTCCTGGGTAGTGGAGGACATTCCTCTAACAAGGAGAAAGAGATGATTGCCAGC
                    ***********************************************************

XM_014196267.1      ATCTTCTGTAAAATGGCTGCTCTGGTGAGACACAGAGTTTCTCTCTTCGGGAACGACGCT
XM_014129250.1      ATCTTCTGTAAAATGGCTGCTCTGGTGAGACACAGAGTTTCTCTCTTTGGAAATGACGCC
                    *********************************************** ** ** *****

XM_014196267.1      GCTGCTATTGTCAACTGTCTTCACATCCTGGCACGATCACTCGATGCAAGGACGGTGATG
XM_014129250.1      CCTGCTATTGTCAACTGTCTTCACATCCTGGCACGATCTCTCGACGCAAGGACGGTGATG
                     ************************************* ***** ***************

XM_014196267.1      AAGTCTGGGCCTGAGATTGTGAAGGCAGGGCTCCGGTCGTTCTTTGAGGGTGCAGCTGAT
XM_014129250.1      AAGTCTGGGCCTGAAATCGTGAAGGCAGGGCTCAGGTCATTCTTTGAGGGTGCAGCTGAT
                    ************** ** *************** **** *********************

XM_014196267.1      GATATCGAGAAGATGGTGGAGAACCTCAAACTGGGAAAGGTGTCTAAAGGCAACCAGCAG
XM_014129250.1      GATATAGAGAAGATGGTGGAGAACCTCAAACTGGGAAAGGTGTCTAAAGGCAACCAGCAG
                    ***** ******************************************************

XM_014196267.1      GTGAAAGGCGTGTCCCAGAACATCAACTACACCACCATTGCTCTGCTCCCAGTCCTCACC
XM_014129250.1      GTGAAAGGCGTGTCCCAGAACATCAACTACACCACCATTGCTCTGCTCCCAGTCCTCACC
                    ************************************************************
```

```
XM_014196267.1      TCCCTGTTCGACCACATCTCTCAGCACCAGTTTGGAGATGATGTCATGCTGGATGATCTC
XM_014129250.1      TCCCTGTTCGACCACATCTCTCAGCACCAGTTTGGAGATGATGTCATTTTGGATGATCTC
                    ***********************************************  ***********

XM_014196267.1      CAGATGTCGTGTTACCGCATCATGTGCGCCATCTACTCCCTGGGCAGTGTCAAGAACCCT
XM_014129250.1      CAGATGTCGTGTTACCGCATCATGTGCGCCATCTACTCGTTGGGTACTGTCAAGAACCCT
                    **************************************  **** * *************

XM_014196267.1      CATGTGGAGAGGCAGAGGCCAGCCCTGGGGGAGTGTCTAGCCCATCTGGCGGCTGCTATG
XM_014129250.1      CATGTGGAGAGGCAGAGGCCAGCCCTGGGGGAGTGTCTGGCCCATCTGGCGGCTGCTATG
                    ************************************** *********************

XM_014196267.1      CCTGTGGCCTACCTCGAGCCCCATCTCAATGAGTACAATGCCTTCTCTGTCTACACCACC
XM_014129250.1      CCTGTGGCCTACCTGGAGCCCCATCTCAATGAGTACAACTCCTTCTCTGTTTACACCACC
                    ************** ***********************  ********** *********

XM_014196267.1      AAGACCCCCAGAGAAAGAGCCATCTTGGGTCTGCCCAACGAGGTCCAGGAGTTATGCCAG
XM_014129250.1      AAGACCCCCAGAGAAAGAGGCATCCTGGGTCTGCCCAATGAGGTCCAGGAGTTATGCCAG
                    ******************* **** ************* *********************

XM_014196267.1      GACATACCAGAGCTAGACGTTCTGCTGAAAGAGATCGGGGACTTGGCAGAGTCAGGTGCC
XM_014129250.1      GACATACCAGAGCTAGACGTTCTATTGAAAGAGATCGGGGACTTGGCAGAGTCAGGTGCC
                    ***********************  ***********************************

XM_014196267.1      CGTTACACTGAGATGCCCCATGTGATCGAGATCACTCTGCCCATGCTGTGTAACTACCTG
XM_014129250.1      CGTTACACAGAGATGCCCCATGTAATCGAGATAACTCTGCCCATGCTGTGTAACTACCTG
                    ******** ************** ******** ***************************

XM_014196267.1      CCTCGCTGGTGGGAGAGAGGAGTAGAGAACTTCCCTGAGCTGGAGGGCCAAATCTGCACC
XM_014129250.1      CCCCGCTGGTGGGAAAGAGGAGTAGAGAACTTCCCTGAGCTGGAGGGCCAGATCTGCACC
                    ** *********** *********************************** *********

XM_014196267.1      GACGTCACCTCTGACCAGCTCAACCAGCTGCTGGGCAGCATCATGAAGATCGTGGTCAAC
XM_014129250.1      GACGTCACCTCTGAGCAGCTCAACCAGCTGCTGGGCAGCATCATGAAGATTGTCGTCAAC
                    ************** *********************************** ** ******

XM_014196267.1      AACCTGGGCATTGACGAGGCCTCCTGGATGAAGAGGCTGGCTGTCTTCTCCCAGCCTATT
XM_014129250.1      AACCTGGGAATTGACGAGGCCTCCTGGATGAAGAGGCTGGCTGTCTTCTCCCAGCCTATT
                    ******** ***************************************************

XM_014196267.1      GTGAGCAGGGCCAGGCCAGAGATGCTCAAGTCCCATTTCATCCCCACCATGGAGAAGCTG
XM_014129250.1      GTGAGCAGGGCCAAGCCAGAGATGCTCAAGTCCCATTTCATCCCCACCATGGAGAAGCTG
                    ************* **********************************************

XM_014196267.1      AAGAAGAGGACAGGGAAGGTGGTGGCCGAGGAGGACCACCTGCGTATGGAGGGGAAGGCC
XM_014129250.1      AAGAAGAGGACAGGGAAGGTGGTGGCAGAGGAGGACCACCTCCGTATGGAGGGGAAGGCC
                    ************************** ************** ******************

XM_014196267.1      GAGGGGGACGAGGAGGAGGGCACCATCAGGGATGAGTTTGCTGTGCTCTGCAGGGACCTA
XM_014129250.1      GAGGGGGACGAGGAGGAGGGCACCATCAGGGAGGAGTTTGCTGTGCTCTGCAGGGACCTG
                    ******************************** **************************

XM_014196267.1      TACGCCCTCTACCCTCTCCTCATCCGCTATGTGGACAATAACAGGGCGAGGTGGTTGACA
XM_014129250.1      TACGCCCTCTACCCTCTCCTCATCCGCTATGTGGACAATAACAGGGCAAGGTGGTTGACA
                    *********************************************** ************

XM_014196267.1      TGCCCGGACCCAGATGCAGAGGAGCTCTTCAGAATGGTTGGAGAGGTCTTCATCTTCTGG
XM_014129250.1      TGCCCAGACCCAGATGCAGAGGAGCTCTTCAGAATGGTTGGAGAGGTCTTCATATTCTGG
                    ***** *********************************************** ******

XM_014196267.1      TCCAAATCTCATAACTTCAAGCGAGAGGAGCAGAACTTTGTGGTGATGAATGAGATTAAT
XM_014129250.1      TCCAAATCTCATAACTTCAAGCGAGAGGAGCAGAACTTTGTGGTGATGAATGAGATTAAT
                    ************************************************************

XM_014196267.1      AACATGTCTTTCCTCACTGCTGACAGCAAGAGCAAGATGAGCAAGGGCAGCGACTCAGAG
XM_014129250.1      AACATGTCTTTCCTCACTGCTGACAGCAAGAGCAAGATGAGCACGGGCAACGACTCAGAG
                    ******************************************* ***** **********
```

```
XM_014196267.1      GCTGGTGGCTCAGACGTGGAGCGTACCAAGAAGAAGAGACGGGGAGACCGTTACTCGGTG
XM_014129250.1      GCTGGTGGCTCAGATGTGGAGCGTACCAAGAAGAAAAGACGAGGAGATCGTTATTCAGTG
                    ************** ******************** ***** ***** ***** ** ***

XM_014196267.1      CAGACGTCACTTATTGTGGCTGCCCTGAAAAAGATGCTCCCCATCGGCCTCAACATGTGC
XM_014129250.1      CAGACGTCACTTATTGTGGCTGCCCTGAAAAAGATGCTCCCCATCGGCCTCAACATGTGC
                    ************************************************************

XM_014196267.1      TCCCCTGCTGATCATGAGCTTATCAACCTGGCCAAGATCCGATACTCGTTGAGGGACACT
XM_014129250.1      TCCCCTGCTGATCAGGAGCTTATCAACCTGGCCAAGATACGATACTCTTTGAGGGACACG
                    ************** *********************** ******** *********** 

XM_014196267.1      GATGAGGAAGTAAGGGAGTTCTTGCAAAACAACCTGCATCTTCAAGGCAAGGTGGAGAAC
XM_014129250.1      GATGAGGAAGTAAGGGAGTTTTTGCAAAACAACCTGCATCTTCAAGGCAAGGTGGAGAAC
                    ******************** ***************************************

XM_014196267.1      CCGTCCATGCGCTGGCAGATGTCCCTGTATAAGGAGATGGCAGGGAAGGCTGAGGATGCT
XM_014129250.1      CCGTCCATGCGCTGGCAGATGTCCCTGTATAAGGAGATGGCAGGGAAGGCTGAGAATGCT
                    ****************************************************** *****

XM_014196267.1      GACGCTCCAGAGAAAGTGGTGAAGAGGGTGCAGGAGGTGTCAGCTGTGCTCTATCACATT
XM_014129250.1      GACGCTCCAGAGAAGGTGGTGAAGAGGGTGCAGGAGGTGTCAGCTGTTCTCTATTACATT
                    ************** ******************************** ****** *****

XM_014196267.1      GAGGTGACGGAGCACCCCTTCAAATCCAAAAAGATGGTGTGGCACAAGCTGCTGTCCAAG
XM_014129250.1      GAGGTGACGGAGCACCCCTTCAAATCCAAAAAGATGGTGTGGCACAAACTGCTGTCCAAG
                    *********************************************** ************

XM_014196267.1      CAGAGACGCAAGGCTGTGGTAGCCTGCTTTAGGATGACGCCACTCTACAACCTACCCAGG
XM_014129250.1      CAGAGACGCAAGGCTGTGGTAGCCTGCTTTAGGATGACTCCACTCTACAACCTACCCAGG
                    ************************************** *********************

XM_014196267.1      CATAGAGCTTCCAATATGTTCTTGGAAGGGTACAAACGCAACTGGATCCATACTGAGGGC
XM_014129250.1      CATAGAGCTTCCAATTTGTTCCTGGAAGGATACAAACGCAACTGGATCCATACTGAGGGC
                    *************** ***** ******* ******************************

XM_014196267.1      TACTCCTTTGAGGACAGAATGATAGACGACTTGTCTAAAGCCATGGAGCAGGAAGGAGAG
XM_014129250.1      TACTCCTTTGAGGACAGAATGATAGACGACTTGTCTAAAGCCATGGAGCAGGAAGGAGAG
                    ************************************************************

XM_014196267.1      GAGGAGGAAGAGACGGAGACCAAGCCAGACCCCCTTCACCAGCTCATTTTGCATTTCAGC
XM_014129250.1      GAAGAGGAAGAGACGGAGACCAAGCCAGATCCCCTTCACCAACTCATTTTGCATTTCAGC
                    ** ************************** *********** ******************

XM_014196267.1      CGTACTGCTCTCACAGAAAAGAGTAAACTTGATACAGATTACCTATATATGGCATATGCT
XM_014129250.1      CGTACCGCTCTCACAGAAAAGAGTAAACTTGATACAGATTACCTATATATGGCATACGCT
                    ***** ************************************************** ***

XM_014196267.1      GATATTATGGCAAAGAGCTGCCACATTGGTGAGGAGGACGAAGGGGGAGAGGAGGTGGAG
XM_014129250.1      GATATTATGGCAAAGAGCTGCCACATTGGTGAGGAGGACGAAGGGGGAGAGGAGGTGGAG
                    ************************************************************

XM_014196267.1      GAAGGGGCTGAGGATGAGATGTCCTTTGAGGTGCGACAGACAGAACTGGAAAAAGAGATG
XM_014129250.1      GAAGGGGCTGAGGATGAGATGTCCTTTGAGATGCGACAGACAGAACTGGAAAAAGAGATG
                    ****************************** *****************************

XM_014196267.1      GAGAAGCAGAGGCTCCTATACCAGCAGTCCCGTCTCCACAACCGTGGGGCGGCTGAGATG
XM_014129250.1      GAGAAACAGAGACTTCTGTACCAGCAGTCCCGTCTCCATAACCGTGGGGCGGCTGAGATG
                    ***** ***** ** ** ******************** *********************

XM_014196267.1      GTGCTGCAGATGATCAGCGCTTGCAAAGGTGAGACTGGCTGCATGGTGTCCTCCACTCTT
XM_014129250.1      GTGCTGCAGATGATCAGTGCTTGCAAAGGTGAGACTGGCTGTATGGTGTCCTCCACCCTT
                    ***************** *********************** ************** ***

XM_014196267.1      AAACTGGGCATCTCCATCCTAAACGGAGGTAACGTTGAGGTTCAACAGAAAATGCTTGAA
XM_014129250.1      AAACTGGGCATCTCCATCCTAAACGGTGGTAACGTTGAGGTTCAACAGAAAATGCTTGAG
                    ************************** ******************************** 
```

```
XM_014196267.1      TACCTGAAGGATAAGAAGGACGTTGGCTTCTTTTTGAGTGTCCAGGCTCTAATGCAGACG
XM_014129250.1      TACCTGAAGGACAAGAAGGATGTTGGCTTCTTTTTGAGTGTCCAGGCTCTAATGCAGACA
                    *********** ******** ************************************** 

XM_014196267.1      TGCAGTGTCCTGGACCTGAATGCCTTTGAGAGACAGAACAAGGCAGAGGGGCTGGGTATG
XM_014129250.1      TGCAGTGTCCTGGACCTGAATGCCTTTGAGAGACAGAACAAGGCAGAGGGTCTGGGCATG
                    ************************************************** ***** ***

XM_014196267.1      GTGTCGGAGGAGGGAACAAACATGAAGTCAGAACGGGGTGAGAAAGTCATGGCGGATGAT
XM_014129250.1      TTGTCGGAGGAGGGAACAAACATGAAGTCAGAACGGGGTGAAAAAGTCATGTCAGATGAT
                     **************************************** ********* * ******

XM_014196267.1      GAGTTTACCTGCGACCTGTTCCGCATGTTGCAGCTGCTCTGTGAGGGCCACAATAACGAT
XM_014129250.1      GAGTTTACATGTGATCTGTTCCGCATGTTGCAGCTGCTCTGTGAGGGCCACAATGACGAT
                    ******** ** ** *************************************** *****

XM_014196267.1      TTCCAGAACTATTTGCGGACACAGACAGGCAGCACCACCACTATAAACATCATCATCTGC
XM_014129250.1      TTCCAGAACTACTTGCGGACACAGACAGGCAGCACCACCACTATCAACATCATCATCTGC
                    *********** ******************************** ***************

XM_014196267.1      ACTGTGGATTACCTCCTCCGACTGCAGGAGTCAATCAGTGATTTCTACTGGTATTACTCT
XM_014129250.1      ACTGTGGATTACCTCCTCCGACTGCAGGAGTCAATCAGTGATTTCTACTGGTATTACTCT
                    ************************************************************

XM_014196267.1      GGGAAAGATATCATTGATGAGCCAGGCAAGAAGAACTTCTCCAAGGCCATGACAGTGGCT
XM_014129250.1      GGGAAAGAGATCATCGATGAGCCAGGCAAGAAGAACTTCTCCAAGGCCATGACAGTGGCT
                    ******** ***** *********************************************

XM_014196267.1      AAACAGATCTTCAACAGTCTGACTGAGTACATCCAGGGTCCGTGTACAGGCAACCAGCAG
XM_014129250.1      AAACAGATCTTTAACAGTCTGACTGAGTACATCCAGGGTCCGTGTACAGGCAACCAGCAG
                    *********** ************************************************

XM_014196267.1      TCCCTGACCCACAGCAGGCTGTGGGATGCAGTGGTGGGCTTCCTCCACGTCTTTGCCCAC
XM_014129250.1      TCCCTGACCCACAGCAGGCTGTGGGATGCAGTAGTAGGCTTCCTCCACGTCTTTGCCCAC
                    ******************************** ** ************************

XM_014196267.1      ATGATGATGAAGCTGGCACAGGGTAAAGATTCCAGCCAGATTGGCCTGCTGAAGGAGCTA
XM_014129250.1      TTAATGATGAAGCTGGCACAGGGTAAAGACTCCAGCCAGATTGGCCTGCTGAAGGAGCTA
                     * ************************** ******************************

XM_014196267.1      CTGGACCTCCAAAAAGACATGGTGGTTATGCTGCTCTCTCTACTGGAGGGTAATGTTGTG
XM_014129250.1      CTGGACCTCCAGAAAGACATGGTGGTTATGTTGCTCTCTCTACTAGAGGGTAATGTGGTG
                    *********** ****************** ************* *********** ***

XM_014196267.1      AATGGCACCATTGCCCGTCAGATGGTGGACATGCTGGTGGAATCGTCCAGCAACGTGGAG
XM_014129250.1      AACGGCACCATTGCCCGTCAGATGGTGGACATGCTGGTGGAATCCTCCAGCAACGTAGAG
                    ** ***************************************** *********** ***

XM_014196267.1      ATGATCCTCAAATTCTTTGACATGTTTCTCAAACTGAAGGACATTGTGGCGTCAGACGCC
XM_014129250.1      ATGATCCTCAAGTTCTTTGACATGTTCCTCAAACTGAAGGACATTGTGGCGTCAGACGCC
                    *********** ************** *********************************

XM_014196267.1      TTCCGTGATTACGTGACCGACCCCCGAGGGTTGATCTCTAAGAAGGACTTTCAGAAGGCC
XM_014129250.1      TTCCGTGATTACGTGACTGACCCCCGAGGGTTAATCTCTAAGAAAGACTTTCAGAAGGCC
                    ***************** ************** *********** ***************

XM_014196267.1      ATGGACAGCCAGAAACAGTACTCTCCCTCTGAGATCCAATTCCTGTTGTCCTGCTCAGAG
XM_014129250.1      ATGGACAGCCAGAAACAGTATTCTCCCTCCGAGATCCAATTCCTGTTGTCCTGCTCAGAG
                    ******************** ******** ******************************

XM_014196267.1      GCCGATGAGAATGACATGATCAACTTTGAGGAGTTTGCCGACCGCTTCCAGGAGCCAGCT
XM_014129250.1      GCTGATGAGAACGACATGATAAACTTTGAGGAGTTTGCCGACCGCTTCCAGGAGCCAGCT
                    ** ******** ******** ***************************************

XM_014196267.1      AAAGACATTGGATTTAACATTGCAGTGTTGCTGACCAACCTGTCTGAACATGTCCCTCAT
XM_014129250.1      AAAGACATTGGCTTTAACATTGCAGTGTTGCTAACCAACCTGTCTGAACATGTGCCTCAT
                    *********** ******************** ******************** ******
```

```
XM_014196267.1      GACCTCAGGCTGAAGAACTTCTTAGAGCAGGCAGAGAGTGTACTCAACTACTTCCGCCCC
XM_014129250.1      GACCTCAGGCTGAAGAACTTCCTAGAGCAGGCAGAGAGTGTACTCAACTACTTCCGCCCC
                    ********************* **************************************

XM_014196267.1      TTCCTGGGCCGTATAGAGATCATGGGTGCCAGCAGGAAGATAGAGCGTATTTACTTTGAG
XM_014129250.1      TTCCTGGGCCGTATAGAGATCATGGGTGCCAGCAGGAAGATAGAGCGTATTTACTTTGAG
                    ************************************************************

XM_014196267.1      ATCAGCGAGGTCAACCGCACACAGTGGGAGATGCCTCAGGTCAGAGAGTCCAAACGACAG
XM_014129250.1      ATCAGCGAGGTAAACCGCACACAGTGGGAGATGCCTCAGGTCAGAGAGTCCAAACGACAG
                    *********** ************************************************

XM_014196267.1      TTCATCTTTGATGTGGTCAATGAGGGGGGCGAGTCGGAGAAGATGGAAATGTTTGTCAAC
XM_014129250.1      TTCATCTTTGATGTGGTTAATGAGGGGGGCGAGTCGGAGAAGATGGAGATGTTTGTCAAC
                    ***************** ***************************** ************

XM_014196267.1      TTCTGCGAGGACACCATCTTTGAGATGAACATCGCCTCCCAGATCTCAGAGCAGGAGGAG
XM_014129250.1      TTCTGCGAGGACACTATCTTTGAGATGAACATCGCCTCCCAGATCTCGGAACAGGAGGAG
                    ************** ******************************** ** *********

XM_014196267.1      GAGAAGGAGGAGGAGGATGATGATGAGCCGGCTGAAGGAGGAGCTGAGGCAGGAGGAGGT
XM_014129250.1      GAGGAGAAGGAGGAGGATGATGATGATCCAGCTGAAGGAGGAACTGATGCAGGAGGAGG-
                    *** ** ******************* ** ************ **** *********** 

XM_014196267.1      GGAGGGGGAGATGAAGAGGGCAATGGAGAGGAAGGAGAGCCAGAGTCCAGCTCTGCCTTT
XM_014129250.1      --AGGTGGAGATGAAGAAGGCAATGGAGAGGAAGGAGAGCCAGAGTCCATCTCTGCCTTT
                      *** *********** ******************************* **********

XM_014196267.1      GCAGACTTCATCAACAGCTTATTGAACTTCCTGAGTATCTTCACCTTCCGTAACCTGCGC
XM_014129250.1      GCAGACTTCATCAACAGCTTATTGAACTTCCTGAGTATCTTCACATTCCGTAACCTGCGA
                    ******************************************** ************** 

XM_014196267.1      AGGCAGTACCGCAGAGTGAGGAAGATGACCATCAAGCAGATAGTGGTGGGTCTGGCCACC
XM_014129250.1      CGGCAGTACCGCAGGGTAAGGAAGATGACCATCAAGCAGATAGTGGTGGGTCTGGCTACC
                     ************* ** ************************************** ***

XM_014196267.1      TTCTTCTGGACTATCCTCATTGGCATCCTGCACTTCATTTACAGTGTATGCAAGGGCTTC
XM_014129250.1      TTCTTCTGGACTATCCTCATCGGCATCCTGCACTTCATCTACAGTGTATGTAAGGGCTTC
                    ******************** ***************** *********** *********

XM_014196267.1      TTCCTGCTCATTTGGCAAACTCTATTTGGAGGAGGCCTGGTGGAGGGAGCCAAGAACATC
XM_014129250.1      TTCCTGCTCATCTGGCAAACTCTATTTGGCGGAGGCCTGGTGGAGGGAGCAAAGAACATC
                    *********** ***************** ******************** *********

XM_014196267.1      ACAGTGACAGAGATCTTGGCCAGTATGCCCGACCCCACCCAGGACGAGGTGCACGGGGAC
XM_014129250.1      ACGGTGACAGAGATCCTGGCCAGTATGCCTGACCCCACCCAGGATGAGGTGCATGGGGAC
                    ** ************ ************* ************** ******** ******

XM_014196267.1      CTGCCAGGGGAGCCTAGGACTGGGGAGGAGCAGGAAGCAGGAGGGGTGACTGACCAAATG
XM_014129250.1      CTGCCAGAGGAGCCTGAGGTTGGGAAGGAGCAGGAGGCAGAAGGGGTGACTGACCAAATA
                    ******* *******  *  **** ********** **** ****************** 

XM_014196267.1      GACACAGGCGGTGGAGAGGAGGAGGAGGAGGA---CAACCAAGACAAGGAAGGTGGGGGG
XM_014129250.1      GACACAGGCGGTGGAGAGGAGGAGGAGGAGGAGGACAAAAAAGACAAGGAAGGTGGGGGG
                    ********************************   ***  ********************

XM_014196267.1      CCACCACGCATTGATGCTCCAGGAGGACTTGGTGACATGGGAATTGAGGCCACTGTTGAG
XM_014129250.1      ACCCCACGCATCGATGCTCCTGGTGGACTTGGTGACATGGGAATTGAGGCCACTGTTGAG
                     * ******** ******** ** ************************************

XM_014196267.1      CCTCCTACTCCTGAGGGTACTCCTTTGACCAGGAGGAAACAGCAACCAGAGGAGGGTGCT
XM_014129250.1      CCTCCTACTCCCGAGGGAACTCCTTTGACCAAGAGGAAACCGCAACCAGAGGAGGGTGCT
                    *********** ***** ************* ******** *******************

XM_014196267.1      GCTGCAGCTGCTGATGGCCAAGCTGCTGA-----------------ACCT-GCTCCTGCT
XM_014129250.1      GCTGCAGCAGCTGATGGCCAAGCTGTTGATGGCCAAGCAGCTGATGGCCTAGCTGCTGAA
                    ******** **************** ***                  *** *** ***  
```

```
XM_014196267.1      CCTGCTCCTGCCATAGAGGAGCCCCCTCCAGAGCCCGAGAAGGCAGACACTGAAAATGGA
XM_014129250.1      CTGGCTCCTGCCATAAAGGAGCCCCCTCCAGAGCCTGAGAAGGCAGACACTGAAAGTGGA
                    *  ************ ******************* ******************* ****

XM_014196267.1      GAGAAGGCTGAGAAGGAAGCCGAGACCAAAGAGGAGGAGAAACAGGAGCAACCGAAGGAA
XM_014129250.1      GAGAAGGCCGAGAAAGAAGCTGAGAACAAAGAGGAG---AAGCAGGAGGAAGCGATAGAA
                    ******** ***** ***** **** **********   ** ****** ** ***  ***

XM_014196267.1      AAGAAGGCCAAGGATAAGAAAAATAAGAAACAACACCGCGAGCAAGGGTTCCAACTCTGG
XM_014129250.1      AAGAAACCCAAGTCTAAGAAAGATAAGAAGCAACACAGGGAGCAAGGGTTCCAACTCTGG
                    *****  *****  ******* ******* ****** * *********************

XM_014196267.1      ACTGAGCTGGACATCCAAAGAAACAAATTCTTGAACTACCTCTCTCGGAACTTTTACAAT
XM_014129250.1      ACTGAGCTGGACATCCAAAGAAACAAATTCTTGAACTACCTCTCTCGGAACTTTTACAAT
                    ************************************************************

XM_014196267.1      CTGCGTTTTCTGGCACTGTTCATTGCATTTGCTCTAAACTTCATTCTACTCTTCTACAAG
XM_014129250.1      CTGCGTTTTCTGGCACTGTTCATTGCATTTGCCCTGAACTTTATTCTACTCTTCTACAAG
                    ******************************** ** ***** ******************

XM_014196267.1      GTGTCGGACAGCCCCCCTGGAGAAGGTGATGAGGTGGAGGGCTCTGGCATGTTTGAGGGC
XM_014129250.1      GTGTCGGACAGCCCCCCTGGAGAAGGTGATGAGGTGGAGGGCTCTGGCATGTTTGAGGGC
                    ************************************************************

XM_014196267.1      TCGGGGGTGGGGGCGCTGTTTGAGGGCAGTGGGCTGTTTGAGGGCTCAGCAGAAGAGATG
XM_014129250.1      TCAGGGTTGGGAGTGCTGTTTGAGGGAAGTGGGCTGTTTGAGGGCTCAGCGGAGGAGATG
                    ** *** **** * ************ *********************** ** ******

XM_014196267.1      GAGGGATCTGGTGGGGATGAAGGAGGAGATGAGGAGGAGGAGGAGGGCCCAGTCTACTTC
XM_014129250.1      GAGGGATCTGGTGGGGAGGAAGGAGGAGACGAGGAAGAGGAGGGGGGCCCAGTCTACTTC
                    ***************** *********** ***** ******* ****************

XM_014196267.1      TTCTTGGAGGAGAGCACTGGCTACATGCAGCCCACTCTCACATTCCTGGCCGCCCTGCAC
XM_014129250.1      TTCTTGGAGGAGAGCACTGGCTACATGCAGCCCACCCTCACCTTCCTGGCTACCCTGCAC
                    *********************************** ***** ********  ********

XM_014196267.1      ACAGTCATCGCCTTCCTCTGCATCATCGGCTACAACTGTCTAAAGATCCCTCTGGTGATC
XM_014129250.1      ACCGTCATCGCCTTCCTCTGCATCATCGGTTACAACTGTCTAAAGATCCCTCTTGTGATC
                    ** ************************** *********************** ******

XM_014196267.1      TTTAAGAGGGAGAAGGAGCTGGCCAGGAAGCTGGAGTTTGATGGTCTTTACATCACAGAG
XM_014129250.1      TTTAAAAGAGAAAAGGAGTTGGCCAGGAAACTGGAGTTTGATGGTCTTTACATCACAGAG
                    ***** ** ** ****** ********** ******************************

XM_014196267.1      CAGCCTGAGGATGATGACATCAAAGGCCAATGGGACAGACTGGTCCTCAACACACCTTCT
XM_014129250.1      CAGCCTGAGGATGATGACATCAAAGGCCAATGGGACAGACTGGTACTCAACACACTCTCT
                    ******************************************** **********  ***

XM_014196267.1      TTCCCAAACAACTACTGGGACAAGTTTGTTAAGCGAAAGGTCCTGGATAAGTATGGAGAC
XM_014129250.1      TTCCCAAGCAACTACTGGGACAAGTTTGTTAAGCGAAAGGTCCTGGATAAGTATGGAGAT
                    ******* ***************************************************

XM_014196267.1      ATTTATGGCAGAGAGAGGATCGCAGAGCTCCTAGGCATGGATTTAGCCTCGCTGGACGTC
XM_014129250.1      ATTTACGGCAGAGAGAGGATTGCAGAGCTCCTAGGCATGGATTTAGCCTCGTTGGATGTC
                    ***** ************** ****************************** **** ***

XM_014196267.1      AGTCAACAAACTGATAAGAAGCCGGAGGAACCAGACAATTCCACGTTGGCCTGGTGTACA
XM_014129250.1      AGTCAACAAACTGATAAGAAGCTGGAGGAACCCGACAATTCTACGTTGGCCTGGTTTACG
                    ********************** ********* ******** ************* ***

XM_014196267.1      TCTATTGACTTTAAGTACCAGATCTGGAAATGTGGAGTTGTGTTCACAGACGGGACTTTT
XM_014129250.1      TCTATTGACTTCAAGTACCAGATCTGGAAATTTGGAGTTGTGTTCACAGACGGGACTTTT
                    *********** ******************* ****************************

XM_014196267.1      CTCTATCTGTGTTGGTACACGATCATGTCCTTGCTTGGACATTACAACAACTTCTTCTAT
XM_014129250.1      CTCTATCTGTGTTGGTACACGATCATGTCCTTTCTTGGACATTACAACAACTTCTTCTAT
                    ******************************** ***************************
```

```
XM_014196267.1      GCCTGTCACTTGCTGGACATAGCCATCGGTGTGAAGGATCTGCGTACTATCCTGTCCTCT
XM_014129250.1      GCCTGTCACTTGCTGGACATAGCCATCGGTGTGAAGGATCTGCGTACTATCCTGTCCTCT
                    ************************************************************

XM_014196267.1      GTCACCCACAATGGGAAACAGCTCATGATGACATTGGGCTTGTTGGCAGTGGTGGTGTAT
XM_014129250.1      GTCACCCACAATGGGAAACAACTCATGATGACATTGGGCTTGTTGGCAGTGGTGGTGTAT
                    ******************** ***************************************

XM_014196267.1      CTCTACACTGTAGTGGCCTTTATCTTCTTCCGCAAGTTTTACAACAAGAGTGAAGATGAG
XM_014129250.1      CTCTACACTGTAGTGGCCTTTATCTTTTTCCGCAAGTTTTACAACAAGAGTGAAGATGAG
                    ************************** *********************************

XM_014196267.1      GATGAGCCGGATATGAAATGTGATGACATGATGACTTGCTACCTCTTCCACATGTACGTG
XM_014129250.1      GATGAGCCGGATATGAAATGTGACGACATGATGACTTGCTACCTCTTCCATATGTACGTG
                    *********************** ************************** *********

XM_014196267.1      GGAGTGCGTGCTGGCGGTGGCATAGGGGACGAGATCGAGGACCCGGCGGGAGACGTGTAC
XM_014129250.1      GGCGTGCGTGCTGGCGGTGGCATAGGGGACGAGATCGAGGACCCGGCAGGAGACGTGTAC
                    ** ******************************************** ************

XM_014196267.1      GAGCTCTACCGGGTGGTCTTTGACATCACCTTCTTCTTCTTTGTCATTGTCATCCTGTTG
XM_014129250.1      GAGCTCTACCGGGTCGTCTTTGACATCACCTTCTTCTTCTTTGTCATTGTCATCCTGTTG
                    ************** *********************************************

XM_014196267.1      GCCATCATCCAGGGTCTGATCATTGACGCCTTCGGAGAGCTCCGAGACCAACAAGAGCAG
XM_014129250.1      GCCATCATCCAGGGTCTGATCATTGATGCCTTCGGAGAGCTCAGAGACCAACAGGAGCAG
                    ************************** *************** ********** ******

XM_014196267.1      GTTAAGGAAGACATGGAGACCAAGTGCTTCATCTGTGGAATTGGAAGCGACTACTTTGAT
XM_014129250.1      GTTAAGGAAGACATGGAGACCAAGTGCTTCATCTGTGGAATTGGAAGCGACTACTTTGAT
                    ************************************************************

XM_014196267.1      ACGACGCCGCACGGCTTCGAGACCCACACCCTGGAGGAACACAACTTGGCCAACTACATG
XM_014129250.1      ACGACGCCGCACGGCTTCGAGACCCACACCCTGGAGGAACACAACTTGGCCAACTACATG
                    ************************************************************

XM_014196267.1      TTCTTCTTAATGTACCTCATCAACAAAGATGAGACGGAGCATACTGGCCAGGAGTCATAT
XM_014129250.1      TTCTTCTTAATGTACCTCATCAACAAAGATGAGACGGAGCACACTGGCCAGGAGTCGTAT
                    ***************************************** ************** ***

XM_014196267.1      GTGTGGAAGATGTACCAGGAGCGAGCGTGGGACTTCTTCCCTGCTGGGGACTGTTTCAGG
XM_014129250.1      GTGTGGAAGATGTACCAGGAGCGAGCGTGGGACTTCTTCCCTGCTGGCGACTGTTTCAGA
                    *********************************************** ***********

XM_014196267.1      AAGCAATATGAGGATCAGCTTGCATAACCATACT----------ACTACAATTTGTAGGT
XM_014129250.1      AAGCAATATGAGGATCAGCTTGCATAACCATACTGTAACGTACTACTACAATTAGTAAGT
                    **********************************          ********* *** **

XM_014196267.1      TTAATAACATATTAAACCGTACTGAAATAGCTGTACTATACTGTATGGAAGAGAGTTGGG
XM_014129250.1      TTAATAACATACTGTAACGTACTACTCCAATT--AGTAGGTTTAATAACATACTGTAACG
                    *********** *  * ******     *  *  * **   *  **   * *  **   *

XM_014196267.1      CCCAAACACAT---GCAAACTAAATAACTGCGTTTAGAAGTGTTTGTAGCAATGTCAAGT
XM_014129250.1      TACTACTACAATTAGTAGGTTTAATAAC-ATACTGTAACGTACTACTACAATTAGTAGGT
                      * *  ***    * *   * ******     *   * **  *  **  * *   * **

XM_014196267.1      TGAA--ACATACAGTGACAGTAATTCTTGAAATGTCA--TTGTGCGAGTGAATGTGGGAA
XM_014129250.1      TTAATTACATACTGTAAC-GTACTACTACAATTAGTAGATTTAATAACATACTGTAACGT
                    * **  ****** ** ** *** * **  ** *   *  **     *   * ***

XM_014196267.1      AATACTCCCGTTTGCCAACACACACAAATATTCTCA---ATAATTAAGTGCATTATGCAT
XM_014129250.1      ACTACTACAATTAGTAGATTTAATAACATACTGTAACGTACTACTACAATTAGTAGATTT
                    * **** *  ** *   *   *   * *** * * *   *  * **     * **    *

XM_014196267.1      GTCAATTTTGTAAAACTTCAGTGCCTAAATTATTGGAACAAACTTTAT-TAGTCGCATGC
XM_014129250.1      AATAACATACTGTAACGT-ACTACTACAATTAGTAGATTTAATAACATACTGTAACGTAC
                       **  *  *  *** * * * *   ***** * **   **    **   **  * * *
```

```
XM_014196267.1      TACAGCAGC------ATCTTGATACTGTACCAAAAC-TGTCATTATTGGTATAGTACTTT
XM_014129250.1      TACTACAATTAGTAGATTTAATTACCATACTGTAACGTACTACTACAATTAGTAGATTTA
                    ***  **        ** *   ***  ***   *** *   * **    **    * **

XM_014196267.1      GATACCCGGCTATCTCCTCCTACT-CATAAAGCAAGA--GATACCGGTACCA-GATATAC
XM_014129250.1      ATAACCATACTGTAACGTACTACTACAATTAGTAGGTTTAATAACCATACTGTAACGTAC
                       ***   ** *  * * ***** **   ** * *    *** *  ***    *  ***

XM_014196267.1      TGTAAACACAACTGGAGT--ATTGTTGATGGTACTTTATCCTTTAAAGCTATTTACACAA
XM_014129250.1      TACTACAATTAGTAAGGTTAATAACATACTGTAACGTACTACTCCAATTAGTAGGTTTAA
                    *   *  *  * *   **  **     *  ***   **    *  **    *      **

XM_014196267.1      CAGTCTCGTAGATAATATTTTACCAGCC------CACTGGAAAAGCTCTAGAACC-ACTA
XM_014129250.1      TAACATACTAAACCAGACTGAAATAGCTGTACTATACTGTAACCATACTAAACCAGACTG
                     *   *  ** *  * * *  *  ***        **** **     *** * *  ***

XM_014196267.1      CA-------TACAGTACAGTATTGACTTTAAAT-GCTCTTCAACTGCTGCTTTTCAAT-T
XM_014129250.1      AAATAGCTGTACAATACTGTAACCATACTAAACCAGACTGAAATAGCTGTACTATACTGT
                     *       **** *** ***   *   ****     **  **  ****   *  * * *

XM_014196267.1      GACAAGGTTTTTCC----TGAAAT----GTTCT---TTGTATCTGTTTTAATTATGACT-
XM_014129250.1      AACCATACTAAACCAGACTGAAATAGCTGTACTATACTGTAACCATACTAAACCAGACTG
                     ** *   *   **    ******    ** **    **** *  *  ***    ****

XM_014196267.1      ATTTCTCTCAACAGAACTG--ACGATGGTTA-CTGTGTTGTTGACGTAGTA-ACTAGGGT
XM_014129250.1      AAATAGCTGTACTATACTGTAACCATACTAAACCAGACTGAAATAGCTGTACAATACCGT
                    *  *  **  **   ****  ** **  * * *     **     *  *** * **  **

XM_014196267.1      TTGTGTATTTATTCGTTTTTAATTTATTGAA-TGTATTTTAAGCCTGGCTAGTCTGAACG
XM_014129250.1      AACCATACTAAACCAGACTGAAATAGCTGTACTATACCGTAACCATACTAAACCAGACTG
                         ** * *  *    * ** *   ** * * **   *** * *    *  * **  *

XM_014196267.1      CCATGAATGCATTAT--TGTATCCAGTACCTTGTTTAGTTTGAGGGAACT-CACTATTTT
XM_014129250.1      AAATAGCTGTACTATACTGTAACCA-TAC-TAAACCAGACTGAAATAGCTGTACTATACT
                      **   ** * ***  **** *** *** *     **  ***   * **  *****  *

XM_014196267.1      GTTTTCTTA--AAACCAAAAAAAATACTGGTTTATTAATGGGTTTGTTCATTGGTTTATT
XM_014129250.1      GTAACCATACTAAACCAGACTGAA-ATAGCTGTACTACACTGTAACCATACTAAACCAGA
                    **   * **  ****** *   ** *  * * ** **    **      * *     *

XM_014196267.1      AAATAAAGGCTTTGATGCAC----------------------------------------
XM_014129250.1      CTGAAATAGCTGTACTATACTGTAACCATACTAAACCAGACTGAAATAGCTGTACTATAC
                        **  *** *  *  **

XM_014196267.1      ------------------------------------------------------------
XM_014129250.1      TGTAACCATACTAAACCAGACTGAAATAGCTGTACTATACTGTAACCATACTAAACCAGA

XM_014196267.1      ------------------------------------------------------------
XM_014129250.1      CTGAAATAGCTGTACTATACTGTAACCATACTAAACCAGACTGAAATAGCTGTACTGTAC

XM_014196267.1      ------------------------------------------------------------
XM_014129250.1      TGTAACCATACTAAACCAGACTGAAATAGCTGTACTATGCTGTAACCATACTAAACCAGA

XM_014196267.1      ------------------------------------------------------------
XM_014129250.1      TTGAAATAGCTGTACTATACTGTACGGACCAGGGTGAAGGCACATTTATAGTGCTAGACC

XM_014196267.1      ------------------------------------------------------------
XM_014129250.1      AAAAGAGGGCTTTTGTCCCTCTCGTTCGAATAGACCAAACACATGCAAACTAAATGACTG

XM_014196267.1      ------------------------------------------------------------
XM_014129250.1      CATTTAGAATATGTGTTTGTTACCATGTCAATTCTTGAAATGTGTGAATGAATGTGGGAA
```

```
XM_014196267.1      ------------------------------------------------------------
XM_014129250.1      ATACTCCCTATTGCCAGCACACACAAATATGCACAATAATGAAGTGCATATGTAGCAATA

XM_014196267.1      ------------------------------------------------------------
XM_014129250.1      ACAATTACATATTTCAATCAATGTCATCTCTCACACACAGTGCACATTGTTTGTCTGTGT

XM_014196267.1      ------------------------------------------------------------
XM_014129250.1      TTGTAGACAATACGAATGTACATTTCTGTCAAATTTCAGTGCCTAAATCGTTTGAACAAA

XM_014196267.1      ------------------------------------------------------------
XM_014129250.1      CTTTATTAGTTGCATTGTTGTACTACTTTGTACTATAAGCCCAACTAGAGTATAGTGGCT

XM_014196267.1      ------------------------------------------------------------
XM_014129250.1      GGTACTTTTATAAAAAATGTAAAACAGAAATACCTCATTGACATAAGTATTAAGTAACTA

XM_014196267.1      ------------------------------------------------------------
XM_014129250.1      TTTAAGTAACAAGGACTAAATACAAGTACCACATTTGTGACAGTTGCTTATTTTCTCTCT

XM_014196267.1      ------------------------------------------------------------
XM_014129250.1      CTCTCTCTCTCTCTGAGTGATCGTTTGGAGTGACTACGTGGGAGATAGTCACAGATTACA

XM_014196267.1      ------------------------------------------------------------
XM_014129250.1      TTGATTTTTCCTTTAAAACAATGCACTCTGAGACTAATGACACAACAATCTCTTTGATAA

XM_014196267.1      ------------------------------------------------------------
XM_014129250.1      TATTTGACTATTCCGCTTGAATTTCCATCCCACTGGAACAGCTCTATAACCACTCATACA

XM_014196267.1      ------------------------------------------------------------
XM_014129250.1      GTACATTGAATGTGTTGACTGCCGTACTTCTTTAAATGCTCTTCAACTGCTACTTTTCAA

XM_014196267.1      ------------------------------------------------------------
XM_014129250.1      CTGACAACATTTGTGTTGCATACACTACAGCTTGTCCTGAAACCTTAATAATAATGTTGA

XM_014196267.1      ------------------------------------------------------------
XM_014129250.1      TTATTCTTATTTTTCTCAATAGAACTAATTATGGTTACTGTGCTGTTAACGTAGTAACTA

XM_014196267.1      ------------------------------------------------------------
XM_014129250.1      GGGTTTGTGTATTTTTTCGTTTTTAGTTTATTCAATGTTTTTTGAATGCTGACTCTAGTC

XM_014196267.1      ------------------------------------------------------------
XM_014129250.1      TGTGAACACTATGAATGCATTATTGTATCCAATACATTGTTTAGTTTAAGAGAACTCACA

XM_014196267.1      ------------------------------------------------------------
XM_014129250.1      ACTTTGTGTTCTTAAAACAAAGAATGGTTTATTAATGAGTTTGTTCATTGGTTTATTAAA

XM_014196267.1      ----------------------
XM_014129250.1      TAAAGGTATTGATGCACTTGCA
```

CLUSTAL 2.1 Multiple Sequence Alignments for *odc1*

Sequence 1: XM_014211026.1(*odc1a*) 2574 bp
Sequence 2: XM_014192087.1 (*odc1b*)2605 bp

Sequences (1:2) Aligned. Score: 75.6022

CLUSTAL 2.1 multiple sequence alignment

```
XM_014211026.1      TTCTAGAGCACATGGAGCCGGTGACAAGACTGAACCGGTTCCTACGTCTACAGAAGGCCG
XM_014192087.1      ----------------------------------TTCCATCCGACGTCGATGGAGGGCCA
                                                       *** ***** *  ** ****

XM_014211026.1      GTTTCAGCTGGCGTGTTTCCTTGGAGACGGCACCTCTATAAATACGGTC-GTATCGAAAC
XM_014192087.1      GTTCCAGCTGGCGTGTTGTCTTGGAGACGGAAACTCTATAAATACAGTCAGTATCGAAAC
                    *** *************  *********** * ************ *** **********

XM_014211026.1      GTTGGCTTCAACTTTCCAGTTGTAATTCAGTTAGAGAGCGCGACACAGTTTCGTGACAGA
XM_014192087.1      ACAGACTTACACTT----------GTTCAGACCGAGAGAGTTATTCAGTTCTTTGACAGA
                       * ***  ****           *****   ***** *  *  *****   *******

XM_014211026.1      CTCGGCTGACGAGGAAAAACCAAGTCATATTTTCCAGGATTCTGGTCTAATGGACACACT
XM_014192087.1      TTCGTCTGACAAGGGAAAACCAAGTAAT-TTTTCCAGGATTCGGGTCTAATGGACACACT
                     *** ***** *** ********** ** ************* *****************

XM_014211026.1      CTTTGTAAATGCTTTTTAAGAATAATCGCTTTAGGAAAGCGCACACTGGCTACTCTGCAG
XM_014192087.1      CTTTGTAAATGCTTTTTAAGAATAATCGCTTTAGGAAAGTGCAGACTGGCAACTCTGCAG
                    *************************************** *** ****** *********

XM_014211026.1      GCCCCGGCCTAGAGTCTTATCTGAAAGGCTTCAACTTCACTGGTGAACCACCGTGGCTGC
XM_014192087.1      GCCCCGGCCTAGAGTGTTATGTGAAAGGTTTCAACTTCACTGGTGAACCGCCTTGGCTGC
                    *************** **** ******* ******************** ** *******

XM_014211026.1      TCTGCACCTGATTAAACTACTTTGCCAAATTCCATCTCTTTCTTGTCCAATT--CTTTTG
XM_014192087.1      TCTGCACCTGATTAAACTACTTTGCCAAATTCCAACTCTTTCTTGTCCAGTTTTCTTTTG
                    ********************************** ************** **  ******

XM_014211026.1      TGACAATCTTTAAATATTTTCTTCGACCATGAACACTTTTGCTCCTG---ATTTTGTCTT
XM_014192087.1      TGACCAATCTAAAAGTTATTCTGCGACCATGAACACTTTCGCTCCTGCCGATTTTTCCTT
                    **** *   * ***  * **** **************** *******   *****  ***

XM_014211026.1      CCTGGAGGAGGGGTTCTGTGCCCGTGACATTGTTGAGCAAAAGATCAACGAGACATCCAT
XM_014192087.1      CCTGGAGGAGGGATTCTGTGCCCGTGACATTGTTGAGCAGAAGATCAATGAGATGTCCAT
                    ************ ************************** ******** ****  *****

XM_014211026.1      GTCTGATGATAAGGATGCCTTCTACGTGTGCGACTTGGGTGACGTGCTGAAGAAGCACAT
XM_014192087.1      GTCGGATGATAAGGATGCCTTCTACGTGTGCGACTTGAGTGACGTGCTGAAGAAGCACAT
                    *** ********************************* **********************

XM_014211026.1      GCGATGGGCGCGTGCCATGCCTCGCGTCACGCCCTTCTATGCTGTCAAATGCAACGACAG
XM_014192087.1      GCGATGGGCGCGTGCCATGCCTCGTGTAACGCCCTTCTATGCTGTCAAATGCAACGACAG
                    ************************ ** ********************************

XM_014211026.1      CCGGGCTGTTGTCACGACCCTGGCCTCCTTGAACGCTGGCTTTGACTGTGCAAGCAAGAC
XM_014192087.1      CCGGACTGTTGTCACGACCTTGGCCTCCTTGGGCGCTGGCTTTGACTGTGCAAGCAAGAC
                    **** ************** ***********  ***************************

XM_014211026.1      TGAGATCCAGATCGTTCAGTCTCTGGGTGTGGACGCTAACAGGATCATCTACGCCAACCC
XM_014192087.1      TGAGATCCAGATTGTTCAGTCTCTGGGTGTGGATGCTAGCAGGATCATCTACGCCAACCC
                    ************ ******************** **** *********************

XM_014211026.1      CTGCAAGCAGGTGTCCCAGATCAAGTATGCCTCTGCCCACGGAGTGCAGATGATGACCTT
XM_014192087.1      TTGCAAGCAGGTATCCCAGATCAAGTATGCCTCTGCCCACGGAGTGCAGATGATGACCTT
                     *********** ***********************************************
```

```
XM_014211026.1      CGACAGCGATGTGGAGCTCATGAAGGTGGCTCGGTACCACGACGATGCCAAACTGGTACT
XM_014192087.1      CGACAGCGACGTGGAGCTCATGAAGGTGGCTCGGTGCCACGACAATGCCAAACTAGTCCT
                    ********* ************************* ******* ********** ** **

XM_014211026.1      GCGCATTGCCACAGATGATTCCAAGGCAGTGTGCCGGCTGAGTGTGAAGTTTGGTGCCAC
XM_014192087.1      GCGCATTGCCACAGATGATTCCAAGGCTGTGTGCCAGCTCAGTGTTAAGTTTGGTGCCAC
                    *************************** ******* *** ***** **************

XM_014211026.1      CCTGAAGGCCTGCCGGGGCCTCCTGGAGCGGGCTAAGGAGCTGGGCCTGGATGTTATCGG
XM_014192087.1      CATGAAGGCATGCCGAGGTCTCCTGGAGAGGGCTAAGGAACTGGGCCTGAATGTTATCGG
                    * ******* ***** ** ********* ********** ********* **********

XM_014211026.1      GGTCAGCTTCCACGTGGGCAGCGGCTGCACTGACCCAGACACCTACAGCCAGGCCATCTC
XM_014192087.1      GGTCAGCTTCCACGTGGGGAGCGGCTGCACTGACCCAGACACCTACAGCCAGGCCATCTC
                    ****************** *****************************************

XM_014211026.1      TGACGCACGCTGTGTCTTCGACATGGGGGCTGAGGTGGGATTCAACATGACCCTCCTGGA
XM_014192087.1      CGACGCTCGCTGTGTCTTTGACATGGGGGCCGAAGTAGGTTTCAACATGACCCTCCTGGA
                     ***** *********** *********** ** ** ** ********************

XM_014211026.1      CATTGGTGGTGGTTTCCCTGGGTCTGAGGACACCAAGCTCAAGTTTGAGGAGATCACAGC
XM_014192087.1      CATTGGTGGTGGTTTCCCTGGGTCTGAGGATACCAAGCTCAAGTTTGAGGAGATCACAGC
                    ****************************** *****************************

XM_014211026.1      AGTTATCAACCCAGCGCTGGACAAGTATTTTCCTGCCGACTCTGGGATCCGGATCATAGC
XM_014192087.1      AGTTATCAACCCAGCACTGGACAAGTATTTCCCTGCCGACTCTGGGATCCGGATCATTGC
                    *************** ************** ************************** **

XM_014211026.1      CGAACCAGGCCGCTACTACGTGGCCTCTGCCTACACCCTAGCTGTTAACATCATTGCCAA
XM_014192087.1      TGAGCCAGGCCGCTACTACGTGGCCTCTGCCTACACCCTAGCTGTTAACGTCATCGCAAA
                     ** ********************************************* **** ** **

XM_014211026.1      GAAGGTTATCATGAACGAGCAGTCTGCCTCTGACGAGGATGACGATTGGACCAGTGACCG
XM_014192087.1      GAAGGTCATCATGAACGAGCAGTCTGCCTCTGACGAGGATGACGATTGGACCAGTGACCG
                    ****** *****************************************************

XM_014211026.1      GACTCTGATGTACTATGTGAACGATGGGGTCTACGGCTCCTTCAACTGTATTCTATATGA
XM_014192087.1      GACTCTGATGTACTATGTGAACGATGGCGTCTATGGCTCCTTCAACTGTATTCTATATGA
                    *************************** ***** **************************

XM_014211026.1      CCATGCTCACCCCCTGCCTACCCTGCACAAGAAGCCAAAGCCAGATGAGCGTATGTACCC
XM_014192087.1      CCATGCTCACCCCTTGCCTACCCTGCACAAGAAGCCAAAGCCAGATGAGCGTATGTACCC
                    ************* **********************************************

XM_014211026.1      CTGCAGCATCTGGGGACCAACCTGCGACGGCCTGGACCGCATCGCTGAGGTGTGCACTCT
XM_014192087.1      CTGCAGCATCTGGGGACCAACCTGTGACGGCCTGGATCGCATCGCTGAGGTGTGCACTCT
                    ************************ *********** ***********************

XM_014211026.1      GCCGGACATGCAGGTGGGAGAGTGGCTGCTGTTTGAGAACATGGGGGCCTACACCGTGGC
XM_014192087.1      GCCGGACATGCAAGTGGGAGAGTGGCTGCTGTTTGAGAATATGGGGGCCTACACCGTGGC
                    ************ ************************** ********************

XM_014211026.1      TGCCTCCTCTACCTTCAACGGCTTCCAGAAGCCAGACATCTATTACACTATGTCCCGCAC
XM_014192087.1      TGCGTCCTCTACCTTCAACGGCTTCCAGAAGCCAGACATCTACTACATCATGTCCCGCAC
                    *** ************************************** ****  ***********

XM_014211026.1      AGCTTGGCAGTGCATGCAGCAGATCTGTGCCCAGGGGATGCCCGTTCCTTTGGAGGAGTC
XM_014192087.1      AGCTTGGCAGTGCATACAGCAGATCTGTGCCCAGGGGATGCCCACTCCTGTTGAGGATGC
                    *************** ***************************  **** * *****  *

XM_014211026.1      GTCCTGTGTGACATCCAGCTGTGGCCATGAGAGCAGCCTGGAGCTGCCCACCAAGCCTTG
XM_014192087.1      ATCCTGTGTGACAGCCAGCTGTGGCCATGAGAGCAGCCTGGAGCTGCCCGCCAAGTCTAG
                     ************ *********************************** ***** ** *

XM_014211026.1      CCAGACCCGTGTGCTCTAAACACAACACCAGCATAATCTCATACATATCCACCACACACT
XM_014192087.1      CCAGACCTGTGTGCTCTAAACACAACACCAACATAATCTCATACATACCCAC-------T
                    ******* ********************** **************** ****       *
```

```
XM_014211026.1      GCCACTCTACGGGCCCAGTAGCCTGTGTT---ATTAATTTCCTATTCAGT--------TT
XM_014192087.1      GCCATTCTACCGGCCCAGTAGCTTGTGTTTTTATTAATTTCCTATTTAATGTATTTTATT
                    **** ***** *********** ******   ************** * *        **

XM_014211026.1      TTTTTTTTAACTCATACCGTTGAAAGGCCAGTTACTTGACTGGAGAATGAG--GGGCATG
XM_014192087.1      TTATTTTTTACTCATACCGTTGAAAGGCCAGTTACTTGACGGGAGAATGAGAGGGGCATG
                    ** ***** ******************************* **********  *******

XM_014211026.1      TTGGTGCACATTTCTTACTTTTCTGTATGAAAGAC--AGATCTAATCCTGAGCTGAGGCT
XM_014192087.1      TTAGCGCACATTTCTCATTTATCTGTATGAAAGACTGAGATCTAATCCTGAGCTGAGGCT
                    ** * ********** * ** **************  ***********************

XM_014211026.1      GAATCTCTCA----------------ATGTCTAGGAGGGTTGTAGTCCTATTGTTACGGG
XM_014192087.1      GAATCTCTCTTAACATCACTAAAGGTACATCTCGAAGGGTTGGAGTCATATTGTTACATG
                    *********                 *  *** * ******* **** *********  *

XM_014211026.1      GGTCTGATTGGTTAATGCCTATAACACCAAGGTTGAATGTTCATTTATTTTAGGGGCCAC
XM_014192087.1      GTTCTGATTGGCTAATGCCTACA-----AAGGTTGTATGTTCAATTCCAGTAGGGGCCAC
                    * ********* ********* *     ******* ******* **    **********

XM_014211026.1      AGGATTCCTTCATTTGCACTGAGC-TAAGTCGCTCTGAATGAGAGCACTTGTATGTAAAA
XM_014192087.1      AAAACACCTTAATTTGCATTGACCATAAATCACTTTAGGTGAGAGCATTT----ACAAAA
                    *  *  **** ******* *** * *** ** ** *   ******** **      ****

XM_014211026.1      TGACAATGTAAATGTTACATGGCGGTGTTGTAAGTGGTTTTTCT------CTCCAACTCC
XM_014192087.1      TGACAATGTAAATGTTACATGGCAGTGTTGTAGGGATTTTCTCTTGTAAACTCCAATTCC
                    *********************** ******** *   *** ***      ****** ***

XM_014211026.1      TAGAAGGCTCC--ATTTCAGTTGACTCCACTACCACCTCTCATGACTCTAAAAGGGCTGT
XM_014192087.1      CAGAAGGCTCCCTGTTTCAATTGAATCCACAACCCAATCTCATGACTCTAAAGGGGCTGT
                     **********   ***** **** ***** ***   *************** *******

XM_014211026.1      TTGAGTGCTGATTACTTTGCCGGGTAATGGCTGCTACAGATATTTAATTTTCTCTGAA-T
XM_014192087.1      TTGAGGGCGGATTACTTCTCCAGGAAATGGCTGCTACAGATATTTAATTTTCCCTGATCT
                    ***** ** ********  ** ** *************************** ****  *

XM_014211026.1      CCTCT--------------------CAGTGTAGGGACTCAACATGCAGACTGTCCCTCTT
XM_014192087.1      CCTCTTTCATACAGAACCCTTTGTTCAATGTAGGGATTCAACATGCAGACTGTCCCTCTT
                    *****                    ** ******** ***********************

XM_014211026.1      CTCCTTTTCTGTATTTAATGTT-TTTTTTTTTGTTGCTGGGTAGTACATCAGTTAAT---
XM_014192087.1      CTCCTTTTCTGTATTTTTTGTAATTTTCTTTTGTTGCTAGGTAGTATGTCAGTTAATGAT
                    ****************  ***  **** ********** *******  *********

XM_014211026.1      ---------AATTATTCATGTTTAAGATGTAT------------ACGAAAGAGATGGGAT
XM_014192087.1      ACAAATAATAATTGTTACTATTGACGATGCATTTGAAAAAGGATACGAAGGAGATGGGAT
                             **** **  * ** * **** **            ***** **********

XM_014211026.1      TGCACATTTGTATTGCTTTCCTATGGAAACTTTTTATTTTGTATTTTAAATGTAATTCTA
XM_014192087.1      TGCACATTTGTATCACTTTCCTATGGAAACTTTTT-TTTAATTTTGTATTTTTTTTTTAA
                    *************  ******************** ***  * ** **  * *  **  *

XM_014211026.1      TATAAATAAAATGGGTGCAGAGCTGA-
XM_014192087.1      AATAAAATGTGTAGGTGCAGAGCTGAA
                     *****     * *************
```
