# Supplementary material for: Plant-Based Diets Induce Transcriptomic Changes in Muscle of Zebrafish and Atlantic Salmon
Source: Front Genet. 2020 Oct 22;11:575237. doi: 10.3389/fgene.2020.575237 (PMC7642599; doi:10.3389/fgene.2020.575237)
Supplement: Supplementary file 3 [file Data_Sheet_3.PDF]

Supplementary Tables 1-4

Plant-based diets induce transcriptomic changes in fast muscle of zebrafish and Atlantic salmon

Anusha K.S. Dhanasiri [1,2]*, Amritha Johny [3], Xi Xue [4], Gerd M Berge [5], Andre S. Bogevik[6], Matthew L. Rise [4], Christiane K. Fæste [3] and Jorge M.O. Fernandes [1]*

[1] Faculty of Biosciences and Aquaculture, Nord University, Bodø, Norway

[2] Department of Paraclinical Sciences, Faculty of Veterinary Medicine, Norwegian University of Life Sciences (NMBU), Oslo, Norway

[3] Toxinology Research Group, Norwegian Veterinary Institute, Oslo, Norway

[4] Department of Ocean Sciences, Memorial University of Newfoundland, St. John's, NL A1C 5S7, Canada

[5] Norwegian Institute of Food, Fisheries and Aquaculture Research (Nofima), Sunndalsøra, Norway

[6] Norwegian Institute of Food, Fisheries and Aquaculture Research (Nofima), Fyllingsdalen, Norway

Corresponding authors

Anusha K.S. Dhanasiri

Email: anusha.dhanasiri@nmbu.no

Jorge M.O. Fernandes

Email: jorge.m.fernandes@nord.no

Supplementary Table 1. Composition of the zebrafish and Atlantic salmon diets.

Diet composition (g/ 100g)	Control	PPC	SPC	WG
**Zebrafish**				
Fish meal [1]	79.35	49.35	49.35	49.35
Wheat [4]	12.0	12.0	12.0	12.0
PPC [3]	-	**30.0**	-	-
SPC [2]	-	-	**30.0**	-
WG [4]	-	-	-	**30.0**
Fish oil [1]	4.0	4.0	4.0	4.0
Additives *	4.65	4.65	4.65	4.65
Total crude protein	56.2	49.9	55.0	59.1
Total lipids	12.0	10.7	9.4	9.8
**Atlantic Salmon**				
Fishmeal [1]	63.35	-	33.35	33.35
Wheat [4]	12.0	-	12.0	12.0
SPC [2]	-	-	**30.0**	-
WG [4]	-	-	-	**30.0**
Fish oil [1]	20.0	-	20.0	20.0
Additives *	4.65	-	4.65	4.65
Total protein	45.2		44.0	48.1
Total lipids	26.5		23.8	24.3

PPC- pea protein concentrate, SPC- soy protein concentrate and WG- wheat gluten

* Vitamin mix (2%), Mineral mix (0.59%), Monosodium phosphate-24% P (2%), Yttrium oxide (0.01%), Carophyll Pink-10% (0.05%)

Ingredient suppliers:
[1] Norsildmel AS (Bergen, Norway)
[2] Agilia A/S (Videbæk, Denmark)
[3] AM Nutrition AS (Stavanger, Norway)
[4] Tereos Syral (Marckolsheim, France).

Supplementary Table 2. Genomic location and pairwise alignment details of paralogues used in this study.

**Paralogues**	**Genomic location**		**Pairwise alignment details***			
	Chromosome	Location	Pairwise alignment	Length (bp) of aligned region	% identity over aligned region	Associated E-value of aligned region
mylpfba	ssa06	24880606-24885367	*mylpfba : mylpfbb*	805	97	0.0
mylpfbb	ssa03	75692468-75696877	*mylpfba* : *mylpfbc*	468	71	1e-50
mylpfbc	ssa20	37559259-37568216	*mylpfbb* : *mylpfbc*	468	70	3e-48
hsp90aa1.1a	ssa06	72144452..72149825	*hsp90aa1.1a* : *hsp90aa1.1b*	2744	92	0.0
hsp90aa1.1b	ssa15	46699126..46705169				
ambra1aa	ssa26	9393199..9499944	*ambra1aa* : *ambra1ab*	4750	93	0.0
ambra1ab	ssa11	9352168..9464323	*ambra1aa* : *ambra1ac*	1850	76	0.0
ambra1ac	ssa16	1802087..1853353	*ambra1aa* : *ambra1ad*	1847	77	0.0
ambra1ad	ssa10	63803157..63851652	*ambra1ab* : *ambra1ac*	1851	75	0.0
			ambra1ab : *ambra1ad*	1842	75	0.0
			ambra1ac : *ambra1ad*	4728	88	0.0
col2a1aa	ssa12	84293497-84386605	*col2a1aa* : *col2a1ab*	2082	93	0.0
col2a1ab	ssa22	53429386-53512213	*col2a1aa* : *col2a1ac*	1533	84	0.0
col2a1ac	ssa13	21505732-21531640	*col2a1aa* : *col2a1ad*	1980	82	0.0
col2a1ad	ssa15	62188998-62218204	*col2a1ab* : *col2a1ac*	4169	83	0.0
			col2a1ab : *col2a1ad*	4706	82	0.0
			col2a1ac : *col2a1ad*	4473	93	0.0
btca	ssa01	117982644-17989859	*btca* : *btcb*	794	85	0.0
btcb	ssa13	89439773-89448007				
ryr1aa	ssa04	35701429-35771978	*ryr1aa* : *ryr1ab*	10715	95	0.0
ryr1ab	ssa11	77701604-77778088				

odc1a	ssa09	27129401-27134433	*odc1a* : *odc1b*	2636	86	0.0
odc1b	ssa01	29219775-29225435				

*Pairwise alignment details were manually obtained on 14.06.2020 from BLAST alignment of respective sequences in NCBI's nt database

Supplementary Table 3. List of selected differentially expressed genes in zebrafish muscle fed with the soy diet compared to their counterparts fed with fishmeal.

Gene symbol	Gene name	Selected GO terms*	Fold change	Base Mean	q-value
**Upregulated**					
klhl30	*kelch-like family member 30*	Protein ubiquitination	5.9	108.5	9.8E-16
dpp6b	*dipeptidyl-peptidase 6b*	Proteolysis	3.3	66.8	0.0014
gnsb	*Glucosamine(N-acetyl)-6-sulfatase*	Glycosaminoglycan metabolic process	3.1	8.7	0.0105
lmo7b	*LIM domain 7b*	Regulation of signaling	2.8	576.6	0.0013
loxl2b	*lysyl oxidase-like 2*	Oxidation-reduction process	2.7	826.9	0.0451
evpla	*envoplakin a*	Epidermis development	2.5	69.1	0.0470
caly	*calcyon neuron-specific vesicular protein*	Dopamine receptor signaling pathway	2.4	15.8	0.0498
dhrs11b	*dehydrogenase/reductase (SDR family) member 11b,*	Oxidoreductase activity [MF]	2.3	68.6	0.0331
si:ch211171h4.3 ¤	*serine/threonine-protein kinase SBK1 #*	Activation of MAPK activity	2.3	37.8	0.0203
limch1a	*LIM and calponin homology domains 1a*	Actomyosin structure organization	2.2	48.3	0.0277
ryr1a	*ryanodine receptor 1a*	Calcium ion transmembrane transport	2.2	583.9	0.0022
dhx32a	*DEAH (Asp-Glu-Ala-His) box polypeptide 32a*	mRNA splicing, via spliceosome	2.2	35.4	0.0274
coro6	*coronin 6*	Actin cytoskeleton organization	2.1	57.7	0.0357
slc22a16	*solute carrier family 22 member 16*	Transmembrane transport	2.1	194.3	0.0244
msh3	*mutS homolog 3(E. coli)*	Mismatch repair	2.1	49.8	0.0200
ambra1a	*activating molecule in beclin-1-regulated autophagy*	Skeletal muscle fiber development	2.1	156.1	0.0045

si:ch211242b18.1 ¤	*Myomegalin #*	Microtubule organizing center [CC]	2	186.4	0.0075
mpp7a	*MAGUK p55 subfamily member 7*	Adherens junction [CC]	1.9	117.1	0.0160
crot	*carnitine O-octanoyltransferase*	Fatty acid metabolic process	1.8	353.3	0.0327
si:ch211196f2.3 ¤	*mucin-3A #*	Integral component of membrane CC	1.8	51.4	0.0470
popdc3	*popeye domain-containing 3*	Skeletal muscle tissue development	1.7	264.7	0.0084
**Downregulated**					
rsph3	*radial spoke 3 homolog*	No evidence	4.0	29.9	0.0013
crygm2d17	*crystallin, gamma M2d17*	Visual perception	3.4	24.5	0.0033
hspa1b	*heat shock protein family A (Hsp70) member 1B*	Chaperone cofactor-dependent protein refolding	3.4	123.1	0.00005
crybb1	*crystallin, beta B1 (Fragment)*	Visual perception	3.3	109.6	0.0011
fetub	*fetuin B*	Negative regulation of endopeptidase activity	3.2	105.7	0.0064
opn1sw2	*opsin-1, short-wave-sensitive 2*	G-protein coupled receptor signaling pathway	3.2	19.8	0.0033
crygm2d1	*crystallin, gamma M2d1*	Visual perception	3.1	23.2	0.0045
crygm2d13	*crystallin, gamma M2d13*	Visual perception	3.1	49.8	0.0045
cyt1	*type I cytokeratin, enveloping layer*	Structural molecule activity [MF]	3.1	59.9	0.0016
desi1a	*desumoylating isopeptidase 1a*	Protein modification by small protein removal	3.1	71.6	0.0064
ifitm1	*interferon induced transmembrane protein 1*	Response to biotic stimulus	3.1	209.8	0.0001
opn1lw2	*red-sensitive opsin-2*	G-protein coupled receptor signaling pathway	3.1	32.4	0.0014
prelid3b	*PRELI domain containing 3B(PRELID3B))*	Phospholipid transport	3.1	777.9	0.0007
crygm2d12	*crystallin, gamma M2d12*	Visual perception	3.0	38.0	0.0218

crygm2d21	*crystallin, gamma M2d21*	Visual perception	3.0	16.6	0.0110
crygm2d7	*crystallin gamma EM2-7*	Visual perception	3.0	27.0	0.0219
fgfbp2a	*fibroblast growth factor-binding protein 2a*	Growth factor binding [MF]	3.0	9.2	0.0200
slc12a10.3	*solute carrier family 12 member 10, tandem duplicate 3*	Ion transport	3.0	27.0	0.0050
apobb.1	*apolipoprotein Bb, tandem duplicate 1*	Cholesterol biosynthetic process	2.9	75.6	0.0084
cryba1b	*crystallin, beta A1b*	Visual perception	2.9	69.6	0.0042
crybgx	*crystallin beta gamma X*	Visual perception	2.9	15.3	0.0071
crygm2d15	*crystallin, gamma M2d15*	Visual perception	2.9	18.3	0.0151
hbae3	*hemoglobin alpha embryonic-3*	Oxygen transport	2.9	43.5	0.0045
rho	*rhodopsin*	G-protein coupled receptor signaling pathway	2.9	45.8	0.0151
apoa1b	*apolipoprotein A-Ib*	Cholesterol biosynthetic process	2.8	192.4	0.0302
atf3	*activating transcription factor 3*	Regulation of transcription by RNA polymerase II	2.8	44.1	0.0223
capn3a	*calpain 3a*	Negative regulation of apoptotic process	2.8	10.0	0.0116
ccl34b.4	*chemokine (C-C motif) ligand 34b, duplicate 4*	Inflammatory response	2.8	82.3	0.0219
crybb1l1	*crystallin, beta B1,-like 1*	Visual perception	2.8	92.3	0.0116
crygm2d14	*crystallin, gamma M2d14*	Visual perception	2.8	8.3	0.0162
crygm2d20	*crystallin, gamma M2d20*	Visual perception	2.8	23.5	0.0327
crygm2d8	*crystallin, gamma M2d8*	Visual perception	2.8	32.8	0.0084
crygn2	*crystallin, gamma N2*	Visual perception	2.8	62.4	0.0151
gngt2b	*guanine nucleotide-binding protein subunit gamma*	G-protein coupled receptor signaling pathway	2.8	19.5	0.0327
ngs	*notochord granular surface*	Notochord morphogenesis	2.8	14.6	0.0151
rspo3	*R-spondin 3*	Wnt signaling pathway	2.8	21.1	0.0188

serpina1	*serpin peptidase inhibitor, clade A (alpha-1 antiproteinase, antitrypsin), member 1*	Negative regulation of endopeptidase activity	2.8	18.2	0.0212
slc32a1	*solute carrier family 32 (GABA vesicular transporter), member 1*	Amino acid transmembrane transport	2.8	6.8	0.0226
apoa1a	*apolipoprotein A-1a*	Cholesterol biosynthetic process	2.7	145.9	0.0365
arr3a	*arrestin 3, retinal (X-arrestin), like*	Signal transduction	2.7	26.8	0.0033
chia.2	*chitinase, acidic.2*	Chitin metabolic process	2.7	41.3	0.0378
gnb3b	*guanine nucleotide binding protein (G protein), beta polypeptide 3b*	Signal transduction	2.7	22.5	0.0033
myhz1.1	*myosin, heavy polypeptide 1.1, skeletal muscle*	Somite specification	2.7	381.6	0.0488
mylpfb	*myosin light chain, phosphorylatable, fast skeletal muscle b*	Calcium ion binding ^{MF}	2.7	70.2	0.0212
opn1mw1	*green-sensitive opsin-1*	G-protein coupled receptor signaling pathway	2.7	27.6	0.0219
syt5b	*synaptotagmin Vb*	Regulation of calcium ion-dependent exocytosis	2.7	8.8	0.0178
tfa	*serotransferrin*	Ion transport	2.7	111.4	0.0161
col2a1a	*collagen type II, alpha 1*	Extracellular matrix structural constituent ^{CC}	2.6	111.4	0.0244
crybb1l2	*crystallin, beta B1,-like 2*	Visual perception	2.6	109.4	0.0045
crygm2d2	*crystallin, gamma M2d2*	Visual perception	2.6	29.9	0.0295
crygm2d3	*crystallin, gamma M2d3*	Visual perception	2.6	25.5	0.0031
crygmxl2	*crystallin, gamma MX,-like 2*	Visual perception	2.6	27.1	0.0346
krtt1c19e	*keratin type 1 c19e*	No evidence	2.6	112.1	0.0219
odc1	*odc1 protein*	Ornithine decarboxylase activity	2.6	50.1	0.0425
sncb	*beta-synuclein*	Dopaminergic neuron differentiation	2.6	23.1	0.0470
ucp1	*uncoupling protein 1*	Cellular response to estrogen stimulus	2.6	87.7	0.0197
arrdc3b	*arrestin domain-containing 3b*	Protein transport	2.5	556.3	0.0255

col9a2	*procollagen, type IX, alpha 2*	Extracellular matrix organization	2.5	30.7	0.0327
cryba1l1	*beta A1-2-crystallin*	Visual perception	2.5	76.8	0.0354
cryba2b	*betaA2-2-crystallin*	Visual perception	2.5	40.4	0.0212
opn1sw1	*opsin SWS-1*	G-protein coupled receptor signaling pathway	2.5	57.8	0.0197
pck1	*phosphoenolpyruvate carboxykinase 1 (soluble)*	Polyamine biosynthetic process	2.5	22.3	0.0256
rbp4l	*retinol binding protein 4, like*	Transport	2.5	23.1	0.0002
crygm2d18	*crystallin, gamma M2d18*	Visual perception	2.4	17.8	0.0480
cyt1l	*type I cytokeratin, enveloping layer,-like*	No evidence	2.4	42.2	0.0478
gnat2	*guanine nucleotide binding protein (G protein), alpha transducing activity polypeptide 2*	Signal transduction	2.4	29.3	0.0488
col9a3	*collagen, type IX, alpha 3*	Extracellular matrix organization	2.3	21.7	0.0224
fosl2	*fos-like antigen 2*	Regulation of transcription, DNA-templated	2.3	72.9	0.0425
gbp1	*guanylate-binding protein 1*	Cell-cell signaling	2.3	39.3	0.0367
nupr1	*nuclear protein 1*	Cellular response to estrogen stimulus	2.2	89.7	0.0480
pdlim1	*PDZ and LIM domain 1*	Actin cytoskeleton organization	2.1	162.6	0.0084
si:dkey183i3.5 ¤	*thread keratin alpha #*	Intermediate filament [CC]	2.1	56.5	0.0022
arrdc2	*arrestin domain-containing 2*	Protein transport	2.0	894.2	0.0256
mrpl20	*mitochondrial ribosomal protein L20*	Translation	2.0	382.6	0.0425
mrps10	*mitochondrial ribosomal protein S10*	Translation	2.0	278.8	0.0100
si:ch211217a12.1 ¤	*alanine aminotransferase 2-like #*	Catalytic activity	2.0	980.6	0.0002
atp1b1a	*sodium/potassium-transporting ATPase subunit beta*	Ion transport	1.9	936.9	0.0045
ciapin1	*cytokine induced apoptosis inhibitor 1*	Apoptotic process	1.9	94.7	0.0335
rmdn1	*regulator of microtubule dynamics 1*	No evidence	1.9	356.4	0.0061

timm17a	*translocase of inner mitochondrial membrane 17A*	Intracellular protein transport,	1.9	638.3	0.0431
cox10	cytochrome c oxidase assembly factor heme A	Heme biosynthetic process	1.8	424.9	0.0084
alas1	*5-aminolevulinate synthase (Fragment)*	Heme biosynthetic process	1.7	1129.7	0.0045
fkbp3	*peptidylprolyl isomerase*	Protein folding	1.7	1149.6	0.0313
mrpl42	*mitochondrial ribosomal protein L42*	Mitochondrial large ribosomal subunit [CC]	1.7	239.2	0.0019
pmpcb	*peptidase (mitochondrial-processing) beta*	Proteolysis	1.7	346.9	0.0461
sumo2b	*small ubiquitin-related modifier 2*	Protein sumoylation	1.7	666.1	0.0137
tmem258	*transmembrane protein 258*	Protein N-linked glycosylation	1.7	281.9	0.0378
wbp1	*WW domain-binding protein 1*	No evidence	1.7	654.5	0.0496
gapdh	*glyceraldehyde-3-phosphate dehydrogenase*	Glucose metabolic process	1.6	237422.9	0.0498
mpc1	*mitochondrial pyruvate carrier*	Mitochondrial pyruvate transport	1.6	1410.3	0.0084
mrpl47	*mitochondrial ribosomal protein L47*	Mitochondrial translation	1.6	408.6	0.0238
ndufb4	*NADH:ubiquinone oxidoreductase subunit B4*	NADH dehydrogenase (ubiquinone) activity [MF]	1.6	2822.8	0.0123
ppifb	*peptidyl-prolyl cis-trans isomerase*	Protein folding	1.6	359.4	0.0470
psmd3	*proteasome (Prosome, macropain) 26S subunit, non-ATPase, 3*	Regulation of protein catabolic process	1.6	721.0	0.0327
rbp4	*retinol binding protein 4(plasma)*	Transport	1.6	6787.5	0.0488
tmem11	*transmembrane protein 11, mitochondrial*	Mitochondrion organization	1.6	494.9	0.0498
reep5	*receptor expression-enhancing protein*	Integral component of membrane [CC]	1.5	1166.6	0.0349
si:dkey44g23.5 ¤	*MAPK regulated corepressor interacting protein 2 #*	No evidence	1.5	255.9	0.0346

* Selected GO biological process terms presented here unless specified as [CC] for cellular component or [MF] for molecular function where there is no biological process GO terms related to the gene

¤ Official symbol provided by zebrafish nomenclature committee

Preferred name indicated in NCBI

Supplementary Table 4. List of selected differentially expressed genes in zebrafish muscle fed with the wheat diet compared to their counterparts fed with fishmeal

**Gene symbol**	**Gene name**	**Selected GO terms***	**Fold change**	**Base Mean**	**q-value**
**Upregulated**					
mhc1uba	*major histocompatibility complex class I UBA*	Antigen processing and presentation	18	1424.7	4.01E-30
si:ch211-242b18.1 ¤	*Myomegalin #*	Microtubule organizing center [CC]	8.6	226.5	0.0001
si:ch211-278j3.3 ¤	*RING-type E3 ubiquitin transferase #*	Protein ubiquitination	7	27.1	0.0158
ttll2	*tubulin tyrosine ligase-like family*	Cellular protein modification process	3.2	4.5	0.0353
raver2	*ribonucleoprotein*	mRNA splicing, via spliceosome	2.7	88.8	0.0008
btc	*betacellulin*	Epidermal growth factor receptor signaling pathway	2.6	54.8	0.0084
msh3	*mutS homolog 3 (E. coli)*	Mismatch repair	2.2	54.1	0.0222
**Downregulated**					
rsph3	*radial spoke 3*	No evidence	11	40.3	0.016
elmo2	*engulfment and cell motility 2*	Cytoskeleton organization	10.5	37.2	0.001
tfa	*serotransferrin*	Ion transport	8.7	147.6	0.005
atf3	*activating transcription factor 3*	Regulation of transcription by RNA polymerase II	8.6	55.7	0.026
fabp10a	*fatty acid-binding protein 10-A*	Transport	7.8	77.3	0.022
zbtb16a	*zinc finger and BTB domain containing 16a*	Protein ubiquitination	6.2	224.7	0.008
angel1	*angel homolog 1*	3'-5'-exoribonuclease activity [MF]	6.2	161.6	0.013
pdlim1	*PDZ and LIM domain 1*	Actin cytoskeleton organization	4.3	199.2	0.016

hsp90aa1.1	*heat shock protein 90, alpha (cytosolic), class A member 1, tandem duplicate 1*	Skeletal myofibril assembly	3.9	7086.2	0.013
aimp1b	*aminoacyl tRNA synthetase complex interacting multifunctional protein 1b*	RNA binding [MF]	3.5	2659.0	0.013
hspa1b	*heat shock protein family A (Hsp70) member 1B*	Chaperone cofactor-dependent protein refolding	7	164.2	0.012

* Selected GO biological process terms presented here unless specified as [CC] for cellular component or [MF] for molecular function where there is no biological process GO terms related to the gene

¤ Official symbol provided by zebrafish nomenclature committee

Preferred name indicated in NCBI
